# Long-term microglia depletion impairs synapse elimination and auditory brainstem function

**DOI:** 10.1038/s41598-022-23250-5

**Published:** 2022-11-02

**Authors:** Sima M. Chokr, Giedre Milinkeviciute, Gisselle A. Jimenez, Hakeem Abubakr, Karina S. Cramer

**Affiliations:** Department of Neurobiology and Behavior, University of California, Irvine, Irvine, CA 92697 USA

**Keywords:** Developmental biology, Neuroscience

## Abstract

Specialized sound localization circuit development requires synapse strengthening, refinement, and pruning. Many of these functions are carried out by microglia, immune cells that aid in regulating neurogenesis, synaptogenesis, apoptosis, and synaptic removal. We previously showed that postnatal treatment with BLZ945 (BLZ), an inhibitor of colony stimulating factor 1 receptor (CSF1R), eliminates microglia in the brainstem and disables calyceal pruning and maturation of astrocytes in the medial nucleus of the trapezoid body (MNTB). BLZ treatment results in elevated hearing thresholds and delayed signal propagation as measured by auditory brainstem responses (ABR). However, when microglia repopulate the brain following the cessation of BLZ, most of the deficits are repaired. It is unknown whether this recovery is achievable without the return of microglia. Here, we induced sustained microglial elimination with a two-drug approach using BLZ and PLX5622 (PLX). We found that BLZ/PLX treated mice had impaired calyceal pruning, diminished astrocytic GFAP in the lateral, low frequency, region of MNTB, and elevated glycine transporter 2 (GLYT2) levels. BLZ/PLX treated mice had elevated hearing thresholds, diminished peak amplitudes, and altered latencies and inter-peak latencies. These findings suggest that microglia are required to repopulate the brain in order to rectify deficits from their ablation.

## Introduction

Sound localization requires specialized synapses that ensure fast, precise and reliable signal transmission. An essential component is the projection from globular bushy cells (GBC) in the anteroventral cochlear nucleus (AVCN) to the contralateral medial nucleus of the trapezoid body (MNTB). GBC axons terminate in the calyx of Held, a large encapsulating excitatory synapse that allows rapid and secure neurotransmission. MNTB neurons in turn relay glycinergic inhibition to several brainstem nuclei, including the ipsilateral lateral superior olive (LSO). LSO neurons simultaneously receive excitatory input from spherical bushy cells in the ipsilateral VCN. The balance of excitatory and inhibitory inputs allows for the computation of interaural intensity differences^1–6^.

Several factors contribute to the maturation of this specialized circuitry. Circuit development entails the removal of excess synapses and the strengthening of the remaining intact connections. In the brainstem, spontaneous GBC firing patterns during early development are associated with elimination of small inputs in the first few postnatal days until a single dominant input is established just after hearing onset, at about postnatal day (P) 12^7–11^.

Maturing calyces are surrounded by glial cells, including microglia, astrocytes, and oligodendrocytes^12,13^. Microglia, the immune cells of the brain, can be detected in the MNTB by P6 and peak in density by P14^12,13^. In addition, the mature astrocyte marker glial fibrillary acidic protein (GFAP) peaks in expression levels just after hearing onset^12,13^. We previously showed that microglia act as key regulators of synapse elimination and circuit maturation during the development of the auditory brainstem^9,14,15^. Elimination of microglia with administration of BLZ945 (BLZ), a colony-stimulating factor 1 receptor (CSF1R) inhibitor, during the first week of development led to retention of excess calyceal inputs and diminished GFAP levels at P14^9^. Remarkably, as microglia repopulate the brain after cessation of BLZ treatment, most of the deficits seen from microglia elimination recover^15^. Prior to microglial return to the MNTB, calyceal pruning deficits were observed up to P21^15^. Once microglia fully repopulated the MNTB by 4 weeks of age (4 wk), monoinnervation of MNTB cells was restored^15^. GFAP expression recovered after prolonged microglial repopulation at 7 wk^15^. Further, temporary loss of microglia in the first postnatal week resulted in increased hearing thresholds and peak latencies, and diminished peak amplitudes in the auditory brainstem response (ABR) of the adult mouse^15^. ABR deficits seen at 4 wk largely recovered by 7 wk as well^15^. While these studies show that microglial repopulation is correlated with resumption of development, it is not known whether anatomical and functional circuit recovery is possible in the absence of microglial repopulation.

In this study we investigated the effects of long-term CSF1R inhibition on the developing auditory brainstem. We developed a dual-drug approach to ablate microglia from early postnatal development until testing at 4 or 7 weeks of age. We tested whether loss of microglia affects monoinnervation of MNTB neurons in the adult mouse. We further investigated the effects of long-term microglia depletion on distributions of excitatory and inhibitory synaptic markers and on maturation of astryocyte markers in MNTB. Auditory brainstem function was assessed using the ABR. We found that long-term CSF1R inhibition impaired calyceal pruning and the elimination of GLYT2 in the MNTB. Levels of astrocytic markers were not affected by long-term microglia depletion. ABR measurements showed that CSF1R inhibition led to increased hearing thresholds, diminished peak amplitudes, and delayed peak latency and interpeak latencies. Together, these data affirm the importance of microglia in the context of synapse elimination within the developing brainstem and highlight the reliance of auditory function on microglia.

## Materials and methods

### Animals and procedures

We used C57/BL6 mice of both sexes at 1 wk (*n* = 3), 2 wk (*n* = 4), 3 wk (*n* = 4), 4 wk (*n* = 36), and 7 wk (*n* = 29). All animal procedures were performed in accordance with the standards and approval of the Institutional Animal Care and Use Committee (IACUC) at the University of California, Irvine, and the study is reported in accordance with ARRIVE guidelines.

Mice were reared in a standard day/light cycle and received food and water ad libitum. Mice were housed in groups with no more than 5 adult mice per cage. Litters remained with the nursing dam until at least P21. Animals chosen for each treatment group were randomized and contained a mix of males and females. Animal care staff were unaware of allocation groups unless veterinary care attention was required, and mice were handled and monitored in the same way regardless of treatment. The administrator of the drugs was not blinded due to the treatment method involved. All assessments following treatment were blinded to the person collecting and analyzing data.

### Treatment

Animals received subcutaneous injections of BLZ (MW: 398.48, MedChem Express HY-12768/CS-3971), a small molecule inhibitor of CSF1R^16^, dissolved in dimethylsulfoxide (DMSO; D136-1; Fisher Scientific) to eliminate microglia as previously described^9^. Injections of 0.01 ml of BLZ solution (200 mg/kg) or control (DMSO) were administered subcutaneously every 2 or 3 days, depending on treatment group, starting from P2 with the last injection delivered at P12 or P15. Groups receiving an additional CSF1R inhibitor were fed a diet containing PLX5622 (PLX) or the respective control rodent diet ad libitum (provided by Plexxikon Inc. and formulated in AIN-76A standard chow by Research Diets Inc, 1200 mg/kg chow)^17^. Litters were given PLX or the respective control (CTL) through the milk of the lactating mother. The mother was given PLX or CTL when the litter was P2, the same day that BLZ945 or DMSO injections were administered to pups. The mother remained on the chow until the litter was of age to wean (P21), and pups had access to the PLX or control chow when they began a solid diet (~ P16). After weaning, BLZ/PLX treated mice remained on the PLX chow until testing at either 4 or 7 weeks of age.

### Neuronal tracing

In four-week-old mice, calyceal innervation levels were determined by sparsely labeling calyces of Held using dye electroporation injections as previously described^9,14,15^. Mice were transcardially perfused with artificial cerebrospinal fluid (aCSF; 130 mM NaCl, 3 mM KCl, 1.2 mM KH2PO4, 20 mM NaHCO3, 3 mM HEPES, 10 mM glucose, 2 mM CaCl2, 1.3 mM MgSO4 infused with 95% O2 and 5% CO2). Brains were quickly dissected and placed in a chamber with oxygenated aCSF. Brains remained in oxygenated aCSF for about 30 min and were then transferred to an aCSF-containing petri dish. A pulled glass micropipette was filled with rhodamine dextran amine (RDA; MW 3000, Invitrogen; in solution of 6.35% RDA with 0.4% Triton-X100 in PBS). RDA was electrophoresed at the rate of 5 pulses/second at 55 V for 50 ms using an Electro Square Porator (ECM830; BTX); multiple pulses were delivered at the midline to target the ventral acoustic stria (VAS). These pulses resulted in sparse dye-labeling of GBC axons and their calyceal terminals in MNTB on both sides. Brains were placed back into the aCSF chamber for approximately 2 h under continuous oxygenation to allow for dye transport. The tissue was then transferred to 4% PFA solution overnight followed by incubation in 30% sucrose solution in 0.1 M PBS. Brainstems were dissected and coronally cryosectioned at 18 µm in a series of 5 alternating slides. Tissue sections containing RDA-labeled calyces were immunolabeled for vesicular glutamate transporter 1/2 (VGLUT1/2) and counterstained with fluorescent Nissl.

### Determination of mono- or polyinnervation

Sections containing RDA-filled calyces were imaged using confocal microscopy (Leica SP8, 40X oil objective, zoom: 2.37, pinhole: 0.43). Tile images containing the entire MNTB in each section were taken and merged with 5% image overlap. Next, z-stack images of Nissl, RDA, and VGLUT1/2 were acquired at a resolution of 1024 × 1024, with a z-step size of 0.32 µm. Gain and offset were adjusted separately if the intensity was noticeably different in comparison with other sections on the same slide.

Image stacks were reconstructed and analyzed using Imaris software (version 9.3.1; Bitplane). RDA-filled calyces were assessed for quality by ensuring that each calyx was fully visible within the z-stack and had a visible preterminal axon segment. Calyces were reconstructed using the surface module with 0.2–0.4 surface detail to capture an accurate depiction of calyx processes shape, and 0.4 background subtraction settings. Calyceal surface area and volume were measured.

Mono- or polyinnervation status of the MNTB neuron was determined by visualizing the RDA-filled calyx with a Nissl-stained MNTB cell. MNTB cells contacted by an RDA-filled calyx, without VGLUT1/2 immunolabeling surrounding non-calyceal spaces, were designated as monoinnervated. Cells were classified as polyinnervated if VGLUT1/2 labeling was present outside of the RDA-filled calyx surrounding the same MNTB cell. Neurons were only considered polyinnervated if the VGLUT1/2 immunolabel outside the RDA-labeled calyx occupied 25% or more of the MNTB cell^9^. We compared the percentage of mono- versus polyinnervated neurons in control and microglia-depleted groups (Supplementary Table 1).

### Immunolabeling

For tissue harvesting, animals were perfused transcardially with chilled 0.9% saline followed by 4% paraformaldehyde while mice were under deep anesthesia with inhalation anesthetic isoflurane (Patterson Veterinary, item # 07–894-6867). Brains were extracted and submerged in 4% paraformaldehyde overnight at 4ºC, followed by 30% sucrose solution until cryosectioning. Serial sections were collected at 20 µm thickness over a series of six gelatin-embedded slides. Sections were stored at 4ºC until immunolabeling was performed. All immunolabeling experiments for protein expression assessment included control and treated groups at both time points. Tissue was stained for ionized-calcium binding adaptor protein-1 (IBA1), S100ß, vesicular glutamate transporter 1/2 (VGLUT1/2), vesicular GABA transporter (VGAT), glycine transporter (GLYT2), and glial fibrillary acidic protein (GFAP). Mounted brain sections containing MNTB were surrounded by hydrophobic PAP pen barrier and kept on a slide warmer for 10 min until dry. Sections were rinsed with 0.1 M phosphate buffer saline (PBS) for 10 min then incubated in 0.1% sodium dodecyl sulfate in PBS for antigen retrieval (5 min) and rinsed with PBS. Next, sections were incubated in blocking solution containing 5% normal goat serum (NGS; Vector Laboratories S-1000) and 0.3% Triton X-100 (Acros 9002–93-1) in 0.1 M PBS for 1 h at room temperature, followed by overnight incubation in blocking solution containing the primary antibodies. The tissue was then rinsed with PBS and incubated for 90 min in blocking buffer containing goat anti-rabbit, anti-guinea pig, or anti-chicken secondary antibody tagged with an Alexa (Invitrogen) fluorophore. Sections were then washed with PBS and incubated in blue fluorescent Nissl stain (NeuroTrace 530/615 or 435/455, Life Technologies N21482 or N21479) diluted 1:200 in 0.3% Triton X-100 in 0.1 M PBS. Sections were rinsed with PBS and coverslipped with Glycergel mounting medium (Dako C0563). The list of primary and secondary antibodies used in this study and their concentrations are listed in Supplementary Table 2.

### Fluorescent imaging and analysis

Sections immunolabeled for IBA1 were visualized and imaged with a slide scanner, Zeiss AxioScan.Z1 equipped with a Colibri camera (Zeiss, Oberkochen, Germany) and Zen AxioScan 2.3 software. Sections that were labeled and analyzed were imaged at 20 × magnification using a Zeiss Axioskop-2 microscope, an Axiocam camera, and Axiovision software. We analyzed the MNTB or LSO on both sides of the midline. For each animal in our analysis, at least three sections were included per primary antibody stain. Images were exported and analyzed using FIJI imaging software. The stack for the Nissl channel was used to guide outlines for the MNTB or LSO using the ROI function, and these outlines were then used to calculate percent coverage in the ROI of the channel containing the primary antibody stain as previously described^15^. Statistical analyses for immunolabeling results are presented in Supplementary Table 3.

### Auditory brainstem recordings

Auditory brainstem recordings (ABRs) were tested on 13 control and 16 BLZ/PLX treated mice at four weeks of age and 20 control and 19 BLZ/PLX treated mice at seven weeks of age. ABR recording procedures were performed as previously described^14,15^. Mice were anesthetized with ketamine (75 mg/kg i.m., KetaVed, VEDCO) and xylazine (15 mg/kg i.m., AnaSed, NADA #139–236). Body temperature was maintained at 35 °C via a far infrared warming pad (Kent Scientific, RT-0501) and sterile ocular lubricant (Puralube Vet Ointment, 006PHM02-1–8) was applied on the eyes. We inserted three pin electrodes subcutaneously with the positive, negative, and ground electrodes at the vertex, right cheek, and back near the right leg, respectively. The electrodes were connected to Tucker-Davis Technologies (TDT) RA4PA 4-channel Medusa amplifier, which was connected to a TDT RA16 Medusa Base Station. The ABR was performed in a sound-attenuating chamber (102 × 98 × 81 cm, Industrial Acoustics Company). Click and pure tone stimuli were generated using the TDT SigGen software version 4.4. Sound was presented with the TDT MF1 Multi-Function Speaker through an ear tube inserted in the animal’s left ear, with stimuli repeated 500 times at a rate of 21 stimuli per second. The stimuli were emitted using the TDT RP2.1 enhanced real time processor and the sound level was controlled with the TDT PA5 programmable attenuator. The recorded responses were amplified by the TDT SA1 stereo power amp and filtered through BioSig software version 4.4. Each sample ABR was recorded for 12 ms in response to 100 µs click or 3 ms pure tone stimuli (4, 8, 12, 16, 24, 32 kHz) and decreasing sound intensities (5 dB SPL steps from 80 to 10 dB SPL). An averaged response was computed at each sound level and was used for ABR analysis.

### ABR analysis

We assessed the ABR for hearing threshold, peak latency, interpeak latency, and peak amplitude. Threshold was defined as the lowest sound intensity at which peak I level (µV) was ≥ 4 standard deviations above the noise level^15,18^. Peaks were manually detected and labeled by a blinded observer using BioSig, and data were exported for analysis. Peak latency was determined as the time from stimulus onset to the apex of the peak. Interpeak latency was calculated as the difference between peak latencies between peaks I-II, II-III, III-IV, I-III, and I-IV. Peak amplitude was determined as the change in microvolts (µV) between the preceding trough and the apex of the subsequent peak. All ABR mean, standard error, and statistics are presented in Supplementary Table 4.

### Experimental design and statistical analysis

Multiple litters were used for each experimental group. Littermate controls were not used for experiments requiring PLX or its control because all the pups in the cage were exposed to the same treatment through lactation and later had access to the same chow. Quantitative results are presented as the mean ± SEM. All statistical analyses were performed using Prism Software (v9.3.1; GraphPad Software). Comparisons between treatment (control and BLZ/PLX) and/or age groups (four and seven weeks of age) were made using either two-way ANOVA with Sidak’s multiple comparisons test or three-way ANOVA with Tukey’s multiple comparisons test unless otherwise indicated and any missing values were handled in accordance with the appropriate statistical test (Supplementary Table 4). Statistical significance was accepted at *p* < 0.05.

## Results

### Long-term microglia depletion

We previously optimized methods for neonatal microglia elimination. Postnatal treatment with BLZ945 (BLZ) from P2-P10 eliminated microglia in the brainstem until P18, when microglia began to slowly repopulate the brainstem in a lateral-to-medial sequence^15^. Cessation of BLZ treatment at P10 led to full microglial repopulation by 4 weeks^15^. In this study, our goal was to test the effects of long-term microglial depletion in auditory brainstem development. Therefore, we developed a method to remove microglia from an early postnatal stage until the day of testing.

**Figure 1:**
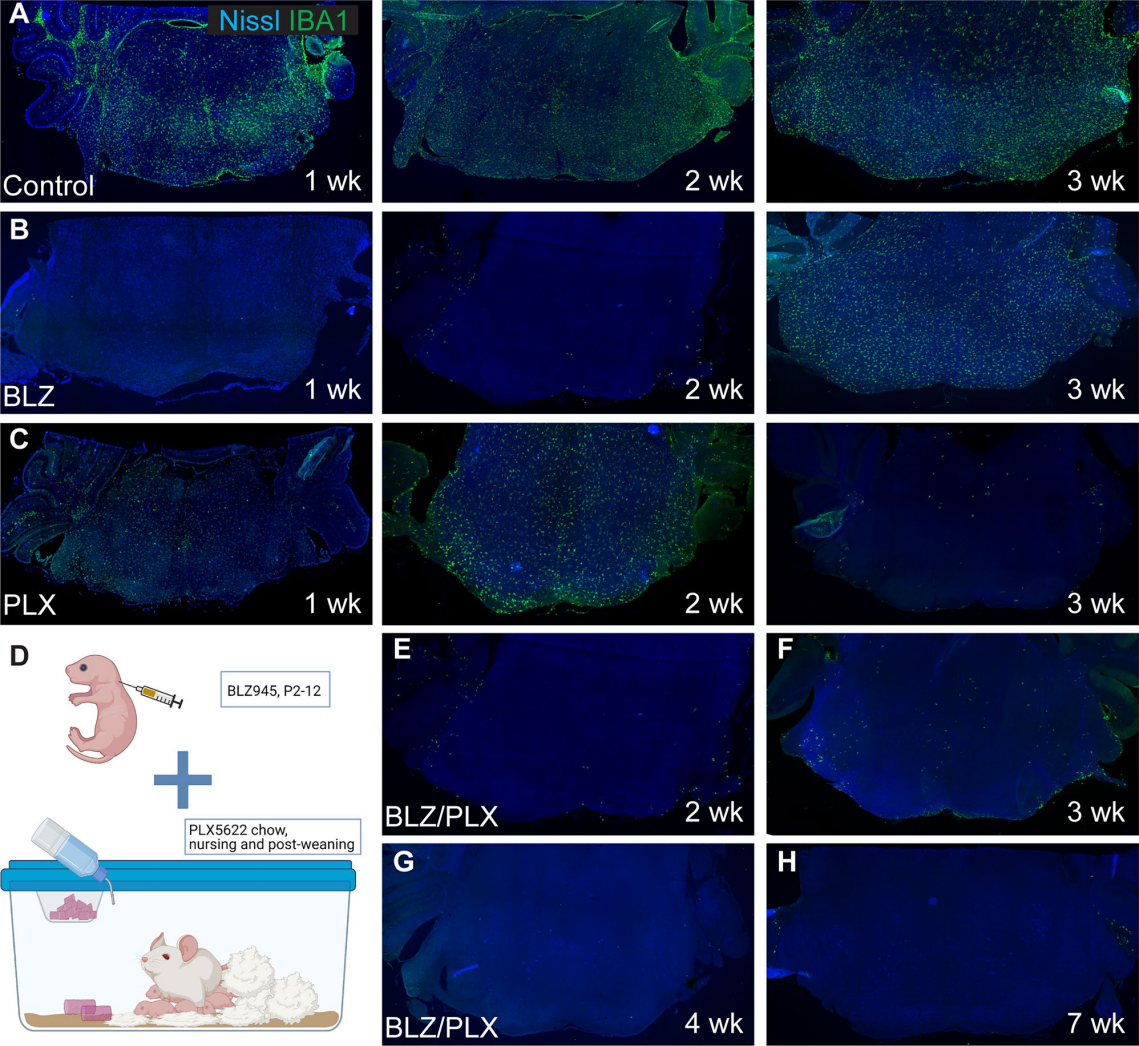
Long-term microglial depletion with CSF1R inhibitors. (**A**) Coronal brainstem section from a one-week-old (1 wk) control mouse with Nissl (blue) and microglial marker IBA1 (green) immunolabeling. Microglia are dispersed throughout the brainstem and are abundant by 2 wk. At 3 wk microglia remain distributed throughout the brainstem. (**B**) Brainstem sections from 1 wk, 2wk, and 3 wk mice treated every other day with BLZ945 (BLZ). At 1 and 2 wk, microglia are depleted. At 3 wk, microglia repopulated the brainstem despite continued BLZ treatment. (**C**) IBA1 immunolabeled section from a 1 wk mouse that received PLX5622 (PLX) through lactation beginning on P2. At 1 and 2 wk following lactation mediated PLX treatment, microglial are present throughout the brainstem but at lower cell density compared to controls. At 3 wk, pups are weaned and consume the PLX chow directly, resulting in microglial elimination with only a few IBA1 + cells detected. (**D**) Illustration of a dual drug method which involved subcutaneous BLZ injections and PLX treatment through the nursing dam. Created with BioRender.com. (**E**)**–**(**H**) Nissl and IBA1 immunolabeled sections from BLZ/PLX treated mice at 2, 3, 4, and 7 wk, respectively. Microglia were eliminated and did not return within the ages tested.

We first tested whether prolonged administration of BLZ past P10 can prevent microglial repopulation. We treated mice with subcutaneous injections of BLZ every two days beginning at P2 until perfusion at 1, 2, or 3 weeks of age, and monitored IBA1 expression levels at each endpoint. In the control group, microglia were present at each timepoint (Fig. 1A). At 1 and 2 weeks, mice treated with BLZ showed very sparse IBA1 expression in the brainstem, and microglia were not detected in the MNTB, LSO, or VCN (Fig. 1B). By 3 weeks, microglia were present throughout the brainstem, although the density of microglia appeared lower than that of controls (Fig. 1B). These data showed that BLZ treatment every other day eliminated microglia during the first 2 weeks, but did not produce long-term elimination.

Previous studies using adult mice showed that inhibition of CSF1R through the oral administration of PLX5622 (PLX) largely depleted microglia throughout the brain^17^. To test whether PLX treatment could ablate microglia in pre-weaned postnatal mice, we fed nursing dams PLX chow ad libitum once pups were P2 to initiate drug exposure through breast milk. Pups began to eat the PLX chow by P16 and remained on the drug until testing. At 1 and 2 weeks of age, PLX-treatment partially eliminated microglia, where microglial density appeared diminished throughout the brainstem (Fig. 1C). This indicates that PLX delivery through lactation reduces but does not fully eliminate microglia from the brainstem. Microglia were sparsely detected in PLX-treated mice at 3 weeks, once pups were fully weaned (Fig. 1C). Together, these data suggest that early maternal PLX treatment produces limited and variable levels of microglia depletion in pups, but that PLX is effective when administered orally to pups. We therefore tested whether a dual-drug approach may be sufficient for the long-term removal of microglia.

We combined these approaches for early and late microglia elimination. We treated mice with BLZ every other day from P2-P12 and placed the nursing mother on PLX chow when CSF1R inhibition was initiated. Pups had access to PLX or control chow before weaning and remained on the chow after weaning, until testing and brain collection (Fig. 1D). We collected brains of BLZ/PLX treated mice at 2, 3, 4, and 7 wk and found that this dual-drug approach depleted microglia from the brainstem, with very few IBA1-positive cells found in peripheral regions at each timepoint tested (Fig. 1E–H). At 7 wk, IBA1 staining patterns were only detected on the lateral and ventral edges of the section, and the auditory nuclei did not show any IBA1-positive cells (Fig. 1H). Qualitative observation indicated that mice treated with BLZ/PLX were generally smaller in size, appeared to have either smaller or absent front teeth, dome shaped heads, and seizures were occasionally observed. Survival rates from BLZ/PLX treatment at P12 was 76%, at 4 wk was 60%, and at 7 wk was 54%, compared to controls which had survival rates of 99% at P12, 91% at 4 wk, and 70% at 7 wk.

Here, we established a pharmacological approach for continuous microglial ablation throughout development. These findings demonstrate that sustained inhibition of CSF1R is attainable with a combination of subcutaneous injections of BLZ and oral delivery of PLX. BLZ/PLX treatment is effective in eliminating microglia through postnatal development, and continuous treatment with PLX prevents microglial repopulation. This method allowed us to investigate the role of microglia during the development and maturation of brainstem circuitry. Lack of microglia was confirmed for each BLZ/PLX animal used for the remainder of the study. Any BLZ/PLX-treated subject that showed IBA1-positive staining throughout the brainstem was excluded from the study.

### Calyceal pruning remains disrupted in BLZ/PLX treated mice

The establishment of a 1:1 calyx-MNTB cell ratio is dependent on microglia. Mice treated with BLZ every other day from P2-10 have higher levels of polyinnervation at P13, just after hearing onset^9^. Microglial return rectifies the pruning deficit but only after microglia fully occupy the MNTB at 4 wk^9^. Here, we tested the effects of sustained microglial elimination on calyceal pruning. We sparsely labeled calyces of both the right and left MNTB by injecting RDA into the ventral acoustic stria at the midline^9,14,15^. On the same tissue, we immunolabeled for VGLUT1/2, which prominently labels calyces in the MNTB. Cells contacted by RDA-labeled calyces were used for confocal imaging and 3D reconstruction on Imaris software.

**Figure 2:**
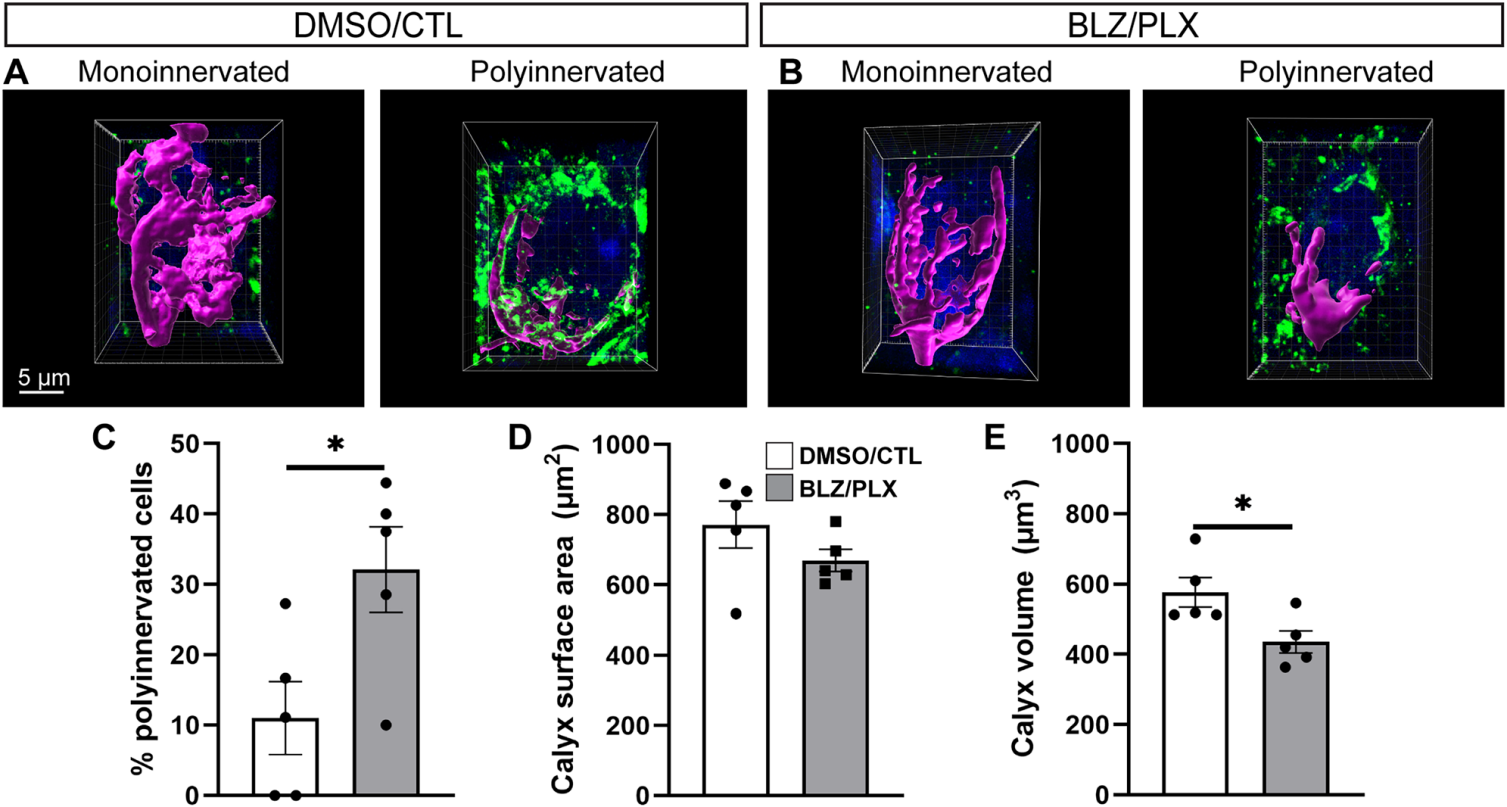
BLZ/PLX treatment disrupts calyceal elimination. (**A**) Mono- and polyinnervated MNTB neurons from a control mouse at 4 wk. 3D reconstructed rhodamine dye labeled calyx (magenta) and VGLUT1/2 (green) immunolabeling. (**B**) Mono- and polyinnervated neuron from a BLZ/PLX treated mouse at 4 wk. (**C**) Comparison of the percentage of polyinnervated versus monoinnervated cells in each treatment group showed that BLZ/PLX treatment results in more polyinnervated cells. (**D**) Surface area of the reconstructed calyces did not show a treatment-related difference. (**E**) Volume of the reconstructed calyces was lower in the BLZ/PLX treated group. *p < 0.05. Scale bar = 5 µm, applies to panels (**A**), (**B**).

We reconstructed and analyzed calyces from 5 BLZ/PLX treated mice and 5 DMSO/CTL treated mice at 4 wk. Mono- and polyinnervated MNTB cells were identified in both control and treated groups. MNTB cells were assessed for VGLUT1/2 immunolabeling surrounding the cell (see Materials and Methods) in order to determine whether the cell was contacted by more than one calyx (DMSO/CTL 11.01 ± 5.19% of cells (mean ± SEM for all data presented), BLZ/PLX 32.10 ± 6.10% of cells, Supplementary Table 1, Fig. 2A,B). We determined the percentage of mono- vs polyinnervated cells in each animal and found that BLZ/PLX treated mice had significantly increased levels of polyinnervated calyces (*p* = 0.0307, t = 2.633, df = 7.799, Welch’s t-test, Supplementary Table 1, Fig. 2C). Next, we assessed the surface area of RDA-labeled calyces in the DMSO/CTL (771.4 ± 67.14 µm^2^) and BLZ/PLX (669.8 ± 31.58 µm^2^) treated groups. Microglial depletion did not significantly affect calyceal surface area (*p* = 0.2226, t = 1.369, df = 5.687, Welch’s t-test) (Supplementary Table 1, Fig. 2D). We then tested whether microglial depletion affects calyceal volume. We found that compared to DMSO/CTL mice (576.8 ± 42.17 µm^3^, BLZ/PLX-treated mice (435.7 ± 31.59 µm^3^) had significantly reduced calyx volume (*p* = 0.0300, t = 2.677, df = 7.414, Welch’s t-test) (Supplementary Table 1, Fig. 2E). In summary, calyx pruning and maturation of calyx size were impaired in the absence of microglia.

### Effects of long-term microglial ablation on astrocyte marker levels

Neural circuit development includes multipartite signaling between microglia, neurons, and astrocytes. Microglial removal diminishes astrocytic GFAP expression at P13^9^. However, cessation of BLZ treatment at P10 leads to recovery of GFAP levels by 7 weeks of age^9^. Here, we tested whether astrocyte marker expression is affected by prolonged CSF1R inhibition.

**Figure 3:**
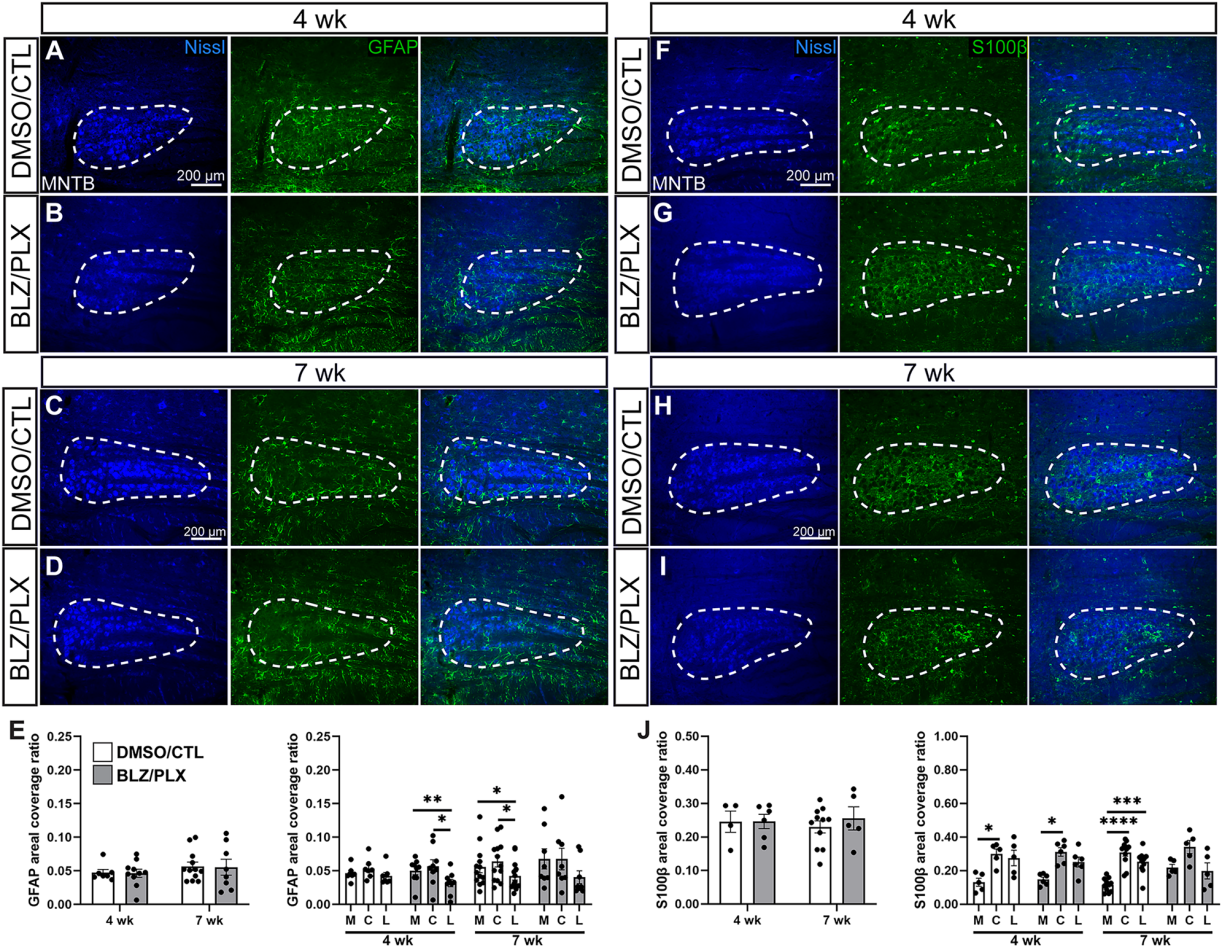
Effects of BLZ/PLX treatment on astrocytes are limited. (**A**) Images of Nissl (blue), GFAP (green), and an overlay in DMSO/CTL treated mice at 4 wk. White dashed lines indicate MNTB. (**B**) Nissl and GFAP immunolabeling in BLZ/PLX at 4 wk. (**C**) Nissl and GFAP immunolabeling in 7 wk controls. (**D**) Nissl and GFAP expression in 7 wk BLZ/PLX mice. (**E**) Comparison of overall GFAP areal coverage ratio and medial, central and lateral GFAP coveragein the MNTB of 4 and 7 wk mice. Overall treatment effects were not detected. BLZ/PLX mice at 4 wk showed less GFAP coverage in lateral and central MNTB, compared to the medial region, while DMSO/CTL mice did not show region specificity. At 7 wk, DMSO/CTL mice had lower GFAP expression levels in lateral compared to medial and central regions. BLZ/PLX did not show a regional difference at 7 wk. (**F**) Images of Nissl (blue), S100β (green) immunolabeling with an overlay in DMSO/CTL treated mice at 4 wk. (**G**) Nissl and S100β staining in BLZ/PLX mice at 4 wk. (**H**) Nissl and S100β immunolabeling in 7 wk DMSO/CTL mice. I) Nissl and S100β immunolabeled sections from BLZ/PLX mice at 7 wk. (**J**) Comparison of overall and regional S100β areal coverage ratio in the MNTB of 4 and 7 wk mice. Treatment effects were not detected. Both DMSO/CTL and BLZ/PLX treated mice at 4 wk showed higher S100β levels in central MNTB compared to the medial region. At 7 wk, regional differences in the DMSO/CTL group were evident, while the BLZ/PLX group did not show any differences. *p < 0.05. Scalebars = 200 µm, apply to panels (**A**)**–**(**D**) and (**G**)**–**(**J**).

GFAP levels were analyzed based on coverage ratio in the MNTB. We examined 7 DMSO/CTL and 10 BLZ/PLX mice at 4 wk, and 12 DMSO/CTL and 8 BLZ/PLX at 7 wk. At four weeks of age, GFAP expression appeared abundant in the MNTB of both DMSO/CTL (0.0472 ± 0.005, Supplementary Table 3, Fig. 3A) and BLZ/PLX (0.046 ± 0.006, Supplementary Table 3, Fig. 3B) groups. GFAP expression levels remained comparable across treatment groups by 7 weeks of age (DMSO/CTL 0.057 ± 0.006, Fig. 3C, BLZ/PLX 0.055, ± 0.012, Fig. 3D). We did not detect any differences across treatment groups or age or any interactions between treatment and age (Supplementary Table 3, Fig. 3E). GFAP distribution across the MNTB mediolateral axis was uniform in the DMSO/CTL group at 4 wk (medial (M)–central (C) *p* = 0.5996, C–Lateral (L) *p* = 0.3111, M–L *p* = 0.9883, Supplementary Table 3, Fig. 3E). In contrast, a significant regional difference in expression was present in the BLZ/PLX treated mice at 4 wk (M–C *p* = 0.9711, C–L *p* = 0.0269, M–L *p* = 0.0058) where the medial (0.050 ± 0.004) and central (0.053 ± 0.006) regions of MNTB had higher levels of GFAP expression compared to the lateral region (0.042 ± 0.005, Supplementary Table 3, Fig. 3E). By 7 wk, DMSO/CTL mice showed a similar regional difference in GFAP expression in the MNTB (M–C *p* = 0.8829, C–L *p* = 0.0211, M–L *p* = 0.0454) where the medial (0.056 ± 0.008) and central (0.064 ± 0.008) regions had higher GFAP expression levels compared to the lateral (0.042 ± 0.006) MNTB. Differences across regions at 7 wk in the BLZ/PLX treated group were not detected. However, there were no significant differences between the regional differences in the BLZ/PLX group at 4 wk and 7 wk (4 wk m–7 wk m *p* = 0.9879, 4 wk c–7 wk c *p* = 0.9805, 4 wk l–7 wk l *p* = 0.9993, Supplementary Table 3, Fig. 3E). Therefore, it appears that although overall GFAP expression levels do not differ across treatment groups at 4 or 7 wk, BLZ/PLX-treated mice have lower levels of GFAP expression in the lateral (low frequency) regions of the MNTB.

Next, we tested whether the astrocyte marker S100β^19^ is affected by BLZ/PLX treatment. S100β expression was abundant throughout the MNTB of both control and BLZ/PLX treated mice at 4 (DMSO/CTL 0.246 ± 0.032, BLZ/PLX 0.247 ± 0.021) and 7 wk (DMSO/CTL 0.230 ± 0.018, BLZ/PLX 0.256 ± 0.034, Fig. 3F–I). We did not detect any overall differences in expression based on treatment or age (*p* = 0.5592, Supplementary Table 3, Fig. 3J). S100β coverage was significantly lower in the medial regions of MNTB of the DMSO/CTL (0.131 ± 0.024) and BLZ/PLX (0.148 ± 0.014) groups compared to the central regions at 4 wk (DMSO/CTL M–C *p* = 0.0172, C–L *p* = 0.9998, M–L *p* = 0.5136; BLZ/PLX M–C *p* = 0.019, C–L *p* = 0.2129, M–L *p* = 0.2586, Supplementary Table 3, Fig. 3J). At 7 wk, DMSO/CTL mice showed significantly diminished levels of S100β in the medial region of MNTB (M–C *p* < 0.0001, C–L *p* = 0.1135, M–L *p* = 0.0007, Supplementary Table 3, Fig. 3J). The BLZ/PLX group at 7 wk did not show any significant differences across regions or in comparison to the 4 wk BLZ/PLX treated group (4 wk M–7 wk M *p* = 0.3018, 4 wk C–7 wk C *p* = 0.9998, 4 wk L–7 wk L *p* = 0.9946, Supplementary Table 3, Fig. 3J). These results show that S100β is not altered as a result of microglial depletion.

These experiments showed that GFAP expression was diminished in the lateral regions of MNTB in the BLZ/PLX treated group. Apart from this finding, we found that astrocyte marker expression levels in MNTB are largely unchanged by BLZ/PLX treatment. GFAP and S100β were previously shown to have some overlapping expression^9^. However, S100β is expressed earlier than GFAP^13^ and overlap in expression may be limited to astrocytes undergoing a change in maturational state.

### Synaptic protein alterations in BLZ/PLX mice

Calyces are glutamatergic and provide the dominant inputs to MNTB principal cells. Despite the calyceal pruning impairment from microglial depletion, VGLUT1/2 and VGAT levels were not altered in the MNTB of P13 BLZ-treated mice^9^. Interestingly, at P14 loss of the microglial fractalkine receptor CX3CR1 led to significantly elevated glycine transporter 2 (GLYT2) levels in the MNTB^14^. These findings provide evidence for microglial effects on synaptic pruning. Here, we tested whether prolonged microglial ablation affects levels of excitatory and inhibitory synaptic proteins in both the MNTB and the LSO.

**Figure 4:**
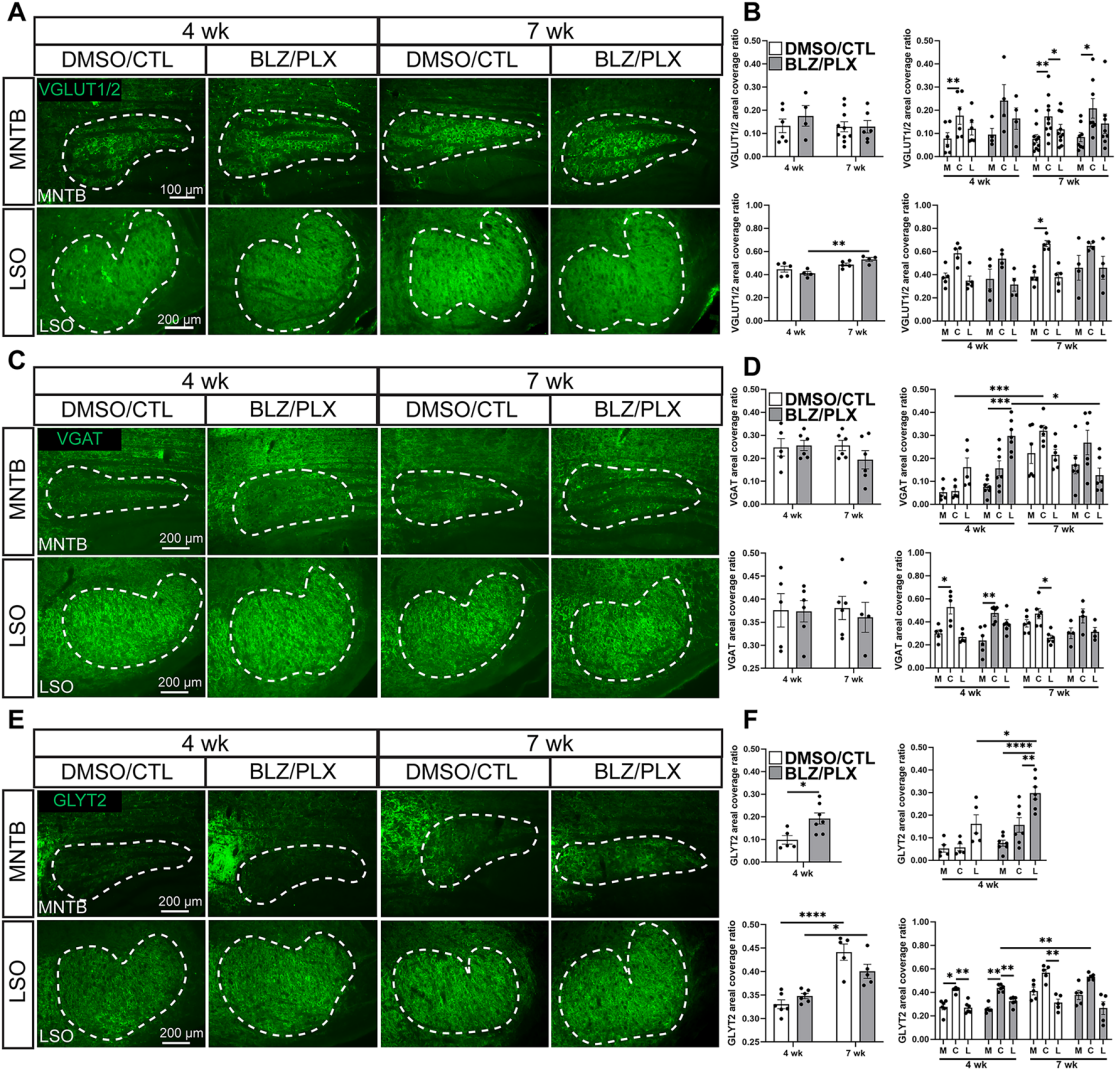
Inhibitory, but not excitatory, synaptic markers are elevated in microglia-depleted mice. (**A**) VGLUT1/2 immunolabeling (green) and their overlay in a control or treated mice at 4 and 7 wk. White dashed lines indicate MNTB or LSO. (**B**) Comparison of overall and regional VGLUT1/2 areal coverage in MNTB (above) or LSO (below) of 4 and 7 wk mice. VGLUT1/2 levels in MNTB were not altered from BLZ/PLX treatment. BLZ/PLX mice showed an increase in VGLUT1/2 levels with age. Increased VGLUT1/2 levels in central LSO were detected in the 7 wk DMSO/CTL group. (**C**) VGAT (green) immunolabeling and their overlay in control or treated mice at 4 or 7 wk. White dashed lines indicate MNTB or LSO. (**D**) comparison of overall or regional VGAT coverage in the MNTB (above) or LSO (below) of 4 and 7 wk mice. Overall VGAT levels in MNTB were not affected by microglia depletion but at 4 wk, BLZ/PLX treated mice showed higher VGAT expression in lateral MNTB. VGAT levels increased with age in both the control and treated groups. VGAT levels at 7 wk were higher in central MNTB in both DMSO/CTL and BLZ/PLX groups. There were no overall differences between age or treatment groups in the LSO and VGAT levels were elevated in central LSO at 4 wk in both control and treated groups. (**E**) GLYT2 (green) immunolabeling and their overlay in the MNTB or LSO of control or treated mice at 4 or 7 wk. White dashed lines indicate MNTB or LSO. (**F**) Comparison of GLYT2 areal coverage ratio in the MNTB of 4 7 wk mice. BLZ/PLX treated mice at 4 wk showed elevated GLYT2 levels. Lateral MNTB contained higher levels of GLYT2 in 4 wk BLZ/PLX mice. In the LSO, GLYT2 levels increased with age in both control and treated groups. GLYT2 was elevated in the central LSO in control and treated groups at 4 wk. *p < 0.05.

We examined VGLUT1/2 coverage ratio in the MNTB of 6 DMSO/CTL (0.113 ± 0.029) and 4 BLZ/PLX (0.176 ± 0.044) mice at 4 wk and 10 DMSO/CTL (0.129 ± 0.022) and 6 BLZ/PLX (0.129 ± 0.027) mice at 7 wk (Fig. 4A). Overall differences in the MNTB across treatment and age were not detected (*p* = 0.7541, Supplementary Table 3, Fig. 4B). In control mice at both 4 and 7 wk, VGLUT1/2 expression was significantly higher in central regions (4 wk 0.177 ± 0.038, 7 wk 0.173 ± 0.028) compared to the medial (4 wk 0.078 ± 0.025, 7 wk 0.077 ± 0.017) and lateral (4 wk 0.120 ± 0.027, 7 wk 0.188 ± 0.021) parts of MNTB (DMSO/CTL 4 wk M–C *p* = 0.0096, C–L *p* = 0.2462, M–L *p* = 0.1500, DMSO/CTL 7 wk M–C *p* = 0.0053, C–L *p* = 0.0155, M–L *p* = 0.1446, Supplementary Table 3, Fig. 4B). Similarly, 7 wk BLZ/PLX-treated mice showed elevated VGLUT1-2 levels in central (4 wk 0.242 ± 0.069, 7 wk 0.208 ± 0.042) regions of MNTB compared to the medial (4 wk 0.095 ± 0.027, 7 wk 0.084 ± 0.020) and lateral (4 wk 0.164 ± 0.046, 7 wk 0.142 ± 0.042) regions (4 wk M–C *p* = 0.3020, C–L *p* = 0.8876, M–L *p* = 0.8051; 7 wk M–C *p* = 0.0406, C–L *p* = 0.1575, M–L *p* = 0.7737, Supplementary Table 3, Fig. 4B). Therefore, we found that despite the permanent calyceal pruning deficits from BLZ/PLX treatment, VGLUT1/2 levels at both 4 and 7 wk were comparable to controls.

We next tested whether excitatory input is altered in the LSO in 5 DMSO/CTL (0.447 ± 0.026) and 4 BLZ/PLX (0.411 ± 0.017) treated mice and 4 wk and 5 DMSO/CTL (0.487 ± 0.013) and 4 BLZ/PLX (0.532 ± 0.017) at 7 wk (Fig. 4B). Overall VGLUT1/2 levels in the LSO did not differ between treatment groups (*p* = 0.8402, Supplementary Table 3, Fig. 4B). However, BLZ/PLX-treatment showed a significant age-related increase in VGLUT1/2 expression where higher levels of VGLUT1/2 were detected at 7 wk (0.532 ± 0.017) compared to 4 wk (0.411 ± 0.017; *p* = 0.0046, Supplementary Table 3, Fig. 4B). Regional differences were not detected in the LSO, except for a significant increase of VGLUT1/2 levels from M–C LSO (M 0.383 ± 0.031, C 0.669 ± 0.026) in the 7 wk control group (*p* = 0.025, Supplementary Table 3, Fig. 4B). Together, our data show that VGLUT1/2 expression in the MNTB and LSO is not affected by long-term CSF1R inhibition.

We measured inhibitory synapse marker VGAT coverage ratio in the MNTB of 5 DMSO/CTL (0.248 ± 0.038) and 6 BLZ/PLX (0.256 ± 0.22) mice at 4 wk, and 6 DMSO/CTL (0.285 ± 0.030) and 6 BLZ/PLX (0.194 ± 0.040) mice at 7 wk (Fig. 4C). We did not detect any differences based on treatment, age, or their interaction (*p* = 0.2731, Supplementary Table 3, Fig. 4D). At 4 wk lateral regions of MNTB in BLZ/PLX treated mice (0.298 ± 0.027) showed significantly higher levels of VGAT compared to the medial (0.076 ± 0.013) and central (0.157 ± 0.032) regions (BLZ/PLX M–C *p* = 0.1650, C–L *p* = 0.0013, M–L *p* 0.0006, Supplementary Table 3, Fig. 4D). The control group did not show regional differences across MNTB (M–C *p* > 0.9999, C–L *p* = 0.1660, M–L *p* = 0.6482, Supplementary Table 3, Fig. 4D). VGAT regional expression shifted with age, where 7 wk DMSO/CTL mice showed significantly increased levels in central MNTB (*p* = 0.0124), and BLZ/PLX mice showed significantly decreased levels in lateral MNTB (*p* = 0.052, Supplementary Table 3, Fig. 4D). At 7 wk, differences between regions of MNTB were not detected in both the control (M–C *p* = 0.2014, C–L *p* = 0.3056, M–L *p* > 0.9999) and treated groups (M–C *p* = 0.2643, C–L *p* = 00,883, M–L *p* = 0.8166, Supplementary Table 3, Fig. 4D). Taken together, VGAT expression in MNTB is not affected by age or treatment but BLZ/PLX mice appear to have higher levels of VGAT in the lateral region at 4 wk compared to controls.

We examined VGAT levels in the LSO of 5 DMSO/CTL (0.376 ± 0.036) and 6 BLZ/PLX (0.374 ± 0.023) at 4 wk, and 6 DMSO/CTL (0.381 ± 0.025) and 4 BLZ/PLX (0.361 ± 0.033) mice at 7 wk (Supplementary Table 3, Fig. 4C). Overall VGAT expression in the LSO of BLZ/PLX-treated mice was comparable to controls at both 4 and 7 wk (*p* = 0.7629, Supplementary Table 3, Fig. 4D). Further, distribution of VGAT was similar in the treated and control groups, where at 4 wk, central LSO (DMSO/CTL 0.529 ± 0.060, BLZ/PLX 0.477 ± 0.028) had significantly higher levels of VGAT than the medial (0.300 ± 0.030) region (DMSO/CTL M–C *p* = 0.0466, BLZ/PLX M–C *p* = 0.0070, Supplementary Table 3, Fig. 4D). In sum, these data show that VGAT expression in the LSO is not affected by BLZ/PLX treatment.

To further test whether inhibitory synapses are affected by loss of microglia, we examined GLYT2 expression in the MNTB of 5 DMSO/CTL (0.98 ± 0.19) and 7 BLZ/PLX (0.193 ± 0.025) mice at 4 wk, and 7 DMSO/CTL (0.004 ± 0.001) and 10 BLZ/PLX (0.004 ± 0.001) mice at 7 wk (Fig. 4E). At 4 wk, BLZ/PLX treated mice had significantly greater GLYT2 expression compared to controls (*p* = 0.0004, Supplementary Table 3, Fig. 4F). By 7wk, GLYT2 levels significantly decreased in both control (*p* = 0.0009) and treated (*p* < 0.0001) mice and there was no significant difference between the two groups (*p* = 0.6470, Supplementary Table 3, Supplementary Fig. 1). Given the observed regional difference in VGAT expression, we tested whether GLYT2 is differentially expressed across the MNTB tonotopic axis in microglia-depleted mice. In the 4 wk control group, GLYT2 expression did not significantly differ based on MNTB region (M–C *p* > 0.9999, C–L p 0.2178, M–L *p* = 0.1675, Supplementary Table 3, Fig. 4F). However, GLYT2 levels in BLZ/PLX mice significantly increased across the tonotopic axis (M–C *p* = 0.3255, C–L *p* = 0.0043, M–L *p* < 0.0001, Supplementary Table 3, Fig. 4F). Further, BLZ/PLX treated mice had significantly more GLYT2 in lateral regions compared to controls (*p* = 0.0156, Supplementary Table 3, Fig. 4F). By 7 wk, GLYT2 expression was more sparse and we did not detect differences across tonotopic regions in both control (M–C *p* = 0.6919, C–L *p* = 0.9964, M–L *p* = 0.9244) or treated (M–C *p* = 0.6859, C–L *p* = 0.8588, M–L *p* = 0.9995) groups (*p* = 0.6470, Supplementary Table 3, Supplementary Fig. 1).

Next, we assessed GLYT2 expression in the LSO of 6 DMSO/CTL (0.330 ± 0.009) and 5 BLZ/PLX (0.348 ± 0.006) mice at 4 wk and 5 DMSO/CTL (0.441 ± 0.017) and 5 BLZ/PLX (0.400 ± 0.015) mice at 7 wk (Fig. 4E). Both DMSO/CTL and BLZ/PLX treated mice showed a significant increase in GLYT2 expression with age (DMSO/CTL *p* < 0.0001, BLZ/PLX *p* = 0.0290, Supplementary Table 3, Fig. 4F). Further, regional expression of GLYT2 in central LSO significantly differed in DMSO/CTL treated mice at 4 (4 wk M–C *p* = 0.0301, C–L *p* = 0.0062, M–L *p* > 0.9999) and 7 wk (M–C *p* = 0.3382, C–L *p* = 0.0084, M–L *p* = 0.7043, Supplementary Table 3, Fig. 4F), compared to BLZ/PLX mice which showed regional differences at 4 wk in the central region (M–C *p* = 0.0084, C–L *p* = 0.0068, M–L *p* = 0.6034, Supplementary Table 3, Fig. 4F) but did not show any differences at 7 wk (M–C *p* = 0.0987, C–L *p* = 0.1015, M–L *p* = 0.9332, Supplementary Table 3, Fig. 4F). Overall, we found that microglia depleted mice had higher GLYT2 expression levels in the MNTB at 4 wk, but were comparable to control at 7 wk. In the LSO, GLYT2 levels in the central region increased with age in DMSO/CTL mice but not the BLZ/PLX group.

We found no correlation between astrocyte markers (GFAP and S100ß) and synaptic markers (VGLUT1/2, VGAT, and GlyT2) in any region of MNTB for controls or microglia-depleted mice (Spearman’s R test, *p* > 0.3 for all comparisons). This finding is consistent with the limited observation of changes in expression of astrocytic and synaptic markers and the differences in distributions of these markers.

### Mice lacking microglia have elevated hearing thresholds

**Figure 5:**
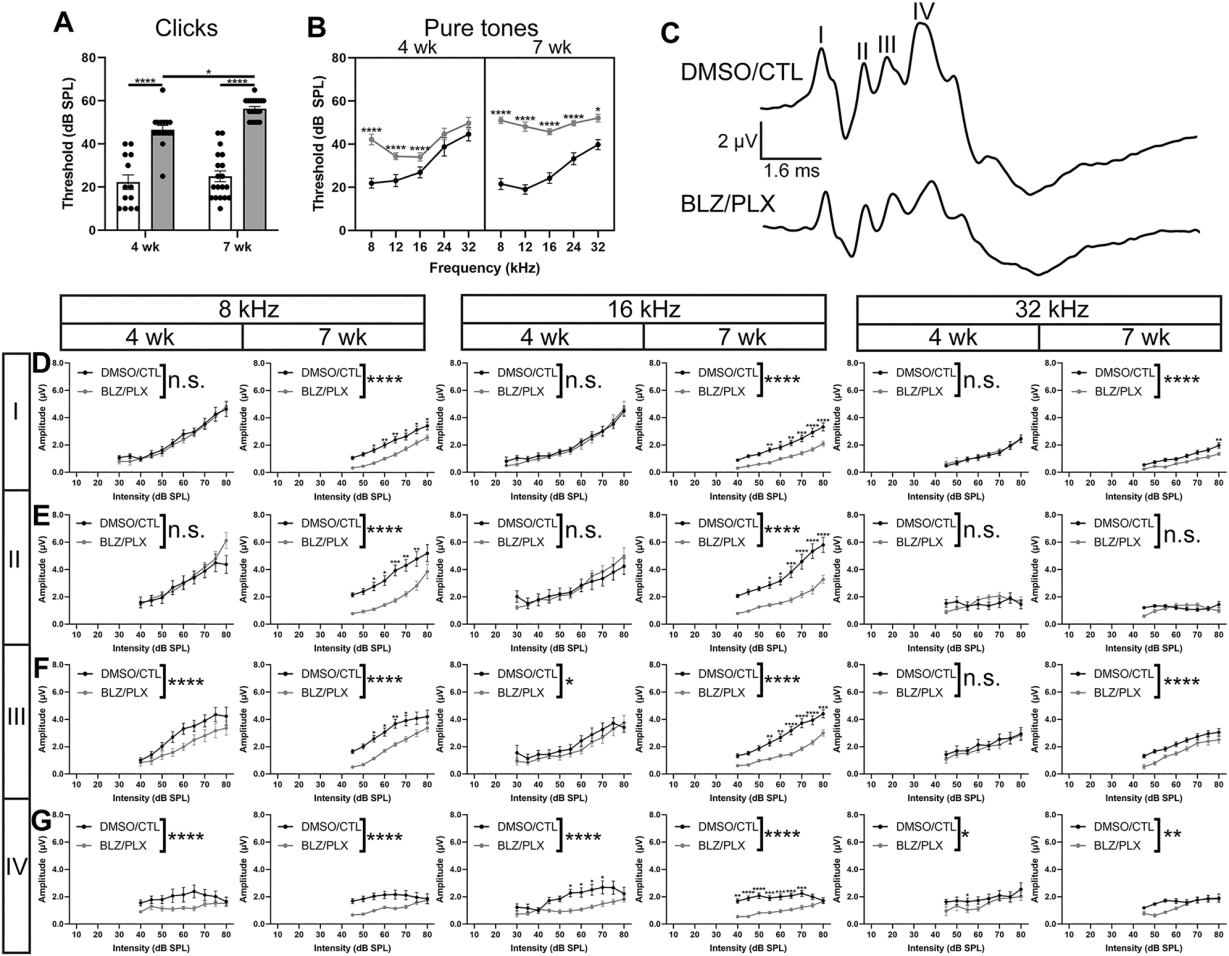
Auditory brainstem response reveals hearing impairments in BLZ/PLX treated mice. (**A**) Hearing thresholds (dB SPL) in response to click stimuli in 4 and 7 wk mice. BLZ/PLX treated mice had elevated hearing thresholds at both time points. (**B**) Hearing thresholds (dB SPL) in response to pure tone stimuli in 4 and 7 wk mice. BLZ/PLX mice at 4 wk had increased hearing thresholds in the lower frequencies compared to controls. At 7 wk, BLZ/PLX mice had elevated hearing thresholds at all frequency levels tested. (C) Example trace from a 7 wk control and treated mouse. (**D**) Peak I amplitudes (µV) in 4 and 7 wk mice at 8, 16, and 32 kHz, respectively. Peak amplitudes in BLZ/PLX mice were comparable to controls at 4 wk and diminished by 7 wk. (**E**) Peak II amplitudes in 4 and 7 wk mice at 8, 16, and 32 kHz, respectively. Peak amplitudes in BLZ/PLX mice were comparable to controls at 4 wk but diminished by 7 wk in the low and middle frequencies. (**F**) Peak III amplitudes in 4 and 7 wk mice at 8, 16, and 32 kHz, respectively. Peak amplitudes in BLZ/PLX mice were diminished in low and middle frequencies at both 4 and 7 wk of age. Peak III amplitude at 32 kHz in BLZ/PLX mice was comparable to controls at 4 wk but decreased by 7 wk. (**G**) Peak IV amplitudes in 4 and 7 wk mice at 8, 16, and 32 kHz, respectively. Peak amplitudes in BLZ/PLX mice were diminished at both 4 and 7 wk in each frequency tested. Asterisks indicate significant differences between age-matched control or treated groups. *p < 0.05.

Auditory brainstem response (ABR) measurements on 4 wk mice that temporarily lacked microglia showed increased hearing thresholds, diminished peak amplitudes, and delayed peak latencies. This impairment largely recovered following prolonged microglial repopulation at 7 wk^15^. Here, we tested whether functional recovery occurs when microglia do not return. ABRs were measured in 12 DMSO/CTL and 11 BLZ/PLX mice at 4 wk, and 20 DMSO/CTL and 13 BLZ/PLX mice at 7 wk. Click and pure tone (8, 12, 16, 24, and 32 kHz) stimuli were presented to the left ear at decreasing intensities (80–10 dB SPL, 5 dB SPL increments). Hearing threshold was determined in response to both click and pure tones and was defined as the lowest intensity at which peak I level (µV) was greater than or equal to 4 standard deviations from noise^15^. BLZ/PLX-treated mice displayed significantly elevated thresholds in response to click stimuli at both 4 and 7 wk compared to controls (4 wk *p* < 0.0001, 7 wk *p* < 0.0001, Supplementary Table 4, Fig. 5A). Hearing thresholds increased with age in the BLZ/PLX group but not the DMSO/CTL group (*p* = 0.0138, Supplementary Table 4, Fig. 5A). At 4 wk, BLZ/PLX mice showed significantly elevated hearing thresholds in response to pure tone stimuli especially in the lower frequencies (8 kHz *p* < 0.0001, 12 kHz *p* = 0.0113, 16 kHz *p* = 0.1328, 24 kHz *p* = 6475, 32 kHz *p* = 0.6278, Supplementary Table 4, Fig. 5B). By 7 wk, BLZ/PLX mice showed significantly elevated hearing thresholds, especially at lower frequencies, compared to both the 4 wk cohort and the 7 wk control group (BLZ/PLX 4 wk vs 7 wk: 8 kHz *p* = 0.0240, 12 kHz *p* < 0.0001, 16 kHz *p* = 0.0002, 24 kHz *p* = 3420, 32 kHz *p* = 0.8819; DMSO/CTL vs BLZ/PLX 7 wk: 8 kHz *p* < 0.0001, 12 kHz *p* < 0.0001, 16 kHz *p* < 0.0001, 24 kHz *p* < 0.0001, 32 kHz *p* = 0.0010, Supplementary Table 4, Fig. 5B). These results indicate that long term loss of CSF1R signaling results in a hearing impairment especially in the lower frequencies.

### Microglial depletion reduces peak latency, interpeak latency, and peak amplitude

The ABR waveform is reflective of neural activity in different parts of the ascending auditory pathway. Peak I shows activity in cochlea, spiral ganglion cells, and the VIIIth nerve, peak II corresponds with the cochlear nucleus, followed by peak III representing the superior olivary complex (SOC), a group of nuclei within the brainstem which includes the MNTB and LSO, and peak IV corresponds with activity in the lateral lemniscus^20–23^. We tested the effects of long term CSF1R inhibition on the mouse ABR at 4 and 7 weeks of age. Each marked peak was assessed for difference in amplitude (µV) between the preceding trough and the peak apex, time of each peak apex (ms), and inter-peak latency differences (ms; Fig. 5C). All ABR statistical analyses indicating peak amplitude, latency, and interpeak latency differences are shown in Supplementary Table 4.

We measured the effects of long-term microglial ablation on ABR peak amplitudes. Peak amplitude was defined as the difference in level (µV) between the apex of the peak and its preceding trough. BLZ/PLX mice at 4 wk had peak I amplitudes that were comparable to controls at each frequency tested. After prolonged depletion at 7 wk, peak I amplitudes significantly diminished at every frequency tested compared to age-matched controls (Supplementary Table 4, Fig. 5D). Similar to peak I, peak II amplitudes in 4 wk BLZ/PLX mice were comparable to controls. By 7 wk, peak II amplitudes in microglia-depleted mice significantly diminished at each frequency tested (Supplementary Table 4, Fig. 5E). At 4 wk, BLZ/PLX mice had significantly lower peak III amplitudes at 8, 12, and 16 kHz, but not the higher frequencies. By 7 wk, peak III amplitude was lower in the BLZ/PLX group at 8, 12, 16, 24, and 32 kHz (Supplementary Table 4, Fig. 5F). Peak IV amplitudes were significantly diminished at both 4 and 7 wk in BLZ/PLX treated mice (Supplementary Table 4, Fig. 5G). Together, these data show that BLZ/PLX treatment leads to diminished peak III and IV amplitudes at 4 wk, and decreased amplitudes for peaks I–IV by 7 wk.

**Figure 6:**
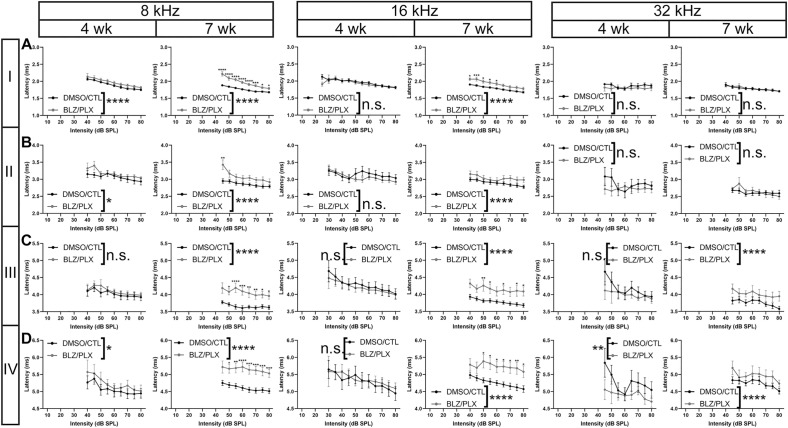
ABR peak latency is delayed with long-term microglia depletion. (**A**) Peak I latencies (ms) in 4 and 7 wk mice at 8, 16, and 32 kHz, respectively. Peak I was delayed in the low frequency at 4 and 7 wk in BLZ/PLX mice. In the middle frequency, peak I was comparable to controls at 4 wk but was delayed by 7 wk. There was no difference in peak latency at 32 kHz in the BLZ/PLX mice compared to controls. (**B**) Peak II latencies (ms) in 4 and 7 wk mice at 8, 16, and 32 kHz, respectively. At 8 kHz, peak II was delayed in BLZ/PLX mice compared to controls at both 4 and 7 wk. BLZ/PLX mice at 7 wk showed delayed peak II latency at 16 kHz. At 32 kHz, peak II was comparable to controls. (**C**) Peak III latencies in 4 and 7 wk mice at 8, 16, and 32 kHz. Peak III in BLZ/PLX mice was comparable to controls at 4 wk but was delayed in the 7 wk cohort at each frequency. (**D**) Peak IV latencies in 4 and 7 wk mice at 8, 16, and 32 kHz, respectively. Peak IV latency was delayed in 4 and 7 wk BLZ/PLX mice at 8 kHz. At 16 kHz, peak IV latency was delayed in 7 wk BLZ/PLX mice. Peak IV latency was delayed at 32 kHz in 4 and 7 wk BLZ/PLX mice. *p < 0.05.

Temporary treatment with BLZ until postnatal day 10 leads to delayed peak latencies, and this deficit largely recovers by 7 wk^15^. We assessed whether peak latency is affected by long-term BLZ/PLX treatment. Absolute peak latency was defined as the time from stimulus (ms) to the time at which the apex of each peak occurred^15^. At 4 wk, BLZ/PLX mice had delayed peak I latency at 8 and 24 kHz, while other frequencies tested were not affected (Supplementary Table 4, Fig. 6A). After prolonged CSF1R inhibition, peak I latency at 7 wk was increased at 8, 12, and 16 kHz but not the higher frequencies (Supplementary Table 4, Fig. 6A). As was seen for peak I, peak II latency in BLZ/PLX treated mice at 4 wk was delayed at 8 and 24 kHz (Supplementary Table 4, Fig. 6B). Peak II latencies at 7 wk were delayed at 8, 12, 16, and 24 kHz in BLZ/PLX treated mice (Supplementary Table 4, Fig. 6B). BLZ/PLX treatment did not affect peak III latencies at 4 wk. However, by 7 wk, peak III was delayed at each frequency tested in the BLZ/PLX mice (Supplementary Table 4, Fig. 6C). Peak IV latencies were delayed at 8, 12, 24, and 32 kHz in 4 wk BLZ/PLX mice and by 7 wk peak IV latencies were delayed at all frequencies tested (Supplementary Table 4, Fig. 6D). Overall, BLZ/PLX treatment delayed peak I-IV latencies in mostly lower frequencies at 4 wk, and by 7 wk this treatment led to delayed peak I-IV latencies at all frequencies tested.

**Figure 7:**
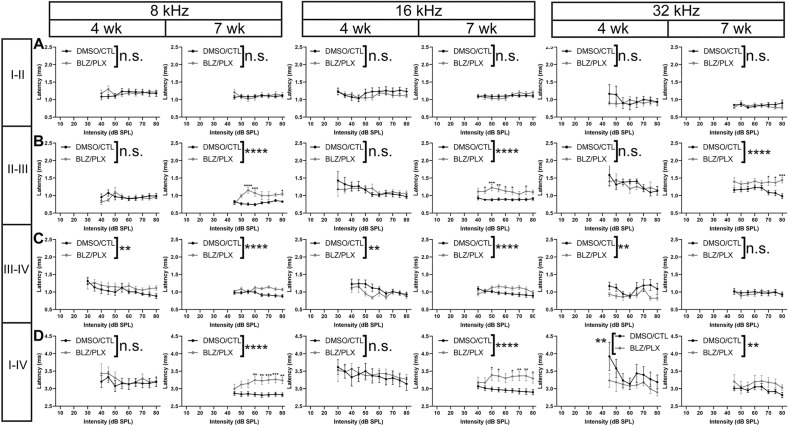
ABR interpeak latency is altered in microglia-depleted mice. (**A**) Peak I-II interpeak latencies (ms) in 4 and 7 wk mice at 8, 16, and 32 kHz, respectively. Peak I-II signal transmission was not affected by BLZ/PLX treatment. (**B**) Peak II-III interpeak latencies at 8, 16, and 32 kHz, respectively. At 7 wk, BLZ/PLX mice showed increased peak latencies at each frequency tested. (**C**) Peak III-IV interpeak latencies in 4 and 7 wk mice at 8, 16, and 32 kHz, respectively. At 8 kHz, peak III-IV latencies were longer in BLZ/PLX mice at both 4 and 7 wk. At 16 and 32 kHz, peak III-IV latencies were shortened at 4 wk. By 7 wk, 16 kHz recordings in BLZ/PLX mice showed reduced interpeak latencies and 32 kHz recordings were comparable to controls. (**D**) Peak I-IV interpeak latencies in 4 and 7 wk mice at 8, 16, and 32 kHz, respectively. At 4 wk, peak I-IV latency was shortened in BLZ/PLX mice at the highest frequency tested. BLZ/PLX mice at 7 wk showed delayed peak I-IV latency at all frequencies tested. *p < 0.05.

We measured interpeak latency differences to assess whether latency delays in BLZ/PLX treated mice were a result of peripheral conduction defects, impairments in central signaling, or both. Interpeak latency was calculated as the difference in time (ms) between the apex of a peak and the apex of the preceding peak. Both 4 and 7 wk BLZ/PLX treated mice showed diminished peak I-II interpeak latency at 24 kHz, while other frequencies tested were not affected (Supplementary Table 4, Fig. 7A). Peak II-III interpeak latency in BLZ/PLX mice was comparable to controls at 4 wk. At 7 wk, BLZ/PLX mice showed delayed peak II-III latencies at 12, 16, 24, and 32 kHz (Supplementary Table 4, Fig. 7B). Peak III-IV latency was delayed at 8 kHz in 4 wk BLZ/PLX mice but decreased at 12–32 kHz. At 7 wk, peak III-IV latency was delayed at 8, 12, and 16 kHz while higher frequencies were comparable to controls (Supplementary Table 4, Fig. 7C). We determined whether overall central latency was affected by calculating the difference between peak IV and peak I (ms). We found that peak I-IV latency was decreased at 12, 24, and 32 kHz in the 4 wk BLZ/PLX group. By 7 wk, peak I-IV latency was delayed at 8, 16, and 32 kHz but decreased at 12 and 24 kHz (Supplementary Table 4, Fig. 7D). These data show that interpeak latencies are mostly affected after prolonged CSF1R inhibition. Taken together with the absolute peak latencies, it appears that there is some latency delay at the cochlea-SGN-VIIIth nerve level, and central signaling impairments are more evident following extended microglia depletion.

## Discussion

We established a novel method for sustained microglia depletion using BLZ/PLX to ensured microglial ablation from the early postnatal stages until testing at either 4 or 7 weeks of age. Here, we showed that calyceal pruning remained impaired in the microglia-depleted mice, indicating that microglia are required for the establishment of monoinnervation in the MNTB. Astrocytic proteins GFAP and S100β were not affected by BLZ/PLX treatment. Synaptic proteins were assessed, and we found that VGLUT1/2 levels were not affected in the MNTB or LSO following microglial depletion. In 4 wk BLZ/PLX mice, inhibitory synapse marker VGAT appeared higher in the lateral regions of MNTB and GLYT2 levels were elevated throughout MNTB, especially in the lateral regions as well. Long-term CSF1R inhibition led to diminished ABR peak amplitudes, and altered peak latencies and interpeak latencies. These effects became more pronounced after prolonged microglia depletion.

### Long-term microglial depletion

Microglial elimination can be achieved with various genetic, pharmacologic, and irradiation methods^16,24–27^. This study used a dual-drug approach involving BLZ injections early in development along with PLX chow until testing. BLZ/PLX treatment led to almost complete ablation of IBA1 + microglia, apart from a few cells found in lateral regions of the brainstem. BLZ injections every other day are effective for eliminating microglia in neonates until three weeks of age, after which microglia repopulate the brainstem^15^. Placing the dam on PLX chow eliminates approximately 99% of microglia in the embryonic mouse and results in neural abnormalities^28^. In rats, intraperitoneal PLX injections in neonates are effective for microglial depletion, and testing day was performed at either P10, if treated early in development, or P50, if treatment began on P41^29^. In the mouse, PLX injections beginning on P4 were effective for microglial elimination until P15^30^. Our study required a treatment that achieved long-term microglial elimination until either four or seven weeks of age, thus mortality and treatment severity were considered during method development. Interestingly, we found that at three weeks of age, BLZ treatment alone was not sufficient for complete microglial elimination, and PLX treatment alone was not fully effective as well. These differences likely reflect developmental variations in the dependence of microglia on CSF1R and other targets of inhibition, as well as the effectiveness of drug delivery as the animal grew. BLZ945 effectively inhibits CSF1R, but may also inhibit other receptor tyrosine kinases, including c-kit^31^. PLX5562 may be more selective for CSF1R than other CSF1R inhibitors^17^. These differences may impact the efficacy on microglia elimination at different stages and highlight the importance of understanding factors needed for microglial survival at different points in development. Our combined approach allowed us to overcome these limitations.

### Microglia mediated pruning in MNTB and LSO

Emerging studies have highlighted key roles for microglia during development. Microglia are highly motile cells that eliminate synapses, promote formation of new synapses, and participate in synaptic remodeling during development and plasticity^32–35^. Microglia have been reported in several developmental systems as key regulators for the formation of appropriate circuit connectivity^33,36^. Further, microglia modulate synapse and circuit refinement largely through activity-dependent interactions.

#### Microglia regulate calyceal pruning

In the MNTB, calyx pruning and refinement prior to hearing onset is regulated by spontaneous activity^7,8,10,11^. Many of the small calyceal inputs to MNTB cells are eliminated by P6 and the 1:1 dominant input is matured around the time of hearing onset^7,8,10,37–40^. Additionally, MNTB-LSO topography is sharpened as excess inputs are eliminated and remaining synapses are strengthened before hearing onset^41–45^.

In the brainstem, microglia populate the VCN by P0 and the MNTB by P6^13^. Loss of microglia during the first week of development leads to an impairment in calyceal elimination just after hearing onset^9^. Calyceal pruning deficits can recover with microglial repopulation^15^. Here, we showed that, in the absence of microglia, calyceal elimination remains impaired. Overall levels of VGLUT1/2 did not change, suggesting that this presynaptic marker is distributed among multiple calyceal inputs. It is not known whether these inputs differ from the total input level normally seen with monoinnervation. Differences in calyx size and shape may influence input strength^46^.

#### Inhibitory synapses are affected by microglial signaling

During visual system development, GABA-receptive microglia preferentially interact with inhibitory synapses rather than excitatory synapses, with a small percentage of those microglia displaying phagocytic morphology^30^. Indeed, loss of GABA_B_ receptor signaling prevents microglia-mediated elimination of inhibitory synapses^30^. In the spinal cord, microglia alter glycinergic, but not GABAergic synapses^47^. Microglial inhibitory synapse elimination can be modulated by the MERTK signaling pathway in the hippocampus^48^. Although the MNTB is largely regulated by highly glutamatergic calyces, inhibitory input from the ventral nucleus of the trapezoid body provides glycinergic input to the MNTB which matches the large conduction of the calyceal input and can suppress MNTB spiking when activated^49,50^. MNTB cells are glycinergic and rapidly provide input to several auditory nuclei including the LSO^51–54^. In the developing MNTB, loss of the microglial fractalkine receptor CX3CR1 results in an increase in GLYT2 at P14. At nine weeks of age, GLYT2 distribution across the brainstem is altered in CX3CR1 mutant mice^14^. Here, we showed that long-term inhibition of the CSF1R signaling pathway prevents VGAT elimination in the lateral, low frequency, regions of MNTB. GLYT2 levels in the MNTB at four weeks of age were elevated after BLZ/PLX treatment, especially in the lateral regions as well. As the brain matures, GLYT2 levels in the MNTB drastically drop, the effects of microglial ablation were not detected at 7 wk^14,55^. Thus, microglia modulate inhibitory synapse development in the auditory brainstem, likely through the CX3CR1 pathway, to regulate GLYT2 levels in the auditory brainstem.

### Microglial influence on astrocyte development

The calyx of Held is contacted by multiple glial cell types, including microglia, astrocytes, and oligodendrocytes^56^. Like microglia, astrocyte signaling is imperative for proper neural circuit development^57^. During development, glial signaling is achievable with the dynamic migratory abilities of both microglia and S100β + astrocytes^19,58^. In several microglial or microglial receptor ablation paradigms, astrocyte maturation or reactivity marker GFAP expression appears to be altered, either by up or down regulation^9,14,15,59^. Further, in pathologic conditions astrocytic S100β signaling regulates microglial reactivity profiles^60^. GFAP + astrocytes are in very close contact with the calyx of Held synapse during development and are involved in gliotransmission in the mature MNTB and contact active zones of the calyx^13,61,62^. Loss of microglia during the first postnatal week leads to diminished GFAP levels, and microglial repopulation rectifies this deficit by 7 wk^9,15^. CX3CR1 mutation leads to an increase in GFAP levels at 9 wk^14^. Here, we found that GFAP levels were significantly lower in the lateral MNTB at 4 wk following long-term microglial ablation. However, we did not detect an overall change in GFAP levels in the MNTB. Additionally, S100β levels in BLZ/PLX mice were comparable to controls. These observations suggest that, in chronically microglia-depleted mice, GFAP expression returns to normal levels. Neuroglial crosstalk is highly plastic and regulatory mechanisms that elicit glial process extension and retraction, activation, and motility depend on several factors such as cytokine release or receptor activation^63^. Therefore, it is likely that long-term loss of CSF1R signaling triggers regulatory mechanisms that restore astrocyte marker levels in the MNTB.

### CSF1R inhibition effects on auditory brainstem function

Signal propagation is tonotopically organized and gradient expression of synaptic proteins, ion channels, and progression of neural size ensure the precision involved in frequency-specific and rapid signaling. Genetic loss of neuroligand CSF1 in the *Csf1*^*op/op*^ mouse model results in increased hearing thresholds in low, middle, and high frequencies in the ABR in part due to deformity of the ossicles and bone capsule of the inner ear^64^. Temporary inhibition of the microglial receptor CSF1R with BLZ results in increased hearing thresholds, decreased peak amplitudes, and altered peak and interpeak latencies, and these deficits mostly recover by 7 wk^15^. Temporary treatment with BLZ led to diminished peak amplitudes mostly in the lower frequencies at 4 wk. Peak latencies were delayed in the lower frequencies, but faster in the higher frequencies for peaks I and II^15^. Additionally, loss of microglial CX3CR1 resulted in faster peak and interpeak latencies especially in the higher frequencies^14^. In this study we found diminished amplitudes in peaks I-IV. Absolute peak latencies were delayed in lower frequencies but faster in higher frequencies. At a central level, interpeak latencies for peaks I-II were not affected at 4 or 7 wk, peaks II-III were delayed following prolonged CSF1R inhibition, and peaks III-IV in the middle to higher frequencies were faster following prolonged depletion by 7 wk. Peak I-IV interpeak latency showed delayed peak latencies at all frequencies.

Since peak II-III represents signaling between the cochlear nucleus and the superior olivary complex, which includes the MNTB^20–23^, it is evident that the signal propagation along the ventral acoustic stria is delayed as a result of microglial elimination and that this effect is propagated along the ascending auditory pathway. This finding is intriguing, as globular bushy cells are highly myelinated and this characteristic secures rapid transmission mediated by the calyx of Held^65–68^. Oligodendrocyte and NG2 cell development is regulated by microglia^69^ and, in turn, NG2 cells regulate fast-signaling characteristics of calyces^61^. Microglia regulate myelination through signaling molecules such as IGF-1 and through microglia-mediated phagocytosis^70–72^. In the brainstem, oligodendroglial BDNF regulates presynaptic properties at the calyx of Held and affects glutamatergic vesicle release^73^. Loss of BDNF results in reduced peak II-IV amplitudes in the click ABR^73^. Ablation of microglial BDNF results in impaired synapse formation and plasticity^34^. Together, these findings suggest a role for significant communication between microglia and oligodendrocytes in the developing auditory system.

## Conclusion

Development of precise neural circuitry is regulated by microglia, and some processes are not achievable without the presence of microglia. Establishment of monoinnervation by the calyx of Held is moderated by microglia. Microglia also appear to be involved in regulating inhibitory synapse levels in the auditory brainstem. Astrocyte development is impaired with short-term microglia depletion but appears to be restored after microglial repopulation or long-term depletion. These studies highlight the complex roles that microglia have in the developmental emergence of precise auditory pathways.

## Supplementary Information

Supplementary Figure S1.

Supplementary Table S1.

Supplementary Table S2.

Supplementary Table S3.

Supplementary Table S4.

## Data Availability

All of the data reported here will be made available by request to the corresponding author at cramerk@uci.edu.
