# Supplementary figures and images for: Long-term microglia depletion impairs synapse elimination and auditory brainstem function
Source: Sci Rep. 2022 Nov 2;12:18521. doi: 10.1038/s41598-022-23250-5 (PMC9630367; doi:10.1038/s41598-022-23250-5)

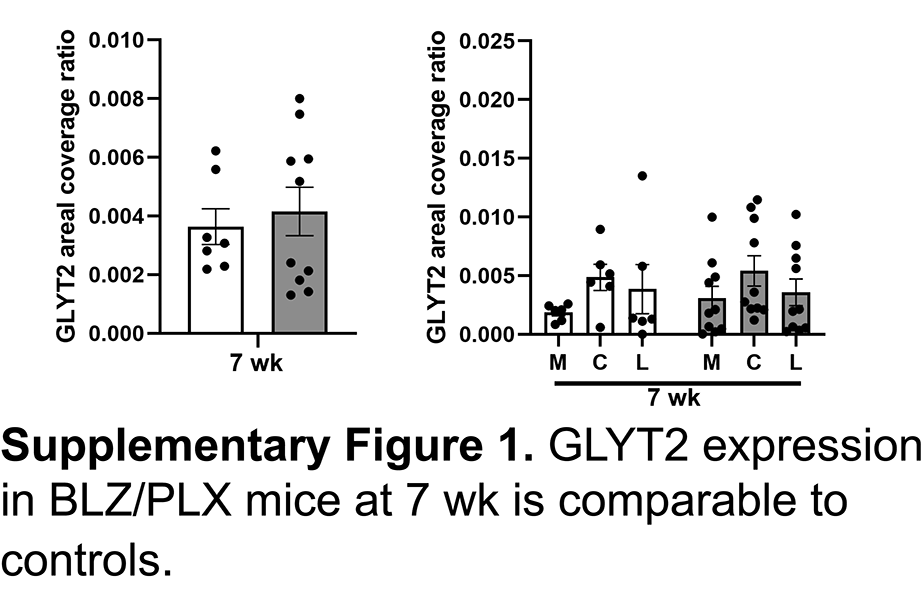

Supplement: Supplementary file 1 — Supplementary Figure S1. [file 41598_2022_23250_MOESM1_ESM.tif]
