# Supplementary material for: Long-term microglia depletion impairs synapse elimination and auditory brainstem function
Source: Sci Rep. 2022 Nov 2;12:18521. doi: 10.1038/s41598-022-23250-5 (PMC9630367; doi:10.1038/s41598-022-23250-5)
Supplement: Supplementary file 2 — Supplementary Table S1. [file 41598_2022_23250_MOESM2_ESM.pdf]

Supplementary Table 1-Calyx pruning								
	% polyinnervated cells		Calyx surface area		Calyx volume			
	DMSO/CTL	BLZ/PLX	DMSO/CTL	BLZ/PLX	DMSO/CTL	BLZ/PLX		
**Mean**	11.01	32.1	771.4	669.8	576.8	435.7		
**SEM**	5.191	6.103	67.14	31.58	42.17	31.59		
**Unpaired t test with Welch's correction**								
	**P value**	**t, df**	**P value**	**t, df**	**P value**	**t, df**		
	0.0307	t=2.633, df=7.799	0.2226	t=1.369, df=5.687	0.03	t=2.677, df=7.414		
**Analysis of 3D Reconstructed Calyces**								
**Mouse ID**	**Treatment**	**Surface Area (µm^2)**	**Volume (µm^3)**	**Mono or Polyinnervated?**	**Total # of Calyces**	**Total # of poly cells**	**Ratio of poly/total cells**	
B970	DMSOCTL	1350	1354	mono	9	1	0.111111111	
		850	394	mono				
		931	513	mono				
		957	575	mono				
		671	343	mono				
		852	437	mono				
		573	283	poly				
		1106	538	mono				
		511	183	mono				
B971	DMSOCTL	1066	723	mono	7	3	0.428571429	
		1336	969	mono				
		748	497	poly				
		916	440	mono				
		1048	585	poly				
		889	524	poly				
		529	195	mono				
B972	DMSOCTL	352	110	mono	8	0	0	
		451	361	mono				
		434	186	mono				
		493	182	mono				
		523	268	mono				
		891	333	mono				
		700	387	mono				
		306	177	mono				
B973	DMSOCTL	858	1005	mono	6	0	0	
		884	834	mono				
		828	872	mono				
		968	711	mono				
		756	497	mono				
		669	453	mono				
B964	BLZPLX	536	282	mono	5	2	0.4	
		487	335	poly				
		643	355	mono				
		856	596	mono				

		493	393	poly			
B966	BLZPLX	685	396	mono	9	4	0.444444444
		758	505	poly			
		626	419	poly			
		657	458	mono			
		798	594	mono			
		695	406	mono			
		471	275	poly			
		537	373	mono			
		539	357	poly			
B967	BLZPLX	1044	712	mono	8	3	0.375
		439	335	poly			
		943	583	mono			
		452	245	mono			
		755	632	poly			
		712	459	mono			
		802	609	mono			
		425	798	poly			
B968	BLZPLX	705	479	mono	7	2	0.285714286
		726	435	poly			
		1122	681	mono			
		660	304	mono			
		527	337	poly			
		1041	483	mono			
		681	468	mono			
B969	BLZPLX	324	210	mono	10	1	0.1
		854	362	mono			
		465	263	mono			
		562	359	mono			
		532	282	mono			
		889	453	mono			
		819	586	mono			
		664	393	mono			
		712	402	mono			
		463	330	poly			
