# Supplementary material for: Long-term microglia depletion impairs synapse elimination and auditory brainstem function
Source: Sci Rep. 2022 Nov 2;12:18521. doi: 10.1038/s41598-022-23250-5 (PMC9630367; doi:10.1038/s41598-022-23250-5)
Supplement: Supplementary file 3 — Supplementary Table S2. [file 41598_2022_23250_MOESM3_ESM.pdf]

Supplementary Table 2-List of antibodies used					
**Primary Antibodies**					
**Antigen**	**Host**	**RRID**	**Cat. No.**	**Source**	**Dilution**
IBA1	Rabbit	AB_839504	019-19741	Wako	1:500
GFAP	Chicken	AB_304558	ab4674	Abcam	1:1000
VGLUT1/2	Rabbit	AB_2285905	135 503	Synaptic Systems	1:200
VGAT	Rabbit	AB_887871	131 002	Synaptic Systems	1:200
GlyT2	Rabbit	AB_2619997	272 003	Synaptic systems	1:200
**Secondary Antibodies**					
Alexa 488	Chicken	AB_2534096	A11039	Thermo Fisher Scientific	1:500
Alexa 647	Rabbit	AB_2535812	A21244	Thermo Fisher Scientific	1:500
Alexa 488	Rabbit	AB_2633280	A32731	Thermo Fisher Scientific	1:500
