# Supplementary material for: Long-term microglia depletion impairs synapse elimination and auditory brainstem function
Source: Sci Rep. 2022 Nov 2;12:18521. doi: 10.1038/s41598-022-23250-5 (PMC9630367; doi:10.1038/s41598-022-23250-5)
Supplement: Supplementary file 4 — Supplementary Table S3. [file 41598_2022_23250_MOESM4_ESM.pdf]

Supplementary Table 3-Immunolabeling analysis						
GFAP						
**Overall**	**DMSO/CTL**			**BLZ/PLX**		
	**Mean**	**SEM**	**N**	**Mean**	**SEM**	**N**
4 wk	0.047239641	0.00474	7	0.04633	0.00607196	10
7 wk	0.05662886	0.00644	12	0.05525	0.011916193	8
**2 way ANOVA**			**Šídák's multiple comparisons test**			**P value**
	**F (DFn, DFd)**	**P value**	4 wk:DMSO/CTL vs. 4 wk:BLZ/PLX			>0.9999
Interactio	F (1, 33) = 0.0009167	P=0.9760	4 wk:DMSO/CTL vs. 7 wk:DMSO/CTL			0.9533
Age	F (1, 33) = 1.386	P=0.2474	4 wk:BLZ/PLX vs. 7 wk:BLZ/PLX			0.9629
Treatmen	F (1, 33) = 0.02158	P=0.8841	7 wk:DMSO/CTL vs. 7 wk:BLZ/PLX			>0.9999
**Regional**	**DMSO/CTL 4 wk**			**BLZ/PLX 4 wk**		
	**Mean**	**SEM**	**N**	**Mean**	**SEM**	**N**
M	0.046087627	0.0041	7	0.05004	0.006024991	9
C	0.053272011	0.00576	7	0.05671	0.009630424	9
L	0.04226233	0.00521	7	0.0317	0.005529951	9
	**DMSO/CTL 7 wk**			**BLZ/PLX 7 wk**		
M	0.055548819	0.00847	13	0.06762	0.015072773	8
C	0.06387332	0.00832	13	0.06745	0.016062794	8
L	0.042233338	0.00612	13	0.04035	0.009632178	8
**3 way ANOVA**						
		**F (DFn, DFd)**		**P value**		
Region of MNTB		F (1.942, 64.09) = 19.25		P<0.0001		
Treatment		F (1, 33) = 1.299		P=0.2626		
Age		F (1, 33) = 0.04487		P=0.8335		
Region of MNTB x Treatment		F (2, 66) = 0.8846		P=0.4177		
Region of MNTB x Age		F (2, 66) = 2.108		P=0.1295		
Treatment x Age		F (1, 33) = 0.1147		P=0.7370		
Region of MNTB x Treatment x Ag		F (2, 66) = 0.2269		P=0.7976		
**Tukey's multiple comparisons test**				**P value**		
M:DMSO/CTL 4 wk vs. M:BLZ/PLX 4 wk				>0.9999		
M:DMSO/CTL 4 wk vs. M:DMSO/CTL 7 wk				0.9952		
M:DMSO/CTL 4 wk vs. M:BLZ/PLX 7 wk				0.9393		
M:DMSO/CTL 4 wk vs. C:DMSO/CTL 4 wk				0.5996		
M:DMSO/CTL 4 wk vs. C:BLZ/PLX 4 wk				0.9932		
M:DMSO/CTL 4 wk vs. C:DMSO/CTL 7 wk				0.7355		
M:DMSO/CTL 4 wk vs. C:BLZ/PLX 7 wk				0.9586		
M:DMSO/CTL 4 wk vs. L:DMSO/CTL 4 wk				0.9883		
M:DMSO/CTL 4 wk vs. L:BLZ/PLX 4 wk				0.6357		
M:DMSO/CTL 4 wk vs. L:DMSO/CTL 7 wk				>0.9999		
M:DMSO/CTL 4 wk vs. L:BLZ/PLX 7 wk				>0.9999		
M:BLZ/PLX 4 wk vs. M:DMSO/CTL 7 wk				>0.9999		
M:BLZ/PLX 4 wk vs. M:BLZ/PLX 7 wk				0.9879		
M:BLZ/PLX 4 wk vs. C:DMSO/CTL 4 wk				>0.9999		
M:BLZ/PLX 4 wk vs. C:BLZ/PLX 4 wk				0.9711		
M:BLZ/PLX 4 wk vs. C:DMSO/CTL 7 wk				0.9614		
M:BLZ/PLX 4 wk vs. C:BLZ/PLX 7 wk				0.9924		

M:BLZ/PLX 4 wk vs. L:DMSO/CTL 4 wk		0.9958		
M:BLZ/PLX 4 wk vs. L:BLZ/PLX 4 wk		0.0058		
M:BLZ/PLX 4 wk vs. L:DMSO/CTL 7 wk		0.9981		
M:BLZ/PLX 4 wk vs. L:BLZ/PLX 7 wk		0.9985		
M:DMSO/CTL 7 wk vs. M:BLZ/PLX 7 wk		0.9997		
M:DMSO/CTL 7 wk vs. C:DMSO/CTL 4 wk		>0.9999		
M:DMSO/CTL 7 wk vs. C:BLZ/PLX 4 wk		>0.9999		
M:DMSO/CTL 7 wk vs. C:DMSO/CTL 7 wk		0.8829		
M:DMSO/CTL 7 wk vs. C:BLZ/PLX 7 wk		0.9998		
M:DMSO/CTL 7 wk vs. L:DMSO/CTL 4 wk		0.962		
M:DMSO/CTL 7 wk vs. L:BLZ/PLX 4 wk		0.4727		
M:DMSO/CTL 7 wk vs. L:DMSO/CTL 7 wk		0.0454		
M:DMSO/CTL 7 wk vs. L:BLZ/PLX 7 wk		0.9829		
M:BLZ/PLX 7 wk vs. C:DMSO/CTL 4 wk		0.9973		
M:BLZ/PLX 7 wk vs. C:BLZ/PLX 4 wk		>0.9999		
M:BLZ/PLX 7 wk vs. C:DMSO/CTL 7 wk		>0.9999		
M:BLZ/PLX 7 wk vs. C:BLZ/PLX 7 wk		>0.9999		
M:BLZ/PLX 7 wk vs. L:DMSO/CTL 4 wk		0.8756		
M:BLZ/PLX 7 wk vs. L:BLZ/PLX 4 wk		0.5634		
M:BLZ/PLX 7 wk vs. L:DMSO/CTL 7 wk		0.8884		
M:BLZ/PLX 7 wk vs. L:BLZ/PLX 7 wk		0.6367		
C:DMSO/CTL 4 wk vs. C:BLZ/PLX 4 wk		>0.9999		
C:DMSO/CTL 4 wk vs. C:DMSO/CTL 7 wk		0.9937		
C:DMSO/CTL 4 wk vs. C:BLZ/PLX 7 wk		0.9984		
C:DMSO/CTL 4 wk vs. L:DMSO/CTL 4 wk		0.3111		
C:DMSO/CTL 4 wk vs. L:BLZ/PLX 4 wk		0.3168		
C:DMSO/CTL 4 wk vs. L:DMSO/CTL 7 wk		0.9654		
C:DMSO/CTL 4 wk vs. L:BLZ/PLX 7 wk		0.9834		
C:BLZ/PLX 4 wk vs. C:DMSO/CTL 7 wk		>0.9999		
C:BLZ/PLX 4 wk vs. C:BLZ/PLX 7 wk		>0.9999		
C:BLZ/PLX 4 wk vs. L:DMSO/CTL 4 wk		0.96		
C:BLZ/PLX 4 wk vs. L:BLZ/PLX 4 wk		0.0269		
C:BLZ/PLX 4 wk vs. L:DMSO/CTL 7 wk		0.971		
C:BLZ/PLX 4 wk vs. L:BLZ/PLX 7 wk		0.9805		
C:DMSO/CTL 7 wk vs. C:BLZ/PLX 7 wk		>0.9999		
C:DMSO/CTL 7 wk vs. L:DMSO/CTL 4 wk		0.5684		
C:DMSO/CTL 7 wk vs. L:BLZ/PLX 4 wk		0.1233		
C:DMSO/CTL 7 wk vs. L:DMSO/CTL 7 wk		0.0211		
C:DMSO/CTL 7 wk vs. L:BLZ/PLX 7 wk		0.7726		
C:BLZ/PLX 7 wk vs. L:DMSO/CTL 4 wk		0.9089		
C:BLZ/PLX 7 wk vs. L:BLZ/PLX 4 wk		0.6329		
C:BLZ/PLX 7 wk vs. L:DMSO/CTL 7 wk		0.9182		
C:BLZ/PLX 7 wk vs. L:BLZ/PLX 7 wk		0.702		
L:DMSO/CTL 4 wk vs. L:BLZ/PLX 4 wk		0.9472		
L:DMSO/CTL 4 wk vs. L:DMSO/CTL 7 wk		>0.9999		
L:DMSO/CTL 4 wk vs. L:BLZ/PLX 7 wk		>0.9999		
L:BLZ/PLX 4 wk vs. L:DMSO/CTL 7 wk		0.9729		

L:BLZ/PLX 4 wk vs. L:BLZ/PLX 7 wk				0.9993		
L:DMSO/CTL 7 wk vs. L:BLZ/PLX 7 wk				>0.9999		
**S100β**						
**Overall**	**DMSO/CTL**			**BLZ/PLX**		
	**Mean**	**SEM**	**N**	**Mean**	**SEM**	**N**
4 wk	0.245526676	0.03161	4	0.24653	0.021228602	6
7 wk	0.230353531	0.01797	11	0.25564	0.034409825	5
**2 way ANOVA**			**Šídák's multiple comparisons test**			**P value**
	**F (DFn, DFd)**	**P value**	4 wk:DMSO/CTL vs. 4 wk:BLZ/PLX			>0.9999
Interactior	F (1, 22) = 0.2163	P=0.6464	4 wk:DMSO/CTL vs. 7 wk:DMSO/CTL			0.9989
Age	F (1, 22) = 0.01351	P=0.9085	4 wk:BLZ/PLX vs. 7 wk:BLZ/PLX			>0.9999
Treatment	F (1, 22) = 0.2537	P=0.6195	7 wk:DMSO/CTL vs. 7 wk:BLZ/PLX			0.9746
**Regional**	**DMSO/CTL 4 wk**			**BLZ/PLX 4 wk**		
	**Mean**	**SEM**	**N**	**Mean**	**SEM**	**N**
M	0.131038024	0.02422	5	0.14805	0.013894857	6
C	0.300431582	0.02882	5	0.31178	0.025579224	6
L	0.275758659	0.04594	5	0.25191	0.029621608	6
	**DMSO/CTL 7 wk**			**BLZ/PLX 7 wk**		
M	0.115165606	0.01191	11	0.21798	0.019908515	5
C	0.307870247	0.02197	11	0.34132	0.038861914	5
L	0.252559934	0.02018	11	0.19897	0.04786334	5
**3 way ANOVA**						
		**F (DFn, DFd)**		**P value**		
Region of MNTB		F (1.484, 34.14) = 63.39		P<0.0001		
Treatment		F (1, 23) = 0.01277		P=0.9110		
Age		F (1, 23) = 0.4369		P=0.5152		
Region of MNTB x Treatment		F (2, 46) = 2.996		P=0.0598		
Region of MNTB x Age		F (2, 46) = 5.932		P=0.0051		
Treatment x Age		F (1, 23) = 0.3512		P=0.5592		
Region of MNTB x Treatment x Age		F (2, 46) = 2.004		P=0.1464		
**Tukey's multiple comparisons test**				**P value**		
M:DMSO/CTL 4 wk vs. M:BLZ/PLX 4 wk				0.9999		
M:DMSO/CTL 4 wk vs. M:DMSO/CTL 7 wk				0.9999		
M:DMSO/CTL 4 wk vs. M:BLZ/PLX 7 wk				0.3349		
M:DMSO/CTL 4 wk vs. C:DMSO/CTL 4 wk				0.0172		
M:DMSO/CTL 4 wk vs. C:BLZ/PLX 4 wk				0.0159		
M:DMSO/CTL 4 wk vs. C:DMSO/CTL 7 wk				0.0076		
M:DMSO/CTL 4 wk vs. C:BLZ/PLX 7 wk				0.0511		
M:DMSO/CTL 4 wk vs. L:DMSO/CTL 4 wk				0.5136		
M:DMSO/CTL 4 wk vs. L:BLZ/PLX 4 wk				0.2017		
M:DMSO/CTL 4 wk vs. L:DMSO/CTL 7 wk				0.0761		
M:DMSO/CTL 4 wk vs. L:BLZ/PLX 7 wk				0.957		
M:BLZ/PLX 4 wk vs. M:DMSO/CTL 7 wk				0.7939		
M:BLZ/PLX 4 wk vs. M:BLZ/PLX 7 wk				0.3018		
M:BLZ/PLX 4 wk vs. C:DMSO/CTL 4 wk				0.0544		
M:BLZ/PLX 4 wk vs. C:BLZ/PLX 4 wk				0.0109		

M:BLZ/PLX 4 wk vs. C:DMSO/CTL 7 wk		0.0008		
M:BLZ/PLX 4 wk vs. C:BLZ/PLX 7 wk		0.0733		
M:BLZ/PLX 4 wk vs. L:DMSO/CTL 4 wk		0.4229		
M:BLZ/PLX 4 wk vs. L:BLZ/PLX 4 wk		0.2586		
M:BLZ/PLX 4 wk vs. L:DMSO/CTL 7 wk		0.0225		
M:BLZ/PLX 4 wk vs. L:BLZ/PLX 7 wk		0.9865		
M:DMSO/CTL 7 wk vs. M:BLZ/PLX 7 wk		0.0564		
M:DMSO/CTL 7 wk vs. C:DMSO/CTL 4 wk		0.0236		
M:DMSO/CTL 7 wk vs. C:BLZ/PLX 4 wk		0.0043		
M:DMSO/CTL 7 wk vs. C:DMSO/CTL 7 wk		<0.0001		
M:DMSO/CTL 7 wk vs. C:BLZ/PLX 7 wk		0.0412		
M:DMSO/CTL 7 wk vs. L:DMSO/CTL 4 wk		0.2366		
M:DMSO/CTL 7 wk vs. L:BLZ/PLX 4 wk		0.0711		
M:DMSO/CTL 7 wk vs. L:DMSO/CTL 7 wk		0.0007		
M:DMSO/CTL 7 wk vs. L:BLZ/PLX 7 wk		0.816		
M:BLZ/PLX 7 wk vs. C:DMSO/CTL 4 wk		0.5157		
M:BLZ/PLX 7 wk vs. C:BLZ/PLX 4 wk		0.28		
M:BLZ/PLX 7 wk vs. C:DMSO/CTL 7 wk		0.2055		
M:BLZ/PLX 7 wk vs. C:BLZ/PLX 7 wk		0.2006		
M:BLZ/PLX 7 wk vs. L:DMSO/CTL 4 wk		0.9739		
M:BLZ/PLX 7 wk vs. L:BLZ/PLX 4 wk		0.9951		
M:BLZ/PLX 7 wk vs. L:DMSO/CTL 7 wk		0.9757		
M:BLZ/PLX 7 wk vs. L:BLZ/PLX 7 wk		>0.9999		
C:DMSO/CTL 4 wk vs. C:BLZ/PLX 4 wk		>0.9999		
C:DMSO/CTL 4 wk vs. C:DMSO/CTL 7 wk		>0.9999		
C:DMSO/CTL 4 wk vs. C:BLZ/PLX 7 wk		0.9978		
C:DMSO/CTL 4 wk vs. L:DMSO/CTL 4 wk		0.9998		
C:DMSO/CTL 4 wk vs. L:BLZ/PLX 4 wk		0.9785		
C:DMSO/CTL 4 wk vs. L:DMSO/CTL 7 wk		0.9439		
C:DMSO/CTL 4 wk vs. L:BLZ/PLX 7 wk		0.7743		
C:BLZ/PLX 4 wk vs. C:DMSO/CTL 7 wk		>0.9999		
C:BLZ/PLX 4 wk vs. C:BLZ/PLX 7 wk		0.9998		
C:BLZ/PLX 4 wk vs. L:DMSO/CTL 4 wk		0.9996		
C:BLZ/PLX 4 wk vs. L:BLZ/PLX 4 wk		0.2129		
C:BLZ/PLX 4 wk vs. L:DMSO/CTL 7 wk		0.7825		
C:BLZ/PLX 4 wk vs. L:BLZ/PLX 7 wk		0.6505		
C:DMSO/CTL 7 wk vs. C:BLZ/PLX 7 wk		0.9991		
C:DMSO/CTL 7 wk vs. L:DMSO/CTL 4 wk		0.9998		
C:DMSO/CTL 7 wk vs. L:BLZ/PLX 4 wk		0.906		
C:DMSO/CTL 7 wk vs. L:DMSO/CTL 7 wk		0.1135		
C:DMSO/CTL 7 wk vs. L:BLZ/PLX 7 wk		0.6567		
C:BLZ/PLX 7 wk vs. L:DMSO/CTL 4 wk		0.9859		
C:BLZ/PLX 7 wk vs. L:BLZ/PLX 4 wk		0.7709		
C:BLZ/PLX 7 wk vs. L:DMSO/CTL 7 wk		0.675		
C:BLZ/PLX 7 wk vs. L:BLZ/PLX 7 wk		0.0742		
L:DMSO/CTL 4 wk vs. L:BLZ/PLX 4 wk		>0.9999		
L:DMSO/CTL 4 wk vs. L:DMSO/CTL 7 wk		>0.9999		

L:DMSO/CTL 4 wk vs. L:BLZ/PLX 7 wk				0.9791		
L:BLZ/PLX 4 wk vs. L:DMSO/CTL 7 wk				>0.9999		
L:BLZ/PLX 4 wk vs. L:BLZ/PLX 7 wk				0.9946		
L:DMSO/CTL 7 wk vs. L:BLZ/PLX 7 wk				0.9874		
**VGLUT1/2 (MNTB)**						
**Overall**	**DMSO/CTL**			**BLZ/PLX**		
	**Mean**	**SEM**	**N**	**Mean**	**SEM**	**N**
4 wk	0.132832421	0.02945	6	0.17582	0.044266352	4
7 wk	0.1289456	0.02169	10	0.12923	0.026813049	6
**2 way ANOVA**			**Šídák's multiple comparisons test**			**P value**
	**F (DFn, DFd)**	**P value**	4 wk:DMSO/CTL vs. 4 wk:BLZ/PLX			0.7905
Interaction	F (1, 22) = 0.5175	P=0.4795	4 wk:DMSO/CTL vs. 7 wk:DMSO/CTL			0.9996
Age	F (1, 22) = 0.7231	P=0.4043	4 wk:BLZ/PLX vs. 7 wk:BLZ/PLX			0.7481
Treatment	F (1, 22) = 0.5316	P=0.4736	7 wk:DMSO/CTL vs. 7 wk:BLZ/PLX			>0.9999
**Regional**	**DMSO/CTL 4 wk**			**BLZ/PLX 4 wk**		
	**Mean**	**SEM**	**N**	**Mean**	**SEM**	**N**
M	0.077836765	0.02521	6	0.09522	0.026776054	4
C	0.176894651	0.03781	6	0.24152	0.069355805	4
L	0.119791638	0.02668	6	0.16408	0.046449194	4
	**DMSO/CTL 7 wk**			**BLZ/PLX 7 wk**		
M	0.0769961	0.01747	10	0.08402	0.01950507	8
C	0.1731872	0.0275	10	0.20817	0.042018973	8
L	0.1177326	0.0212	10	0.14219	0.041825745	8
**3 way ANOVA**						
		**F (DFn, DFd)**		**P value**		
Region of MNTB		F (1.961, 47.06) = 47.85		P<0.0001		
Treatment		F (1, 24) = 0.1496		P=0.7023		
Age		F (1, 24) = 1.042		P=0.3175		
Region of MNTB x Treatment		F (2, 48) = 0.1378		P=0.8716		
Region of MNTB x Age		F (2, 48) = 1.257		P=0.2937		
Treatment x Age		F (1, 24) = 0.1004		P=0.7541		
Region of MNTB x Treatment x Age		F (2, 48) = 0.08181		P=0.9216		
**Tukey's multiple comparisons test**				**P value**		
M:DMSO/CTL 4wk vs. M:BLZ/PLX 4 wk				>0.9999		
M:DMSO/CTL 4wk vs. M:DMSO/CTL 7 wk				>0.9999		
M:DMSO/CTL 4wk vs. M:BLZ/PLX 7 wk				>0.9999		
M:DMSO/CTL 4wk vs. C:DMSO/CTL 4wk				0.0096		
M:DMSO/CTL 4wk vs. C:BLZ/PLX 4 wk				0.6039		
M:DMSO/CTL 4wk vs. C:DMSO/CTL 7 wk				0.3838		
M:DMSO/CTL 4wk vs. C:BLZ/PLX 7 wk				0.3498		
M:DMSO/CTL 4wk vs. L:DMSO/CTL 4wk				0.15		
M:DMSO/CTL 4wk vs. L:BLZ/PLX 4 wk				0.8435		
M:DMSO/CTL 4wk vs. L:DMSO/CTL 7 wk				0.9767		
M:DMSO/CTL 4wk vs. L:BLZ/PLX 7 wk				0.9589		
M:BLZ/PLX 4 wk vs. M:DMSO/CTL 7 wk				>0.9999		
M:BLZ/PLX 4 wk vs. M:BLZ/PLX 7 wk				>0.9999		

M:BLZ/PLX 4 wk vs. C:DMSO/CTL 4wk		0.802		
M:BLZ/PLX 4 wk vs. C:BLZ/PLX 4 wk		0.302		
M:BLZ/PLX 4 wk vs. C:DMSO/CTL 7 wk		0.671		
M:BLZ/PLX 4 wk vs. C:BLZ/PLX 7 wk		0.5446		
M:BLZ/PLX 4 wk vs. L:DMSO/CTL 4wk		0.9998		
M:BLZ/PLX 4 wk vs. L:BLZ/PLX 4 wk		0.8051		
M:BLZ/PLX 4 wk vs. L:DMSO/CTL 7 wk		0.9997		
M:BLZ/PLX 4 wk vs. L:BLZ/PLX 7 wk		0.9959		
M:DMSO/CTL 7 wk vs. M:BLZ/PLX 7 wk		>0.9999		
M:DMSO/CTL 7 wk vs. C:DMSO/CTL 4wk		0.4946		
M:DMSO/CTL 7 wk vs. C:BLZ/PLX 4 wk		0.5788		
M:DMSO/CTL 7 wk vs. C:DMSO/CTL 7 wk		0.0053		
M:DMSO/CTL 7 wk vs. C:BLZ/PLX 7 wk		0.2777		
M:DMSO/CTL 7 wk vs. L:DMSO/CTL 4wk		0.9509		
M:DMSO/CTL 7 wk vs. L:BLZ/PLX 4 wk		0.7909		
M:DMSO/CTL 7 wk vs. L:DMSO/CTL 7 wk		0.1446		
M:DMSO/CTL 7 wk vs. L:BLZ/PLX 7 wk		0.9275		
M:BLZ/PLX 7 wk vs. C:DMSO/CTL 4wk		0.5955		
M:BLZ/PLX 7 wk vs. C:BLZ/PLX 4 wk		0.6201		
M:BLZ/PLX 7 wk vs. C:DMSO/CTL 7 wk		0.334		
M:BLZ/PLX 7 wk vs. C:BLZ/PLX 7 wk		0.0406		
M:BLZ/PLX 7 wk vs. L:DMSO/CTL 4wk		0.9884		
M:BLZ/PLX 7 wk vs. L:BLZ/PLX 4 wk		0.8546		
M:BLZ/PLX 7 wk vs. L:DMSO/CTL 7 wk		0.9843		
M:BLZ/PLX 7 wk vs. L:BLZ/PLX 7 wk		0.7737		
C:DMSO/CTL 4wk vs. C:BLZ/PLX 4 wk		0.9973		
C:DMSO/CTL 4wk vs. C:DMSO/CTL 7 wk		>0.9999		
C:DMSO/CTL 4wk vs. C:BLZ/PLX 7 wk		>0.9999		
C:DMSO/CTL 4wk vs. L:DMSO/CTL 4wk		0.2462		
C:DMSO/CTL 4wk vs. L:BLZ/PLX 4 wk		>0.9999		
C:DMSO/CTL 4wk vs. L:DMSO/CTL 7 wk		0.9431		
C:DMSO/CTL 4wk vs. L:BLZ/PLX 7 wk		>0.9999		
C:BLZ/PLX 4 wk vs. C:DMSO/CTL 7 wk		0.9925		
C:BLZ/PLX 4 wk vs. C:BLZ/PLX 7 wk		>0.9999		
C:BLZ/PLX 4 wk vs. L:DMSO/CTL 4wk		0.8355		
C:BLZ/PLX 4 wk vs. L:BLZ/PLX 4 wk		0.8876		
C:BLZ/PLX 4 wk vs. L:DMSO/CTL 7 wk		0.8078		
C:BLZ/PLX 4 wk vs. L:BLZ/PLX 7 wk		0.9619		
C:DMSO/CTL 7 wk vs. C:BLZ/PLX 7 wk		0.9998		
C:DMSO/CTL 7 wk vs. L:DMSO/CTL 4wk		0.9456		
C:DMSO/CTL 7 wk vs. L:BLZ/PLX 4 wk		>0.9999		
C:DMSO/CTL 7 wk vs. L:DMSO/CTL 7 wk		0.0155		
C:DMSO/CTL 7 wk vs. L:BLZ/PLX 7 wk		>0.9999		
C:BLZ/PLX 7 wk vs. L:DMSO/CTL 4wk		0.8031		
C:BLZ/PLX 7 wk vs. L:BLZ/PLX 4 wk		0.9996		
C:BLZ/PLX 7 wk vs. L:DMSO/CTL 7 wk		0.7289		
C:BLZ/PLX 7 wk vs. L:BLZ/PLX 7 wk		0.1575		

L:DMSO/CTL 4wk vs. L:BLZ/PLX 4 wk				0.9972		
L:DMSO/CTL 4wk vs. L:DMSO/CTL 7 wk				>0.9999		
L:DMSO/CTL 4wk vs. L:BLZ/PLX 7 wk				>0.9999		
L:BLZ/PLX 4 wk vs. L:DMSO/CTL 7 wk				0.9935		
L:BLZ/PLX 4 wk vs. L:BLZ/PLX 7 wk				>0.9999		
L:DMSO/CTL 7 wk vs. L:BLZ/PLX 7 wk				>0.9999		
**VGLUT1/2 (LSO)**						
**Overall**	**DMSO/CTL**			**BLZ/PLX**		
	**Mean**	**SEM**	**N**	**Mean**	**SEM**	**N**
4 wk	0.4474504	0.02554	5	0.41129	0.016819338	4
7 wk	0.487425329	0.01315	5	0.53154	0.016873809	4
**2 way ANOVA**			**Šídák's multiple comparisons test**			**P value**
	**F (DFn, DFd)**	**P value**	4 wk:DMSO/CTL vs. 4 wk:BLZ/PLX			0.5655
Interaction	F (1, 14) = 4.299	P=0.0571	4 wk:DMSO/CTL vs. 7 wk:DMSO/CTL			0.4368
Age	F (1, 14) = 17.13	P=0.0010	4 wk:BLZ/PLX vs. 7 wk:BLZ/PLX			0.0046
Treatment	F (1, 14) = 0.04219	P=0.8402	7 wk:DMSO/CTL vs. 7 wk:BLZ/PLX			0.404
**Regional**	**DMSO/CTL 4 wk**			**BLZ/PLX 4 wk**		
	**Mean**	**SEM**	**N**	**Mean**	**SEM**	**N**
M	0.3776868	0.0372	5	0.36308	0.08505084	4
C	0.5857956	0.0378	5	0.53879	0.034537095	4
L	0.3489102	0.0399	5	0.31438	0.056194418	4
	**DMSO/CTL 7 wk**			**BLZ/PLX 7 wk**		
M	0.3830158	0.03065	5	0.45991	0.106769572	4
C	0.668508	0.02569	5	0.6496	0.019023357	4
L	0.3788582	0.04169	5	0.45978	0.099523012	4
**3 way ANOVA**						
		**F (DFn, DFd)**		**P value**		
Region of MNTB		F (1.218, 17.05) = 17.56		P=0.0003		
Treatment		F (1, 14) = 15.82		P=0.0014		
Age		F (1, 14) = 0.1304		P=0.7234		
Region of MNTB x Treatment		F (2, 28) = 0.1512		P=0.8603		
Region of MNTB x Age		F (2, 28) = 0.3158		P=0.7318		
Treatment x Age		F (1, 14) = 3.939		P=0.0671		
Region of MNTB x Treatment x Age		F (2, 28) = 0.1318		P=0.8771		
**Tukey's multiple comparisons test**				**P value**		
M:DMSO/CTL 4 wk vs. M:BLZ/PLX 4 wk				>0.9999		
M:DMSO/CTL 4 wk vs. M:DMSO/CTL 7 wk				>0.9999		
M:DMSO/CTL 4 wk vs. M:BLZ/PLX 7 wk				0.9985		
M:DMSO/CTL 4 wk vs. C:DMSO/CTL 4 wk				0.1136		
M:DMSO/CTL 4 wk vs. C:BLZ/PLX 4 wk				0.2258		
M:DMSO/CTL 4 wk vs. C:DMSO/CTL 7 wk				0.0074		
M:DMSO/CTL 4 wk vs. C:BLZ/PLX 7 wk				0.0126		
M:DMSO/CTL 4 wk vs. L:DMSO/CTL 4 wk				>0.9999		
M:DMSO/CTL 4 wk vs. L:BLZ/PLX 4 wk				0.9934		
M:DMSO/CTL 4 wk vs. L:DMSO/CTL 7 wk				>0.9999		
M:DMSO/CTL 4 wk vs. L:BLZ/PLX 7 wk				0.9977		

M:BLZ/PLX 4 wk vs. M:DMSO/CTL 7 wk		>0.9999		
M:BLZ/PLX 4 wk vs. M:BLZ/PLX 7 wk		0.9993		
M:BLZ/PLX 4 wk vs. C:DMSO/CTL 4 wk		0.5308		
M:BLZ/PLX 4 wk vs. C:BLZ/PLX 4 wk		0.5575		
M:BLZ/PLX 4 wk vs. C:DMSO/CTL 7 wk		0.2644		
M:BLZ/PLX 4 wk vs. C:BLZ/PLX 7 wk		0.3047		
M:BLZ/PLX 4 wk vs. L:DMSO/CTL 4 wk		>0.9999		
M:BLZ/PLX 4 wk vs. L:BLZ/PLX 4 wk		>0.9999		
M:BLZ/PLX 4 wk vs. L:DMSO/CTL 7 wk		>0.9999		
M:BLZ/PLX 4 wk vs. L:BLZ/PLX 7 wk		0.9991		
M:DMSO/CTL 7 wk vs. M:BLZ/PLX 7 wk		0.9989		
M:DMSO/CTL 7 wk vs. C:DMSO/CTL 4 wk		0.0671		
M:DMSO/CTL 7 wk vs. C:BLZ/PLX 4 wk		0.1893		
M:DMSO/CTL 7 wk vs. C:DMSO/CTL 7 wk		0.0255		
M:DMSO/CTL 7 wk vs. C:BLZ/PLX 7 wk		0.0048		
M:DMSO/CTL 7 wk vs. L:DMSO/CTL 4 wk		0.9997		
M:DMSO/CTL 7 wk vs. L:BLZ/PLX 4 wk		0.9817		
M:DMSO/CTL 7 wk vs. L:DMSO/CTL 7 wk		>0.9999		
M:DMSO/CTL 7 wk vs. L:BLZ/PLX 7 wk		0.9982		
M:BLZ/PLX 7 wk vs. C:DMSO/CTL 4 wk		0.973		
M:BLZ/PLX 7 wk vs. C:BLZ/PLX 4 wk		0.9988		
M:BLZ/PLX 7 wk vs. C:DMSO/CTL 7 wk		0.7327		
M:BLZ/PLX 7 wk vs. C:BLZ/PLX 7 wk		0.7933		
M:BLZ/PLX 7 wk vs. L:DMSO/CTL 4 wk		0.9882		
M:BLZ/PLX 7 wk vs. L:BLZ/PLX 4 wk		0.9621		
M:BLZ/PLX 7 wk vs. L:DMSO/CTL 7 wk		0.9989		
M:BLZ/PLX 7 wk vs. L:BLZ/PLX 7 wk		>0.9999		
C:DMSO/CTL 4 wk vs. C:BLZ/PLX 4 wk		0.9956		
C:DMSO/CTL 4 wk vs. C:DMSO/CTL 7 wk		0.7783		
C:DMSO/CTL 4 wk vs. C:BLZ/PLX 7 wk		0.8931		
C:DMSO/CTL 4 wk vs. L:DMSO/CTL 4 wk		0.0509		
C:DMSO/CTL 4 wk vs. L:BLZ/PLX 4 wk		0.1175		
C:DMSO/CTL 4 wk vs. L:DMSO/CTL 7 wk		0.1158		
C:DMSO/CTL 4 wk vs. L:BLZ/PLX 7 wk		0.9623		
C:BLZ/PLX 4 wk vs. C:DMSO/CTL 7 wk		0.2881		
C:BLZ/PLX 4 wk vs. C:BLZ/PLX 7 wk		0.3757		
C:BLZ/PLX 4 wk vs. L:DMSO/CTL 4 wk		0.1413		
C:BLZ/PLX 4 wk vs. L:BLZ/PLX 4 wk		0.4269		
C:BLZ/PLX 4 wk vs. L:DMSO/CTL 7 wk		0.2854		
C:BLZ/PLX 4 wk vs. L:BLZ/PLX 7 wk		0.9981		
C:DMSO/CTL 7 wk vs. C:BLZ/PLX 7 wk		0.9999		
C:DMSO/CTL 7 wk vs. L:DMSO/CTL 4 wk		0.0065		
C:DMSO/CTL 7 wk vs. L:BLZ/PLX 4 wk		0.0465		
C:DMSO/CTL 7 wk vs. L:DMSO/CTL 7 wk		0.0863		
C:DMSO/CTL 7 wk vs. L:BLZ/PLX 7 wk		0.6812		
C:BLZ/PLX 7 wk vs. L:DMSO/CTL 4 wk		0.0113		
C:BLZ/PLX 7 wk vs. L:BLZ/PLX 4 wk		0.0638		

C:BLZ/PLX 7 wk vs. L:DMSO/CTL 7 wk				0.0232		
C:BLZ/PLX 7 wk vs. L:BLZ/PLX 7 wk				0.7156		
L:DMSO/CTL 4 wk vs. L:BLZ/PLX 4 wk				>0.9999		
L:DMSO/CTL 4 wk vs. L:DMSO/CTL 7 wk				>0.9999		
L:DMSO/CTL 4 wk vs. L:BLZ/PLX 7 wk				0.9832		
L:BLZ/PLX 4 wk vs. L:DMSO/CTL 7 wk				0.9947		
L:BLZ/PLX 4 wk vs. L:BLZ/PLX 7 wk				0.9504		
L:DMSO/CTL 7 wk vs. L:BLZ/PLX 7 wk				0.9983		
**VGAT (MNTB)**						
**Overall**	**DMSO/CTL**			**BLZ/PLX**		
	**Mean**	**SEM**	**N**	**Mean**	**SEM**	**N**
4 wk	0.247894	0.03803	5	0.25614	0.022431077	6
7 wk	0.28458475	0.03046	4	0.19376	0.039656001	6
**2 way ANOVA**			**Šídák's multiple comparisons test**			**P value**
	**F (DFn, DFd)**	**P value**	4 wk:DMSO/CTL vs. 4 wk:BLZ/PLX			0.998
Interaction	F (1, 17) = 2.081	P=0.1673	4 wk:DMSO/CTL vs. 7 wk:DMSO/CTL			0.8937
Age	F (1, 17) = 0.1399	P=0.7130	4 wk:BLZ/PLX vs. 7 wk:BLZ/PLX			0.5206
Treatment	F (1, 17) = 1.446	P=0.2457	7 wk:DMSO/CTL vs. 7 wk:BLZ/PLX			0.3012
**Regional**	**DMSO/CTL 4 wk**			**BLZ/PLX 4 wk**		
	**Mean**	**SEM**	**N**	**Mean**	**SEM**	**N**
M	0.052676	0.01652	5	0.0763	0.013225031	7
C	0.0576728	0.01508	5	0.15699	0.032476983	7
L	0.1614152	0.04035	5	0.2983	0.026829489	7
	**DMSO/CTL 7 wk**			**BLZ/PLX 7 wk**		
M	0.222429	0.04469	6	0.17301	0.040170441	6
C	0.3202455	0.02359	6	0.26902	0.053534579	6
L	0.215121833	0.02405	6	0.12647	0.031384306	6
**3 way ANOVA**						
		**F (DFn, DFd)**		**P value**		
Region of MNTB		F (1.788, 35.77) = 14.42		P<0.0001		
Treatment		F (1, 20) = 9.750		P=0.0054		
Age		F (1, 20) = 0.1774		P=0.6781		
Region of MNTB x Treatment		F (2, 40) = 37.48		P<0.0001		
Region of MNTB x Age		F (2, 40) = 1.019		P=0.3701		
Treatment x Age		F (1, 20) = 7.192		P=0.0143		
Region of MNTB x Treatment x Age		F (2, 40) = 3.250		P=0.0492		
**Tukey's multiple comparisons test**				**P value**		
M:DMSO/CTL 4 wk vs. M:BLZ/PLX 4 wk				0.9842		
M:DMSO/CTL 4 wk vs. M:DMSO/CTL 7 wk				0.1594		
M:DMSO/CTL 4 wk vs. M:BLZ/PLX 7 wk				0.3513		
M:DMSO/CTL 4 wk vs. C:DMSO/CTL 4 wk				>0.9999		
M:DMSO/CTL 4 wk vs. C:BLZ/PLX 4 wk				0.2926		
M:DMSO/CTL 4 wk vs. C:DMSO/CTL 7 wk				0.0003		
M:DMSO/CTL 4 wk vs. C:BLZ/PLX 7 wk				0.1247		
M:DMSO/CTL 4 wk vs. L:DMSO/CTL 4 wk				0.6482		
M:DMSO/CTL 4 wk vs. L:BLZ/PLX 4 wk				0.0007		

M:DMSO/CTL 4 wk vs. L:DMSO/CTL 7 wk		0.0109		
M:DMSO/CTL 4 wk vs. L:BLZ/PLX 7 wk		0.6472		
M:BLZ/PLX 4 wk vs. M:DMSO/CTL 7 wk		0.2562		
M:BLZ/PLX 4 wk vs. M:BLZ/PLX 7 wk		0.5539		
M:BLZ/PLX 4 wk vs. C:DMSO/CTL 4 wk		0.9962		
M:BLZ/PLX 4 wk vs. C:BLZ/PLX 4 wk		0.165		
M:BLZ/PLX 4 wk vs. C:DMSO/CTL 7 wk		0.0005		
M:BLZ/PLX 4 wk vs. C:BLZ/PLX 7 wk		0.1863		
M:BLZ/PLX 4 wk vs. L:DMSO/CTL 4 wk		0.6873		
M:BLZ/PLX 4 wk vs. L:BLZ/PLX 4 wk		0.0006		
M:BLZ/PLX 4 wk vs. L:DMSO/CTL 7 wk		0.023		
M:BLZ/PLX 4 wk vs. L:BLZ/PLX 7 wk		0.9087		
M:DMSO/CTL 7 wk vs. M:BLZ/PLX 7 wk		0.9987		
M:DMSO/CTL 7 wk vs. C:DMSO/CTL 4 wk		0.1756		
M:DMSO/CTL 7 wk vs. C:BLZ/PLX 4 wk		0.9779		
M:DMSO/CTL 7 wk vs. C:DMSO/CTL 7 wk		0.2014		
M:DMSO/CTL 7 wk vs. C:BLZ/PLX 7 wk		0.9998		
M:DMSO/CTL 7 wk vs. L:DMSO/CTL 4 wk		0.9925		
M:DMSO/CTL 7 wk vs. L:BLZ/PLX 4 wk		0.9196		
M:DMSO/CTL 7 wk vs. L:DMSO/CTL 7 wk		>0.9999		
M:DMSO/CTL 7 wk vs. L:BLZ/PLX 7 wk		0.8072		
M:BLZ/PLX 7 wk vs. C:DMSO/CTL 4 wk		0.3846		
M:BLZ/PLX 7 wk vs. C:BLZ/PLX 4 wk		>0.9999		
M:BLZ/PLX 7 wk vs. C:DMSO/CTL 7 wk		0.2121		
M:BLZ/PLX 7 wk vs. C:BLZ/PLX 7 wk		0.2643		
M:BLZ/PLX 7 wk vs. L:DMSO/CTL 4 wk		>0.9999		
M:BLZ/PLX 7 wk vs. L:BLZ/PLX 4 wk		0.393		
M:BLZ/PLX 7 wk vs. L:DMSO/CTL 7 wk		0.9968		
M:BLZ/PLX 7 wk vs. L:BLZ/PLX 7 wk		0.8166		
C:DMSO/CTL 4 wk vs. C:BLZ/PLX 4 wk		0.3281		
C:DMSO/CTL 4 wk vs. C:DMSO/CTL 7 wk		0.0003		
C:DMSO/CTL 4 wk vs. C:BLZ/PLX 7 wk		0.1357		
C:DMSO/CTL 4 wk vs. L:DMSO/CTL 4 wk		0.166		
C:DMSO/CTL 4 wk vs. L:BLZ/PLX 4 wk		0.0008		
C:DMSO/CTL 4 wk vs. L:DMSO/CTL 7 wk		0.0124		
C:DMSO/CTL 4 wk vs. L:BLZ/PLX 7 wk		0.6994		
C:BLZ/PLX 4 wk vs. C:DMSO/CTL 7 wk		0.051		
C:BLZ/PLX 4 wk vs. C:BLZ/PLX 7 wk		0.7912		
C:BLZ/PLX 4 wk vs. L:DMSO/CTL 4 wk		>0.9999		
C:BLZ/PLX 4 wk vs. L:BLZ/PLX 4 wk		0.0013		
C:BLZ/PLX 4 wk vs. L:DMSO/CTL 7 wk		0.93		
C:BLZ/PLX 4 wk vs. L:BLZ/PLX 7 wk		0.9998		
C:DMSO/CTL 7 wk vs. C:BLZ/PLX 7 wk		0.9969		
C:DMSO/CTL 7 wk vs. L:DMSO/CTL 4 wk		0.1836		
C:DMSO/CTL 7 wk vs. L:BLZ/PLX 4 wk		>0.9999		
C:DMSO/CTL 7 wk vs. L:DMSO/CTL 7 wk		0.3056		
C:DMSO/CTL 7 wk vs. L:BLZ/PLX 7 wk		0.0189		

C:BLZ/PLX 7 wk vs. L:DMSO/CTL 4 wk				0.8704		
C:BLZ/PLX 7 wk vs. L:BLZ/PLX 4 wk				>0.9999		
C:BLZ/PLX 7 wk vs. L:DMSO/CTL 7 wk				0.9956		
C:BLZ/PLX 7 wk vs. L:BLZ/PLX 7 wk				0.0883		
L:DMSO/CTL 4 wk vs. L:BLZ/PLX 4 wk				0.3214		
L:DMSO/CTL 4 wk vs. L:DMSO/CTL 7 wk				0.9784		
L:DMSO/CTL 4 wk vs. L:BLZ/PLX 7 wk				0.9997		
L:BLZ/PLX 4 wk vs. L:DMSO/CTL 7 wk				0.5193		
L:BLZ/PLX 4 wk vs. L:BLZ/PLX 7 wk				0.0452		
L:DMSO/CTL 7 wk vs. L:BLZ/PLX 7 wk				0.5596		
**VGAT (LSO)**						
**Overall**	**DMSO/CTL**			**BLZ/PLX**		
	**Mean**	**SEM**	**N**	**Mean**	**SEM**	**N**
4 wk	0.375861455	0.03636	5	0.37375	0.02329008	6
7 wk	0.380786531	0.02531	6	0.36069	0.032602909	4
**2 way ANOVA**			**Šídák's multiple comparisons test**			**P value**
	**F (DFn, DFd)**	**P value**	4 wk:DMSO/CTL vs. 4 wk:BLZ/PLX			>0.9999
Interaction	F (1, 17) = 0.09399	P=0.7629	4 wk:DMSO/CTL vs. 7 wk:DMSO/CTL			>0.9999
Age	F (1, 17) = 0.01923	P=0.8913	4 wk:BLZ/PLX vs. 7 wk:BLZ/PLX			0.9998
Treatment	F (1, 17) = 0.1433	P=0.7097	7 wk:DMSO/CTL vs. 7 wk:BLZ/PLX			0.998
**Regional**	**DMSO/CTL 4 wk**			**BLZ/PLX 4 wk**		
	**Mean**	**SEM**	**N**	**Mean**	**SEM**	**N**
M	0.3004002	0.0298	5	0.23917	0.047222067	6
C	0.5288312	0.05986	5	0.47651	0.027874887	6
L	0.2676338	0.02206	5	0.38382	0.035630029	6
	**DMSO/CTL 7 wk**			**BLZ/PLX 7 wk**		
M	0.387432167	0.03216	6	0.30103	0.046597398	4
C	0.472106833	0.04427	6	0.45189	0.061130385	4
L	0.261481833	0.02212	6	0.31386	0.03860088	4
**3 way ANOVA**						
		**F (DFn, DFd)**		**P value**		
Region of MNTB		F (1.700, 28.90) = 33.49		P<0.0001		
Treatment		F (1, 17) = 0.002560		P=0.9602		
Age		F (1, 17) = 0.09273		P=0.7644		
Region of MNTB x Treatment		F (2, 34) = 3.523		P=0.0407		
Region of MNTB x Age		F (2, 34) = 5.565		P=0.0081		
Treatment x Age		F (1, 17) = 0.1126		P=0.7413		
Region of MNTB x Treatment x Age		F (2, 34) = 0.4748		P=0.6261		
**Tukey's multiple comparisons test**				**P value**		
M:DMSO/CTL 4 wk vs. M:BLZ/PLX 4 wk				0.9857		
M:DMSO/CTL 4 wk vs. M:DMSO/CTL 7 wk				0.6951		
M:DMSO/CTL 4 wk vs. M:BLZ/PLX 7 wk				>0.9999		
M:DMSO/CTL 4 wk vs. C:DMSO/CTL 4 wk				0.0466		
M:DMSO/CTL 4 wk vs. C:BLZ/PLX 4 wk				0.0469		
M:DMSO/CTL 4 wk vs. C:DMSO/CTL 7 wk				0.194		
M:DMSO/CTL 4 wk vs. C:BLZ/PLX 7 wk				0.5937		

M:DMSO/CTL 4 wk vs. L:DMSO/CTL 4 wk		0.9042		
M:DMSO/CTL 4 wk vs. L:BLZ/PLX 4 wk		0.7892		
M:DMSO/CTL 4 wk vs. L:DMSO/CTL 7 wk		0.9892		
M:DMSO/CTL 4 wk vs. L:BLZ/PLX 7 wk		>0.9999		
M:BLZ/PLX 4 wk vs. M:DMSO/CTL 7 wk		0.3937		
M:BLZ/PLX 4 wk vs. M:BLZ/PLX 7 wk		0.9954		
M:BLZ/PLX 4 wk vs. C:DMSO/CTL 4 wk		0.0986		
M:BLZ/PLX 4 wk vs. C:BLZ/PLX 4 wk		0.007		
M:BLZ/PLX 4 wk vs. C:DMSO/CTL 7 wk		0.1048		
M:BLZ/PLX 4 wk vs. C:BLZ/PLX 7 wk		0.3617		
M:BLZ/PLX 4 wk vs. L:DMSO/CTL 4 wk		>0.9999		
M:BLZ/PLX 4 wk vs. L:BLZ/PLX 4 wk		0.6777		
M:BLZ/PLX 4 wk vs. L:DMSO/CTL 7 wk		>0.9999		
M:BLZ/PLX 4 wk vs. L:BLZ/PLX 7 wk		0.9699		
M:DMSO/CTL 7 wk vs. M:BLZ/PLX 7 wk		0.8869		
M:DMSO/CTL 7 wk vs. C:DMSO/CTL 4 wk		0.6494		
M:DMSO/CTL 7 wk vs. C:BLZ/PLX 4 wk		0.6376		
M:DMSO/CTL 7 wk vs. C:DMSO/CTL 7 wk		0.5101		
M:DMSO/CTL 7 wk vs. C:BLZ/PLX 7 wk		0.9927		
M:DMSO/CTL 7 wk vs. L:DMSO/CTL 4 wk		0.2303		
M:DMSO/CTL 7 wk vs. L:BLZ/PLX 4 wk		>0.9999		
M:DMSO/CTL 7 wk vs. L:DMSO/CTL 7 wk		0.3266		
M:DMSO/CTL 7 wk vs. L:BLZ/PLX 7 wk		0.911		
M:BLZ/PLX 7 wk vs. C:DMSO/CTL 4 wk		0.272		
M:BLZ/PLX 7 wk vs. C:BLZ/PLX 4 wk		0.2511		
M:BLZ/PLX 7 wk vs. C:DMSO/CTL 7 wk		0.3819		
M:BLZ/PLX 7 wk vs. C:BLZ/PLX 7 wk		0.5798		
M:BLZ/PLX 7 wk vs. L:DMSO/CTL 4 wk		0.9995		
M:BLZ/PLX 7 wk vs. L:BLZ/PLX 4 wk		0.9244		
M:BLZ/PLX 7 wk vs. L:DMSO/CTL 7 wk		0.9982		
M:BLZ/PLX 7 wk vs. L:BLZ/PLX 7 wk		>0.9999		
C:DMSO/CTL 4 wk vs. C:BLZ/PLX 4 wk		0.9983		
C:DMSO/CTL 4 wk vs. C:DMSO/CTL 7 wk		0.9991		
C:DMSO/CTL 4 wk vs. C:BLZ/PLX 7 wk		0.9962		
C:DMSO/CTL 4 wk vs. L:DMSO/CTL 4 wk		0.0533		
C:DMSO/CTL 4 wk vs. L:BLZ/PLX 4 wk		0.6481		
C:DMSO/CTL 4 wk vs. L:DMSO/CTL 7 wk		0.1087		
C:DMSO/CTL 4 wk vs. L:BLZ/PLX 7 wk		0.2747		
C:BLZ/PLX 4 wk vs. C:DMSO/CTL 7 wk		>0.9999		
C:BLZ/PLX 4 wk vs. C:BLZ/PLX 7 wk		>0.9999		
C:BLZ/PLX 4 wk vs. L:DMSO/CTL 4 wk		0.0067		
C:BLZ/PLX 4 wk vs. L:BLZ/PLX 4 wk		0.713		
C:BLZ/PLX 4 wk vs. L:DMSO/CTL 7 wk		0.0044		
C:BLZ/PLX 4 wk vs. L:BLZ/PLX 7 wk		0.1926		
C:DMSO/CTL 7 wk vs. C:BLZ/PLX 7 wk		>0.9999		
C:DMSO/CTL 7 wk vs. L:DMSO/CTL 4 wk		0.0751		
C:DMSO/CTL 7 wk vs. L:BLZ/PLX 4 wk		0.8916		

C:DMSO/CTL 7 wk vs. L:DMSO/CTL 7 wk				0.0456		
C:DMSO/CTL 7 wk vs. L:BLZ/PLX 7 wk				0.3623		
C:BLZ/PLX 7 wk vs. L:DMSO/CTL 4 wk				0.3935		
C:BLZ/PLX 7 wk vs. L:BLZ/PLX 4 wk				0.9916		
C:BLZ/PLX 7 wk vs. L:DMSO/CTL 7 wk				0.3671		
C:BLZ/PLX 7 wk vs. L:BLZ/PLX 7 wk				0.5799		
L:DMSO/CTL 4 wk vs. L:BLZ/PLX 4 wk				0.3308		
L:DMSO/CTL 4 wk vs. L:DMSO/CTL 7 wk				>0.9999		
L:DMSO/CTL 4 wk vs. L:BLZ/PLX 7 wk				0.9854		
L:BLZ/PLX 4 wk vs. L:DMSO/CTL 7 wk				0.2779		
L:BLZ/PLX 4 wk vs. L:BLZ/PLX 7 wk				0.9477		
L:DMSO/CTL 7 wk vs. L:BLZ/PLX 7 wk				0.9688		
**GLYT2 (MNTB)**						
**Overall**	**DMSO/CTL**			**BLZ/PLX**		
	**Mean**	**SEM**	**N**	**Mean**	**SEM**	**N**
4 wk	0.097930115	0.01912	5	0.19276	0.024506533	7
7 wk	0.003634134	0.00061	7	0.00415	0.000825382	10
**2 way ANOVA**			**Šídák's multiple comparisons test**			**P value**
	**F (DFn, DFd)**	**P value**	4 wk:DMSO/CTL vs. 4 wk:BLZ/PLX			0.0009
Interactior	F (1, 25) = 11.64	P=0.0022	4 wk:DMSO/CTL vs. 7 wk:DMSO/CTL			0.0009
Age	F (1, 25) = 104.8	P<0.0001	4 wk:BLZ/PLX vs. 7 wk:BLZ/PLX			<0.0001
Treatmen	F (1, 25) = 11.90	P=0.0020	7 wk:DMSO/CTL vs. 7 wk:BLZ/PLX			>0.9999
**Regional**	**DMSO/CTL 4 wk**			**BLZ/PLX 4 wk**		
	**Mean**	**SEM**	**N**	**Mean**	**SEM**	**N**
M	0.052676	0.01652	5	0.0763	0.013225031	7
C	0.0576728	0.01508	5	0.15699	0.032476983	7
L	0.1614152	0.04035	5	0.2983	0.026829489	7
	**DMSO/CTL 7 wk**			**BLZ/PLX 7 wk**		
M	0.001859177	0.00029	6	0.00307	0.001025463	10
C	0.004860531	0.00111	6	0.00541	0.001295316	10
L	0.003858671	0.0021	6	0.00358	0.00113839	10
**3 way ANOVA**						
		**F (DFn, DFd)**		**P value**		
Region of MNTB		F (1.390, 33.35) = 54.36		P<0.0001		
Treatment		F (1, 24) = 97.56		P<0.0001		
Age		F (1, 24) = 10.93		P=0.0030		
Region of MNTB x Treatment		F (2, 48) = 53.63		P<0.0001		
Region of MNTB x Age		F (2, 48) = 5.947		P=0.0049		
Treatment x Age		F (1, 24) = 10.68		P=0.0033		
Region of MNTB x Treatment x		F (2, 48) = 6.251		P=0.0039		
**Tukey's multiple comparisons test**				**P value**		
M:DMSO/CTL 4 wk vs. M:BLZ/PLX 4 wk				0.9842		
M:DMSO/CTL 4 wk vs. M:DMSO/CTL 7 wk				0.3228		
M:DMSO/CTL 4 wk vs. M:BLZ/PLX 7 wk				0.3415		
M:DMSO/CTL 4 wk vs. C:DMSO/CTL 4 wk				>0.9999		
M:DMSO/CTL 4 wk vs. C:BLZ/PLX 4 wk				0.2926		
M:DMSO/CTL 4 wk vs. C:DMSO/CTL 7 wk				0.3707		

M:DMSO/CTL 4 wk vs. C:BLZ/PLX 7 wk		0.3803		
M:DMSO/CTL 4 wk vs. L:DMSO/CTL 4 wk		0.6482		
M:DMSO/CTL 4 wk vs. L:BLZ/PLX 4 wk		0.0007		
M:DMSO/CTL 4 wk vs. L:DMSO/CTL 7 wk		0.3558		
M:DMSO/CTL 4 wk vs. L:BLZ/PLX 7 wk		0.3497		
M:BLZ/PLX 4 wk vs. M:DMSO/CTL 7 wk		0.0239		
M:BLZ/PLX 4 wk vs. M:BLZ/PLX 7 wk		0.0255		
M:BLZ/PLX 4 wk vs. C:DMSO/CTL 4 wk		0.9962		
M:BLZ/PLX 4 wk vs. C:BLZ/PLX 4 wk		0.165		
M:BLZ/PLX 4 wk vs. C:DMSO/CTL 7 wk		0.0287		
M:BLZ/PLX 4 wk vs. C:BLZ/PLX 7 wk		0.0296		
M:BLZ/PLX 4 wk vs. L:DMSO/CTL 4 wk		0.6873		
M:BLZ/PLX 4 wk vs. L:BLZ/PLX 4 wk		0.0006		
M:BLZ/PLX 4 wk vs. L:DMSO/CTL 7 wk		0.026		
M:BLZ/PLX 4 wk vs. L:BLZ/PLX 7 wk		0.0263		
M:DMSO/CTL 7 wk vs. M:BLZ/PLX 7 wk		0.9845		
M:DMSO/CTL 7 wk vs. C:DMSO/CTL 4 wk		0.2021		
M:DMSO/CTL 7 wk vs. C:BLZ/PLX 4 wk		0.0511		
M:DMSO/CTL 7 wk vs. C:DMSO/CTL 7 wk		0.2576		
M:DMSO/CTL 7 wk vs. C:BLZ/PLX 7 wk		0.3521		
M:DMSO/CTL 7 wk vs. L:DMSO/CTL 4 wk		0.1679		
M:DMSO/CTL 7 wk vs. L:BLZ/PLX 4 wk		0.0007		
M:DMSO/CTL 7 wk vs. L:DMSO/CTL 7 wk		0.9958		
M:DMSO/CTL 7 wk vs. L:BLZ/PLX 7 wk		0.9218		
M:BLZ/PLX 7 wk vs. C:DMSO/CTL 4 wk		0.2143		
M:BLZ/PLX 7 wk vs. C:BLZ/PLX 4 wk		0.0529		
M:BLZ/PLX 7 wk vs. C:DMSO/CTL 7 wk		0.9804		
M:BLZ/PLX 7 wk vs. C:BLZ/PLX 7 wk		0.8088		
M:BLZ/PLX 7 wk vs. L:DMSO/CTL 4 wk		0.1715		
M:BLZ/PLX 7 wk vs. L:BLZ/PLX 4 wk		0.0007		
M:BLZ/PLX 7 wk vs. L:DMSO/CTL 7 wk		>0.9999		
M:BLZ/PLX 7 wk vs. L:BLZ/PLX 7 wk		>0.9999		
C:DMSO/CTL 4 wk vs. C:BLZ/PLX 4 wk		0.3281		
C:DMSO/CTL 4 wk vs. C:DMSO/CTL 7 wk		0.2343		
C:DMSO/CTL 4 wk vs. C:BLZ/PLX 7 wk		0.2407		
C:DMSO/CTL 4 wk vs. L:DMSO/CTL 4 wk		0.166		
C:DMSO/CTL 4 wk vs. L:BLZ/PLX 4 wk		0.0008		
C:DMSO/CTL 4 wk vs. L:DMSO/CTL 7 wk		0.2228		
C:DMSO/CTL 4 wk vs. L:BLZ/PLX 7 wk		0.2198		
C:BLZ/PLX 4 wk vs. C:DMSO/CTL 7 wk		0.0557		
C:BLZ/PLX 4 wk vs. C:BLZ/PLX 7 wk		0.0565		
C:BLZ/PLX 4 wk vs. L:DMSO/CTL 4 wk		>0.9999		
C:BLZ/PLX 4 wk vs. L:BLZ/PLX 4 wk		0.0013		
C:BLZ/PLX 4 wk vs. L:DMSO/CTL 7 wk		0.0539		
C:BLZ/PLX 4 wk vs. L:BLZ/PLX 7 wk		0.0536		
C:DMSO/CTL 7 wk vs. C:BLZ/PLX 7 wk		>0.9999		
C:DMSO/CTL 7 wk vs. L:DMSO/CTL 4 wk		0.1771		

C:DMSO/CTL 7 wk vs. L:BLZ/PLX 4 wk				0.0007		
C:DMSO/CTL 7 wk vs. L:DMSO/CTL 7 wk				>0.9999		
C:DMSO/CTL 7 wk vs. L:BLZ/PLX 7 wk				0.9991		
C:BLZ/PLX 7 wk vs. L:DMSO/CTL 4 wk				0.1789		
C:BLZ/PLX 7 wk vs. L:BLZ/PLX 4 wk				0.0007		
C:BLZ/PLX 7 wk vs. L:DMSO/CTL 7 wk				0.9999		
C:BLZ/PLX 7 wk vs. L:BLZ/PLX 7 wk				0.9457		
L:DMSO/CTL 4 wk vs. L:BLZ/PLX 4 wk				0.3214		
L:DMSO/CTL 4 wk vs. L:DMSO/CTL 7 wk				0.1738		
L:DMSO/CTL 4 wk vs. L:BLZ/PLX 7 wk				0.1731		
L:BLZ/PLX 4 wk vs. L:DMSO/CTL 7 wk				0.0007		
L:BLZ/PLX 4 wk vs. L:BLZ/PLX 7 wk				0.0007		
L:DMSO/CTL 7 wk vs. L:BLZ/PLX 7 wk				>0.9999		
**GLYT2 (LSO)**						
**Overall**	**DMSO/CTL**			**BLZ/PLX**		
	**Mean**	**SEM**	**N**	**Mean**	**SEM**	**N**
4 wk	0.097930115	0.01912	5	0.19276	0.024506533	7
7 wk	0.003634134	0.00061	7	0.00415	0.000825382	10
**2 way ANOVA**			**Šídák's multiple comparisons test**			**P value**
	**F (DFn, DFd)**	**P value**	4 wk:DMSO/CTL vs. 4 wk:BLZ/PLX			0.7053
Interactior	F (1, 18) = 5.880	P=0.0261	4 wk:DMSO/CTL vs. 7 wk:DMSO/CTL			<0.0001
Age	F (1, 18) = 46.29	P<0.0001	4 wk:BLZ/PLX vs. 7 wk:BLZ/PLX			0.029
Treatmen	F (1, 18) = 0.9365	P=0.3460	7 wk:DMSO/CTL vs. 7 wk:BLZ/PLX			0.1359
**Regional**	**DMSO/CTL 4 wk**			**BLZ/PLX 4 wk**		
	**Mean**	**SEM**	**N**	**Mean**	**SEM**	**N**
M	0.2770915	0.02437	6	0.26007	0.014300296	6
C	0.423117333	0.00883	6	0.43693	0.012065177	6
L	0.270821667	0.02023	6	0.32506	0.016254995	6
	**DMSO/CTL 7 wk**			**BLZ/PLX 7 wk**		
M	0.4121888	0.03316	5	0.3786	0.038702228	5
C	0.5681412	0.02937	5	0.53468	0.012587024	5
L	0.3129106	0.02974	5	0.26638	0.053898057	5
**3 way ANOVA**						
		**F (DFn, DFd)**		**P value**		
Region of MNTB		F (1.344, 24.20) = 54.52		P<0.0001		
Treatment		F (1, 18) = 44.93		P<0.0001		
Age		F (1, 18) = 0.7636		P=0.3937		
Region of MNTB x Treatment		F (2, 36) = 7.312		P=0.0022		
Region of MNTB x Age		F (2, 36) = 0.2660		P=0.7680		
Treatment x Age		F (1, 18) = 5.288		P=0.0337		
Region of MNTB x Treatment x		F (2, 36) = 0.5674		P=0.5720		
**Tukey's multiple comparisons test**				**P value**		
M:DMSO/CTL 4wk vs. M:BLZ/PLX 4 wk				>0.9999		
M:DMSO/CTL 4wk vs. M:DMSO/CTL 7 wk				0.189		
M:DMSO/CTL 4wk vs. M:BLZ/PLX 7 wk				0.5803		
M:DMSO/CTL 4wk vs. C:DMSO/CTL 4wk				0.0301		
M:DMSO/CTL 4wk vs. C:BLZ/PLX 4 wk				0.0116		

M:DMSO/CTL 4wk vs. C:DMSO/CTL 7 wk		0.0014		
M:DMSO/CTL 4wk vs. C:BLZ/PLX 7 wk		0.0006		
M:DMSO/CTL 4wk vs. L:DMSO/CTL 4wk		>0.9999		
M:DMSO/CTL 4wk vs. L:BLZ/PLX 4 wk		0.8576		
M:DMSO/CTL 4wk vs. L:DMSO/CTL 7 wk		0.9957		
M:DMSO/CTL 4wk vs. L:BLZ/PLX 7 wk		>0.9999		
M:BLZ/PLX 4 wk vs. M:DMSO/CTL 7 wk		0.0978		
M:BLZ/PLX 4 wk vs. M:BLZ/PLX 7 wk		0.3464		
M:BLZ/PLX 4 wk vs. C:DMSO/CTL 4wk		0.0002		
M:BLZ/PLX 4 wk vs. C:BLZ/PLX 4 wk		0.0084		
M:BLZ/PLX 4 wk vs. C:DMSO/CTL 7 wk		0.0018		
M:BLZ/PLX 4 wk vs. C:BLZ/PLX 7 wk		<0.0001		
M:BLZ/PLX 4 wk vs. L:DMSO/CTL 4wk		>0.9999		
M:BLZ/PLX 4 wk vs. L:BLZ/PLX 4 wk		0.6034		
M:BLZ/PLX 4 wk vs. L:DMSO/CTL 7 wk		0.8606		
M:BLZ/PLX 4 wk vs. L:BLZ/PLX 7 wk		>0.9999		
M:DMSO/CTL 7 wk vs. M:BLZ/PLX 7 wk		0.9998		
M:DMSO/CTL 7 wk vs. C:DMSO/CTL 4wk		>0.9999		
M:DMSO/CTL 7 wk vs. C:BLZ/PLX 4 wk		0.9993		
M:DMSO/CTL 7 wk vs. C:DMSO/CTL 7 wk		0.3382		
M:DMSO/CTL 7 wk vs. C:BLZ/PLX 7 wk		0.2062		
M:DMSO/CTL 7 wk vs. L:DMSO/CTL 4wk		0.1388		
M:DMSO/CTL 7 wk vs. L:BLZ/PLX 4 wk		0.5234		
M:DMSO/CTL 7 wk vs. L:DMSO/CTL 7 wk		0.7043		
M:DMSO/CTL 7 wk vs. L:BLZ/PLX 7 wk		0.5419		
M:BLZ/PLX 7 wk vs. C:DMSO/CTL 4wk		0.9747		
M:BLZ/PLX 7 wk vs. C:BLZ/PLX 4 wk		0.9087		
M:BLZ/PLX 7 wk vs. C:DMSO/CTL 7 wk		0.0944		
M:BLZ/PLX 7 wk vs. C:BLZ/PLX 7 wk		0.0987		
M:BLZ/PLX 7 wk vs. L:DMSO/CTL 4wk		0.4744		
M:BLZ/PLX 7 wk vs. L:BLZ/PLX 4 wk		0.953		
M:BLZ/PLX 7 wk vs. L:DMSO/CTL 7 wk		0.9456		
M:BLZ/PLX 7 wk vs. L:BLZ/PLX 7 wk		0.9332		
C:DMSO/CTL 4wk vs. C:BLZ/PLX 4 wk		0.9964		
C:DMSO/CTL 4wk vs. C:DMSO/CTL 7 wk		0.0775		
C:DMSO/CTL 4wk vs. C:BLZ/PLX 7 wk		0.003		
C:DMSO/CTL 4wk vs. L:DMSO/CTL 4wk		0.0062		
C:DMSO/CTL 4wk vs. L:BLZ/PLX 4 wk		0.0184		
C:DMSO/CTL 4wk vs. L:DMSO/CTL 7 wk		0.201		
C:DMSO/CTL 4wk vs. L:BLZ/PLX 7 wk		0.3702		
C:BLZ/PLX 4 wk vs. C:DMSO/CTL 7 wk		0.1082		
C:BLZ/PLX 4 wk vs. C:BLZ/PLX 7 wk		0.0094		
C:BLZ/PLX 4 wk vs. L:DMSO/CTL 4wk		0.0026		
C:BLZ/PLX 4 wk vs. L:BLZ/PLX 4 wk		0.0068		
C:BLZ/PLX 4 wk vs. L:DMSO/CTL 7 wk		0.1385		
C:BLZ/PLX 4 wk vs. L:BLZ/PLX 7 wk		0.3063		
C:DMSO/CTL 7 wk vs. C:BLZ/PLX 7 wk		0.9859		

C:DMSO/CTL 7 wk vs. L:DMSO/CTL 4wk		0.0013		
C:DMSO/CTL 7 wk vs. L:BLZ/PLX 4 wk		0.0055		
C:DMSO/CTL 7 wk vs. L:DMSO/CTL 7 wk		0.0084		
C:DMSO/CTL 7 wk vs. L:BLZ/PLX 7 wk		0.0426		
C:BLZ/PLX 7 wk vs. L:DMSO/CTL 4wk		0.0001		
C:BLZ/PLX 7 wk vs. L:BLZ/PLX 4 wk		0.0001		
C:BLZ/PLX 7 wk vs. L:DMSO/CTL 7 wk		0.0123		
C:BLZ/PLX 7 wk vs. L:BLZ/PLX 7 wk		0.1015		
L:DMSO/CTL 4wk vs. L:BLZ/PLX 4 wk		0.6397		
L:DMSO/CTL 4wk vs. L:DMSO/CTL 7 wk		0.9764		
L:DMSO/CTL 4wk vs. L:BLZ/PLX 7 wk		>0.9999		
L:BLZ/PLX 4 wk vs. L:DMSO/CTL 7 wk		>0.9999		
L:BLZ/PLX 4 wk vs. L:BLZ/PLX 7 wk		0.9847		
L:DMSO/CTL 7 wk vs. L:BLZ/PLX 7 wk		0.999		
