# Supplementary material for: Long-term microglia depletion impairs synapse elimination and auditory brainstem function
Source: Sci Rep. 2022 Nov 2;12:18521. doi: 10.1038/s41598-022-23250-5 (PMC9630367; doi:10.1038/s41598-022-23250-5)
Supplement: Supplementary file 5 — Supplementary Table S4. [file 41598_2022_23250_MOESM5_ESM.pdf]

Supplementary Table 4-Auditory Brainstem Response Descriptive Statistics

Two-way ANOVA

			**Clicks threshold**		**Pure tones**		
	**Stimulus (kHz)**	**Source of Variation**	**F (DFn, DFd)**	**P value**	**Source of Variation**	**F (DFn, DFd)**	**P value**
	**Clicks**	Interaction	F (1, 63) = 2.488	P=0.1197	Frequency	F (3.029, 191.6) = 66.90	<0.0001
		Age	F (1, 63) = 7.731	P=0.0072	Treatment	F (3, 64) = 31.42	<0.0001
		Treatment	F (1, 63) = 154.1	P<0.0001	Frequency x Treatment	F (12, 253) = 7.560	<0.0001

Šídák's multiple comparisons test

Clicks

	**Comparison**	**t**	**df**	**P value**
	4 wk:DMSO/CTL vs. 4 wk:BLZ/PLX	7.178	63	<0.0001
	4 wk:DMSO/CTL vs. 7 wk:DMSO/CTL	0.8266	63	0.9585
	4 wk:BLZ/PLX vs. 7 wk:BLZ/PLX	3.177	63	0.0138
	7 wk:DMSO/CTL vs. 7 wk:BLZ/PLX	10.67	63	<0.0001

Pure tones

**Stimulus (kHz)**	**Comparison**	Mean 1	Mean 2	P value
**8**	DMSO/CTL 4wk vs. BLZ/PLX 4wk	21.92	42.19	<0.0001
	DMSO/CTL 4wk vs. DMSO/CTL 7wk	21.92	21.58	0.9996
	BLZ/PLX 4wk vs. BLZ/PLX 7wk	42.19	51.05	0.0241
	DMSO/CTL 7wk vs. BLZ/PLX 7wk	21.58	51.05	<0.0001
**12**	DMSO/CTL 4wk vs. BLZ/PLX 4wk	23.08	34.38	0.0113

	DMSO/CTL 4wk vs. DMSO/CTL 7wk	23.08	19	0.6587
	BLZ/PLX 4wk vs.	34.38	48.16	<0.0001
	DMSO/CTL 7wk vs. BLZ/PLX 7wk	19	48.16	<0.0001
**16**	DMSO/CTL 4wk vs. BLZ/PLX 4wk	26.92	34.06	0.1328
	DMSO/CTL 4wk vs. DMSO/CTL 7wk	26.92	24.25	0.8696
	BLZ/PLX 4wk vs. BLZ/PLX 7wk	34.06	45.79	0.0002
	DMSO/CTL 7wk vs. BLZ/PLX 7wk	24.25	45.79	<0.0001
**24**	DMSO/CTL 4wk vs. BLZ/PLX 4wk	38.75	44.69	0.6475
	DMSO/CTL 4wk vs. DMSO/CTL 7wk	38.75	33.25	0.7033
	BLZ/PLX 4wk vs. BLZ/PLX 7wk	44.69	49.74	0.343
	DMSO/CTL 7wk vs. BLZ/PLX 7wk	33.25	49.74	<0.0001
**32**	DMSO/CTL 4wk vs. BLZ/PLX 4wk	44.62	49.67	0.6278
	DMSO/CTL 4wk vs. DMSO/CTL 7wk	44.62	39.75	0.6039
	BLZ/PLX 4wk vs. BLZ/PLX 7wk	49.67	52.11	0.8819
	DMSO/CTL 7wk vs. BLZ/PLX 7wk	39.75	52.11	0.001

Two-way ANOVA with Šídák's multiple comparisons test

			**4 wk**							**7 wk**							
			**Amplitude (µV)**		**Latency (ms)**		**Interpeak latency (ms)**				**Amplitude (µV)**		**Latency (ms)**		**Interpeak latency (ms)**		
**Peak**	**Stimulus (kHz)**	**Source of Variation**	**F (DFn, DFd)**	**P value**	**F (DFn, DFd)**	**P value**	**Peak diff.**	**F (DFn, DFd)**	**P value**	**Peak**	**F (DFn, DFd)**	**P value**	**F (DFn, DFd)**	**P value**	**Peak diff.**	**F (DFn, DFd)**	**P value**
**I**	**8**	Interaction	F (10, 191) = 0.1272	P=0.9994	F (8, 177) = 0.04536	P>0.9999	I-II	F (8, 175) = 0.6437	P=0.7401	I	F (7, 237) = 0.09467	P=0.9986	F (7, 237) = 2.645	P=0.0119	I-II	F (7, 237) = 0.5255	P=0.8149
		Intensity (dB SPL)	F (10, 191) = 29.97	P<0.0001	F (8, 177) = 15.76	P<0.0001		F (8, 175) = 0.4009	P=0.9190		F (7, 237) = 26.24	P<0.0001	F (7, 237) = 24.01	P<0.0001		F (7, 237) = 0.5201	P=0.8190
		Treatment	F (1, 191) = 1.089	P=0.2981	F (1, 177) = 16.91	P<0.0001		F (1, 175) = 0.4263	P=0.5147		F (1, 237) = 65.86	P<0.0001	F (1, 237) = 182.9	P<0.0001		F (1, 237) = 0.2276	P=0.6338
	**12**	Interaction	F (11, 208) = 0.3802	P=0.9627	F (10, 203) = 0.3474	P=0.9667		F (10, 201) = 0.1361	P=0.9993		F (9, 287) = 0.8603	P=0.5613	F (9, 287) = 0.5145	P=0.8637		F (9, 287) = 0.7639	P=0.6500

		Intensity (dB SPL)	F (11, 208) = 36.48	P<0.0001	F (10, 203) = 13.18	P<0.0001		F (10, 201) = 0.4717	P=0.9069		F (9, 287) = 26.60	P<0.0001	F (9, 287) = 16.50	P<0.0001		F (9, 287) = 0.8127	P=0.6049
		Treatment	F (1, 208) = 0.9840	P=0.3224	F (1, 203) = 2.512	P=0.1146		F (1, 201) = 0.03732	P=0.8470		F (1, 287) = 90.69	P<0.0001	F (1, 287) = 106.5	P<0.0001		F (1, 287) = 0.2480	P=0.6189
	**16**	Interaction	F (11, 198) = 0.1733	P=0.9987	F (11, 197) = 0.4770	P=0.9160		F (10, 192) = 0.4182	P=0.9367		F (8, 267) = 0.6487	P=0.7363	F (8, 267) = 0.3484	P=0.9461		F (8, 267) = 0.9272	P=0.4944
		Intensity (dB SPL)	F (11, 198) = 31.64	P<0.0001	F (11, 197) = 7.346	P<0.0001		F (10, 192) = 0.7919	P=0.6366		F (8, 267) = 24.91	P<0.0001	F (8, 267) = 15.04	P<0.0001		F (8, 267) = 1.266	P=0.2614
		Treatment	F (1, 198) = 0.9235	P=0.3377	F (1, 197) = 1.505	P=0.2213		F (1, 192) = 2.612	P=0.1077		F (1, 267) = 98.40	P<0.0001	F (1, 267) = 77.53	P<0.0001		F (1, 267) = 0.005492	P=0.9410
	**24**	Interaction	F (7, 138) = 0.2827	P=0.9598	F (8, 144) = 0.1365	P=0.9975		F (8, 144) = 0.6241	P=0.7565		F (7, 228) = 0.4074	P=0.8972	F (7, 228) = 0.4811	P=0.8478		F (7, 228) = 0.7985	P=0.5893
		Intensity (dB SPL)	F (7, 138) = 27.20	P<0.0001	F (8, 144) = 2.601	P=0.0110		F (8, 144) = 0.2775	P=0.9724		F (7, 228) = 22.50	P<0.0001	F (7, 228) = 6.374	P<0.0001		F (7, 228) = 0.5958	P=0.7590
		Treatment	F (1, 138) = 0.3322	P=0.5653	F (1, 144) = 5.603	P=0.0193		F (1, 144) = 6.415	P=0.0124		F (1, 228) = 42.21	P<0.0001	F (1, 228) = 3.257	P=0.0724		F (1, 228) = 6.838	P=0.0095
	**32**	Interaction	F (7, 116) = 0.1361	P=0.9953	F (7, 126) = 0.4233	P=0.8863		F (7, 126) = 0.4274	P=0.8836		F (7, 215) = 0.2803	P=0.9612	F (8, 281) = 0.1872	P=0.9925		F (7, 214) = 0.6855	P=0.6843
		Intensity (dB SPL)	F (7, 116) = 19.57	P<0.0001	F (7, 126) = 0.2289	P=0.9777		F (7, 126) = 0.1620	P=0.9920		F (7, 215) = 16.34	P<0.0001	F (8, 281) = 3.717	P=0.0004		F (7, 214) = 0.2853	P=0.9592
		Treatment	F (1, 116) = 0.2403	P=0.6249	F (1, 126) = 3.327	P=0.0705		F (1, 126) = 1.043	P=0.3090		F (1, 215) = 32.67	P<0.0001	F (1, 281) = 0.008054	P=0.9286		F (1, 214) = 0.9352	P=0.3346
	**8**	Interaction	F (8, 175) = 0.8378	P=0.5706	F (8, 175) = 0.4172	P=0.9096	II-III	F (8, 175) = 0.8190	P=0.5868	II	F (7, 237) = 0.3159	P=0.9464	F (7, 237) = 0.7820	P=0.6030	II-III	F (7, 234) = 2.093	P=0.0451
		Intensity (dB SPL)	F (8, 175) = 14.54	P<0.0001	F (8, 175) = 2.310	P=0.0222		F (8, 175) = 0.5696	P=0.8018		F (7, 237) = 13.27	P<0.0001	F (7, 237) = 3.582	P=0.0011		F (7, 234) = 1.095	P=0.3669
		Treatment	F (1, 175) = 1.316	P=0.2529	F (1, 175) = 5.934	P=0.0159		F (1, 175) = 0.7511	P=0.3873		F (1, 237) = 68.17	P<0.0001	F (1, 237) = 36.42	P<0.0001		F (1, 234) = 49.90	P<0.0001
	**12**	Interaction	F (10, 201) = 0.9122	P=0.5229	F (10, 201) = 0.05545	P>0.9999		F (10, 198) = 0.5937	P=0.8180		F (9, 287) = 0.7412	P=0.6710	F (9, 287) = 0.2912	P=0.9769		F (8, 265) = 0.3944	P=0.9231
		Intensity (dB SPL)	F (10, 201) = 16.60	P<0.0001	F (10, 201) = 2.593	P=0.0056		F (10, 198) = 0.9606	P=0.4792		F (9, 287) = 22.83	P<0.0001	F (9, 287) = 2.802	P=0.0036		F (8, 265) = 0.1449	P=0.9969

		Treatment	F (1, 201) = 0.2381	P=0.6261	F (1, 201) = 0.3366	P=0.5624		F (1, 198) = 0.1223	P=0.7270		F (1, 287) = 113.5	P<0.0001	F (1, 287) = 28.50	P<0.0001		F (1, 265) = 88.47	P<0.0001
	**16**	Interaction	F (10, 192) = 0.4110	P=0.9403	F (10, 192) = 0.4663	P=0.9102		F (10, 192) = 0.5790	P=0.8300		F (8, 267) = 1.292	P=0.2475	F (8, 267) = 0.2810	P=0.9718		F (8, 264) = 0.5338	P=0.8306
**II**		Intensity (dB SPL)	F (10, 192) = 10.60	P<0.0001	F (10, 192) = 1.002	P=0.4430		F (10, 192) = 1.995	P=0.0358		F (8, 267) = 16.82	P<0.0001	F (8, 267) = 1.686	P=0.1019		F (8, 264) = 0.4580	P=0.8848
		Treatment	F (1, 192) = 0.06233	P=0.8031	F (1, 192) = 2.414	P=0.1219		F (1, 192) = 0.006439	P=0.9361		F (1, 267) = 118.3	P<0.0001	F (1, 267) = 17.66	P<0.0001		F (1, 264) = 76.05	P<0.0001
	**24**	Interaction	F (8, 144) = 1.917	P=0.0616	F (8, 144) = 0.3964	P=0.9211		F (8, 142) = 0.5303	P=0.8322		F (7, 227) = 2.682	P=0.0110	F (7, 228) = 0.9326	P=0.4820		F (7, 218) = 0.4657	P=0.8587
		Intensity (dB SPL)	F (8, 144) = 0.8553	P=0.5559	F (8, 144) = 0.8466	P=0.5633		F (8, 142) = 0.8765	P=0.5380		F (7, 227) = 6.559	P<0.0001	F (7, 228) = 2.256	P=0.0308		F (7, 218) = 0.7021	P=0.6703
		Treatment	F (1, 144) = 0.6634	P=0.4167	F (1, 144) = 8.821	P=0.0035		F (1, 142) = 1.233	P=0.2687		F (1, 227) = 22.80	P<0.0001	F (1, 228) = 1.171	P=0.2804		F (1, 218) = 56.66	P<0.0001
	**32**	Interaction	F (7, 126) = 0.8033	P=0.5861	F (7, 126) = 0.5121	P=0.8240		F (7, 125) = 0.5439	P=0.7997		F (7, 214) = 2.464	P=0.0189	F (7, 214) = 0.6546	P=0.7102		F (7, 211) = 1.017	P=0.4198
		Intensity (dB SPL)	F (7, 126) = 0.9464	P=0.4735	F (7, 126) = 0.2334	P=0.9764		F (7, 125) = 1.776	P=0.0978		F (7, 214) = 0.7842	P=0.6012	F (7, 214) = 1.341	P=0.2322		F (7, 211) = 0.4370	P=0.8782
		Treatment	F (1, 126) = 0.1011	P=0.7510	F (1, 126) = 2.403	P=0.1236		F (1, 125) = 0.3162	P=0.5749		F (1, 214) = 1.689	P=0.1952	F (1, 214) = 0.6138	P=0.4342		F (1, 211) = 29.23	P<0.0001
	**8**	Interaction	F (8, 175) = 0.3392	P=0.9497	F (8, 175) = 0.1130	P=0.9987	III-IV	F (10, 197) = 0.3890	P=0.9505	III	F (7, 234) = 0.2409	P=0.9746	F (7, 234) = 0.4518	P=0.8684	III-IV	F (7, 234) = 1.674	P=0.1161
		Intensity (dB SPL)	F (8, 175) = 11.97	P<0.0001	F (8, 175) = 1.413	P=0.1940		F (10, 197) = 1.522	P=0.1338		F (7, 234) = 16.33	P<0.0001	F (7, 234) = 1.040	P=0.4040		F (7, 234) = 0.6995	P=0.6725
		Treatment	F (1, 175) = 19.14	P<0.0001	F (1, 175) = 1.607	P=0.2066		F (1, 197) = 8.883	P=0.0032		F (1, 234) = 54.63	P<0.0001	F (1, 234) = 93.73	P<0.0001		F (1, 234) = 30.01	P<0.0001
	**12**	Interaction	F (10, 198) = 0.5038	P=0.8861	F (10, 198) = 0.1857	P=0.9971		F (10, 190) = 0.3446	P=0.9676		F (8, 265) = 1.062	P=0.3903	F (8, 265) = 0.2262	P=0.9859		F (8, 261) = 0.2933	P=0.9678
		Intensity (dB SPL)	F (10, 198) = 12.77	P<0.0001	F (10, 198) = 1.988	P=0.0364		F (10, 190) = 0.6165	P=0.7987		F (8, 265) = 17.94	P<0.0001	F (8, 265) = 1.369	P=0.2101		F (8, 261) = 2.529	P=0.0115

		Treatment	F (1, 198) = 12.44	P=0.0005	F (1, 198) = 0.8994	P=0.3441		F (1, 190) = 9.304	P=0.0026		F (1, 265) = 86.22	P<0.0001	F (1, 265) = 95.13	P<0.0001		F (1, 261) = 44.15	P<0.0001
	**16**	Interaction	F (10, 192) = 0.3025	P=0.9799	F (10, 192) = 0.05787	P>0.9999		F (8, 144) = 0.9519	P=0.4761		F (8, 264) = 1.231	P=0.2807	F (8, 264) = 0.1418	P=0.9972		F (8, 261) = 1.495	P=0.1590
**III**		Intensity (dB SPL)	F (10, 192) = 12.15	P<0.0001	F (10, 192) = 1.963	P=0.0393		F (8, 144) = 2.923	P=0.0047		F (8, 264) = 26.78	P<0.0001	F (8, 264) = 1.250	P=0.2701		F (8, 261) = 0.9967	P=0.4389
		Treatment	F (1, 192) = 4.562	P=0.0340	F (1, 192) = 1.230	P=0.2689		F (1, 144) = 8.104	P=0.0051		F (1, 264) = 116.7	P<0.0001	F (1, 264) = 69.60	P<0.0001		F (1, 261) = 14.49	P=0.0002
	**24**	Interaction	F (8, 142) = 0.3715	P=0.9342	F (8, 142) = 0.3694	P=0.9352		F (8, 140) = 0.8243	P=0.5825		F (7, 218) = 0.5315	P=0.8102	F (7, 218) = 0.3512	P=0.9292		F (7, 217) = 2.486	P=0.0178
		Intensity (dB SPL)	F (8, 142) = 15.91	P<0.0001	F (8, 142) = 1.845	P=0.0735		F (8, 140) = 1.351	P=0.2233		F (7, 218) = 24.94	P<0.0001	F (7, 218) = 2.643	P=0.0121		F (7, 217) = 0.4154	P=0.8922
		Treatment	F (1, 142) = 1.280	P=0.2598	F (1, 142) = 1.724	P=0.1913		F (1, 140) = 10.87	P=0.0012		F (1, 218) = 35.10	P<0.0001	F (1, 218) = 27.33	P<0.0001		F (1, 217) = 0.5391	P=0.4636
	**32**	Interaction	F (7, 125) = 0.08732	P=0.9989	F (7, 125) = 0.3582	P=0.9247		F (7, 123) = 0.5183	P=0.8194		F (7, 211) = 0.1375	P=0.9953	F (7, 211) = 0.1679	P=0.9912		F (7, 209) = 0.2556	P=0.9699
		Intensity (dB SPL)	F (7, 125) = 4.047	P=0.0005	F (7, 125) = 1.134	P=0.3463		F (7, 123) = 0.8663	P=0.5353		F (7, 211) = 14.44	P<0.0001	F (7, 211) = 0.8773	P=0.5253		F (7, 209) = 0.3713	P=0.9183
		Treatment	F (1, 125) = 1.334	P=0.2503	F (1, 125) = 3.174	P=0.0772		F (1, 123) = 7.642	P=0.0066		F (1, 211) = 27.07	P<0.0001	F (1, 211) = 16.45	P<0.0001		F (1, 209) = 0.1535	P=0.6956
**IV**	**8**	Interaction	F (8, 173) = 0.5191	P=0.8410	F (8, 173) = 0.1479	P=0.9966	I-IV	F (8, 173) = 0.1913	P=0.9918	IV	F (7, 234) = 0.8372	P=0.5574	F (7, 234) = 0.1275	P=0.9963	I-IV	F (7, 234) = 0.6394	P=0.7231
		Intensity (dB SPL)	F (8, 173) = 0.4765	P=0.8717	F (8, 173) = 1.998	P=0.0493		F (8, 173) = 0.5401	P=0.8251		F (7, 234) = 0.9933	P=0.4368	F (7, 234) = 0.9529	P=0.4666		F (7, 234) = 0.3782	P=0.9145
		Treatment	F (1, 173) = 19.07	P<0.0001	F (1, 173) = 4.442	P=0.0365		F (1, 173) = 1.790	P=0.1827		F (1, 234) = 30.24	P<0.0001	F (1, 234) = 112.1	P<0.0001		F (1, 234) = 72.08	P<0.0001
	**12**	Interaction	F (10, 197) = 0.7965	P=0.6322	F (10, 197) = 0.1068	P=0.9997		F (10, 197) = 0.1016	P=0.9998		F (8, 260) = 4.322	P<0.0001	F (8, 261) = 0.1330	P=0.9977		F (8, 261) = 0.2061	P=0.9897
		Intensity (dB SPL)	F (10, 197) = 1.726	P=0.0770	F (10, 197) = 2.406	P=0.0102		F (10, 197) = 0.9022	P=0.5322		F (8, 260) = 0.7160	P=0.6774	F (8, 261) = 2.197	P=0.0280		F (8, 261) = 0.4241	P=0.9061
		Treatment	F (1, 197) = 25.14	P<0.0001	F (1, 197) = 5.551	P=0.0195		F (1, 197) = 4.572	P=0.0337		F (1, 260) = 35.07	P<0.0001	F (1, 261) = 111.8	P<0.0001		F (1, 261) = 97.45	P<0.0001

	**16**	Interaction	F (10, 190) = 1.198	P=0.2944	F (10, 190) = 0.1324	P=0.9993		F (10, 190) = 0.1502	P=0.9988		F (8, 258) = 2.151	P=0.0317	F (8, 261) = 0.2829	P=0.9712		F (8, 261) = 0.6107	P=0.7686
		Intensity (dB SPL)	F (10, 190) = 4.350	P<0.0001	F (10, 190) = 1.412	P=0.1775		F (10, 190) = 0.5452	P=0.8563		F (8, 258) = 2.190	P=0.0287	F (8, 261) = 1.244	P=0.2739		F (8, 261) = 0.1829	P=0.9931
		Treatment	F (1, 190) = 40.31	P<0.0001	F (1, 190) = 0.2581	P=0.6120		F (1, 190) = 0.4476	P=0.5043		F (1, 258) = 108.2	P<0.0001	F (1, 261) = 65.80	P<0.0001		F (1, 261) = 53.10	P<0.0001
	**24**	Interaction	F (8, 140) = 0.4778	P=0.8703	F (8, 140) = 0.2563	P=0.9785		F (8, 140) = 0.3717	P=0.9340		F (7, 217) = 1.805	P=0.0875	F (7, 217) = 0.2505	P=0.9716		F (7, 217) = 0.5223	P=0.8172
		Intensity (dB SPL)	F (8, 140) = 1.872	P=0.0690	F (8, 140) = 1.626	P=0.1225		F (8, 140) = 1.288	P=0.2544		F (7, 217) = 2.133	P=0.0414	F (7, 217) = 2.006	P=0.0556		F (7, 217) = 0.3514	P=0.9290
		Treatment	F (1, 140) = 17.39	P<0.0001	F (1, 140) = 8.537	P=0.0041		F (1, 140) = 7.835	P=0.0058		F (1, 217) = 39.26	P<0.0001	F (1, 217) = 22.23	P<0.0001		F (1, 217) = 28.14	P<0.0001
	**32**	Interaction	F (7, 123) = 0.1962	P=0.9858	F (7, 123) = 0.3503	P=0.9288		F (7, 123) = 0.3072	P=0.9495		F (7, 209) = 1.313	P=0.2454	F (7, 209) = 0.1191	P=0.9970		F (7, 209) = 0.2367	P=0.9758
		Intensity (dB SPL)	F (7, 123) = 2.192	P=0.0394	F (7, 123) = 0.9190	P=0.4943		F (7, 123) = 1.064	P=0.3909		F (7, 209) = 3.984	P=0.0004	F (7, 209) = 1.457	P=0.1844		F (7, 209) = 0.9820	P=0.4453
		Treatment	F (1, 123) = 5.666	P=0.0188	F (1, 123) = 7.484	P=0.0071		F (1, 123) = 7.401	P=0.0075		F (1, 209) = 9.329	P=0.0025	F (1, 209) = 10.10	P=0.0017		F (1, 209) = 9.814	P=0.0020
**Comparisons**		**Amplitude (μV)**															
			**DMSO/CTL 4 wk**		**BLZ/PLX 4 wk**			**DMSO/CTL 7 wk**		**BLZ/PLX 7 wk**							
**Peak**	**Stimulus (kHz)**	**Intensity (dB SPL)**	**Mean**	**SEM**	**Mean**	**SEM**	**P value**	**Mean**	**SEM**	**Mean**	**SEM**	**P value**					
**I**	**8**	10															
		15															
		20															
		25															
		30	1.08	0.14	0.79	0.17	>0.9999										
		35	1.19	0.17	0.79	0.26	1										
		40	0.98	0.17	1.00	0.15	>0.9999										
		45	1.34	0.24	1.17	0.16	>0.9999	1.06	0.12	0.34	0.05	0.5069					
		50	1.62	0.26	1.42	0.23	>0.9999	1.34	0.14	0.46	0.06	0.0563					

		55	2.15	0.26	2.00	0.20	>0.9999	1.63	0.16	0.68	0.08	0.0137					
		60	2.79	0.31	2.39	0.24	0.993	2.00	0.20	1.01	0.11	0.008					
		65	2.96	0.28	2.87	0.28	>0.9999	2.39	0.24	1.31	0.14	0.0028					
		70	3.56	0.39	3.43	0.31	>0.9999	2.63	0.25	1.73	0.15	0.0224					
		75	4.26	0.51	4.08	0.35	>0.9999	3.12	0.27	2.17	0.19	0.0135					
		80	4.62	0.54	4.83	0.41	>0.9999	3.41	0.29	2.56	0.21	0.0377					
	**12**	10															
		15															
		20															
		25	0.74	0.17	0.50	0.12	>0.9999										
		30	1.30	0.17	0.68	0.11	0.99										
		35	0.97	0.20	0.85	0.11	>0.9999	0.84	0.10	0.37	0.14	0.9949					
		40	1.15	0.19	0.95	0.09	>0.9999	1.02	0.11	0.33	0.05	0.5546					
		45	1.20	0.22	1.15	0.15	>0.9999	1.26	0.14	0.45	0.06	0.2223					
		50	1.61	0.25	1.49	0.17	>0.9999	1.58	0.15	0.53	0.06	0.0206					
		55	2.18	0.32	1.72	0.17	0.969	1.85	0.19	0.75	0.08	0.0118					
		60	2.40	0.35	2.20	0.17	>0.9999	2.19	0.21	0.97	0.13	0.0037					
		65	2.96	0.38	2.74	0.21	>0.9999	2.60	0.23	1.21	0.13	0.0003					
		70	3.48	0.41	3.43	0.31	>0.9999	2.99	0.28	1.53	0.16	0.0001					
		75	3.85	0.38	4.22	0.40	0.996	3.45	0.37	2.06	0.20	0.0003					
		80	4.56	0.43	4.87	0.38	0.999	4.17	0.41	2.55	0.21	<0.0001					
	**16**	10															
		15															
		20															
		25	0.81	0.31	0.49	0.10	>0.9999										
		30	1.05	0.18	0.57	0.11	1										
		35	0.98	0.18	0.85	0.09	>0.9999										
		40	1.20	0.21	0.96	0.14	>0.9999	0.89	0.08	0.31	0.05	0.4938					

		45	1.21	0.19	1.15	0.14	>0.9999	1.18	0.10	0.46	0.04	0.1119					
		50	1.52	0.17	1.34	0.17	>0.9999	1.33	0.13	0.60	0.05	0.0975					
		55	1.67	0.17	1.55	0.23	>0.9999	1.65	0.16	0.70	0.09	0.0047					
		60	2.24	0.21	1.98	0.30	1	1.83	0.17	0.99	0.10	0.0197					
		65	2.67	0.30	2.49	0.33	>0.9999	2.15	0.20	1.16	0.11	0.0029					
		70	3.02	0.35	2.97	0.38	>0.9999	2.47	0.23	1.40	0.14	0.0009					
		75	3.54	0.33	3.78	0.44	>0.9999	2.92	0.31	1.68	0.14	<0.0001					
		80	4.48	0.37	4.65	0.55	>0.9999	3.32	0.29	2.10	0.19	<0.0001					
	**24**	10															
		15															
		20															
		25															
		30															
		35															
		40															
		45	0.66	0.30	0.61	0.11	>0.9999	0.72	0.08	0.32	0.07	0.6423					
		50	0.81	0.15	0.89	0.12	>0.9999	0.83	0.09	0.40	0.03	0.4827					
		55	0.97	0.17	1.02	0.14	>0.9999	0.90	0.11	0.51	0.05	0.491					
		60	1.27	0.19	1.14	0.16	1	1.15	0.15	0.70	0.05	0.3162					
		65	1.75	0.21	1.36	0.16	0.758	1.50	0.18	0.87	0.09	0.0375					
		70	1.99	0.23	1.91	0.21	>0.9999	1.69	0.16	1.09	0.08	0.0493					
		75	2.32	0.20	2.38	0.21	>0.9999	1.88	0.20	1.39	0.12	0.1895					
		80	2.94	0.30	2.91	0.27	>0.9999	2.47	0.26	1.67	0.12	0.0022					
	**32**	10															
		15															
		20															
		25															
		30															
		35															

		40															
		45	0.48	0.04	0.61	0.19	>0.9999	0.55	0.07	0.24	0.05	0.938					
		50	0.67	0.16	0.80	0.27	>0.9999	0.74	0.11	0.44	0.07	0.8368					
		55	0.97	0.16	0.90	0.16	>0.9999	0.91	0.12	0.39	0.04	0.1135					
		60	1.07	0.20	1.16	0.15	>0.9999	0.97	0.11	0.63	0.08	0.5465					
		65	1.25	0.17	1.35	0.13	>0.9999	1.20	0.13	0.77	0.09	0.1973					
		70	1.43	0.19	1.58	0.15	0.998	1.45	0.15	0.99	0.09	0.1253					
		75	1.96	0.28	1.86	0.15	>0.9999	1.64	0.15	1.13	0.12	0.0556					
		80	2.46	0.28	2.43	0.16	>0.9999	1.97	0.19	1.35	0.12	0.0093					
II	**8**	10															
		15															
		20															
		25															
		30															
		35															
		40	1.59	0.40	1.44	0.24	>0.9999										
		45	1.73	0.38	1.88	0.19	>0.9999	2.15	0.16	0.78	0.10	0.4872					
		50	1.94	0.43	2.13	0.22	>0.9999	2.39	0.22	0.93	0.11	0.1218					
		55	2.68	0.51	2.41	0.26	>0.9999	2.76	0.31	1.09	0.10	0.0229					
		60	3.03	0.50	2.92	0.28	>0.9999	3.18	0.36	1.41	0.11	0.0129					
		65	3.41	0.46	3.59	0.33	>0.9999	3.93	0.40	1.73	0.14	0.0008					
		70	3.89	0.50	4.19	0.34	>0.9999	4.30	0.43	2.20	0.19	0.0016					
		75	4.49	0.65	4.78	0.53	>0.9999	4.77	0.54	2.81	0.34	0.0041					
		80	4.38	0.66	6.13	0.59	0.062	5.19	0.62	3.85	0.53	0.1254					
	**12**	10															
		15															
		20															
		25															

		30	1.78	0.36	1.20	0.15	1										
		35	1.73	0.36	1.44	0.18	>0.9999	2.12	0.13	0.53	0.04	0.5343					
		40	2.18	0.50	1.66	0.14	0.999	2.45	0.17	0.78	0.08	0.0572					
		45	2.10	0.48	1.73	0.19	>0.9999	2.46	0.18	0.96	0.10	0.0687					
		50	2.24	0.44	2.12	0.22	>0.9999	2.75	0.26	1.13	0.08	0.0198					
		55	2.69	0.51	2.36	0.23	>0.9999	3.08	0.32	1.40	0.09	0.0138					
		60	2.74	0.48	2.78	0.28	>0.9999	3.61	0.40	1.47	0.09	0.0005					
		65	3.13	0.51	3.62	0.41	0.997	4.25	0.48	1.61	0.13	<0.0001					
		70	3.83	0.49	4.54	0.45	0.947	4.86	0.52	2.23	0.24	<0.0001					
		75	4.37	0.56	5.12	0.44	0.934	5.61	0.51	3.29	0.37	<0.0001					
		80	4.67	0.63	5.96	0.57	0.312	6.09	0.48	4.54	0.30	0.0259					
	**16**	10															
		15															
		20															
		25															
		30	2.02	0.42	1.22	0.15	0.997										
		35	1.53	0.39	1.38	0.12	>0.9999										
		40	1.78	0.44	1.83	0.19	>0.9999	2.07	0.12	0.79	0.08	0.2455					
		45	2.04	0.49	1.77	0.20	>0.9999	2.36	0.19	0.96	0.04	0.0771					
		50	2.21	0.44	2.10	0.23	>0.9999	2.60	0.16	1.27	0.06	0.1084					
		55	2.32	0.34	2.17	0.27	>0.9999	2.86	0.24	1.40	0.11	0.0343					
		60	2.85	0.45	2.68	0.31	>0.9999	3.17	0.29	1.55	0.09	0.0118					
		65	3.13	0.59	3.53	0.39	1	3.81	0.41	1.77	0.17	0.0006					
		70	3.34	0.55	3.83	0.46	0.997	4.58	0.55	2.16	0.22	<0.0001					
		75	3.81	0.57	4.32	0.55	0.996	5.34	0.54	2.52	0.30	<0.0001					
		80	4.23	0.58	4.98	0.62	0.929	5.80	0.55	3.28	0.30	<0.0001					
	**24**	10															
		15															
		20															
		25															

		30															
		35															
		40	1.72	0.08	0.99	0.17	0.944										
		45	1.78	0.33	1.37	0.15	0.997	1.33	0.14	0.67	0.05	0.3499					
		50	1.68	0.24	1.54	0.19	>0.9999	1.33	0.12	1.02	0.05	0.9539					
		55	1.83	0.28	1.66	0.22	>0.9999	1.42	0.11	1.28	0.09	0.9997					
		60	1.83	0.29	1.83	0.32	>0.9999	1.33	0.11	1.42	0.11	>0.9999					
		65	1.75	0.38	1.78	0.27	>0.9999	1.65	0.17	1.44	0.13	0.9924					
		70	1.76	0.34	2.19	0.26	0.937	1.83	0.22	1.38	0.15	0.6407					
		75	1.64	0.25	2.44	0.29	0.31	2.53	0.31	1.44	0.15	0.0015					
		80	1.38	0.32	2.67	0.34	0.01	2.77	0.37	1.50	0.15	0.0001					
	**32**	10															
		15															
		20															
		25															
		30															
		35															
		40															
		45	1.52	0.28	0.90	0.14	0.949	1.21	0.11	0.59	0.10	0.3187					
		50	1.65	0.38	1.12	0.09	0.981	1.33	0.11	0.96	0.13	0.711					
		55	1.32	0.31	1.29	0.18	>0.9999	1.34	0.11	1.15	0.13	0.9831					
		60	1.46	0.34	1.79	0.25	0.993	1.20	0.13	1.38	0.15	0.9854					
		65	1.35	0.27	1.98	0.18	0.69	1.10	0.12	1.39	0.13	0.7604					
		70	1.56	0.34	2.06	0.26	0.852	1.07	0.12	1.42	0.14	0.5198					
		75	1.91	0.39	1.82	0.25	>0.9999	1.14	0.15	1.14	0.14	>0.9999					
		80	1.45	0.33	1.69	0.31	0.999	1.44	0.22	0.98	0.14	0.1724					
**III**	**8**	10															
		15															
		20															
		25															
		30															
		35															
		40	1.01	0.18	0.84	0.26	>0.9999										

		45	1.40	0.23	0.95	0.25	0.997	1.65	0.15	0.51	0.10	0.4475					
		50	2.02	0.27	1.36	0.28	0.939	2.02	0.18	0.68	0.08	0.0721					
		55	2.70	0.40	1.58	0.28	0.365	2.58	0.24	1.13	0.10	0.0145					
		60	3.31	0.41	1.99	0.27	0.174	3.07	0.29	1.70	0.11	0.0195					
		65	3.52	0.41	2.50	0.37	0.496	3.67	0.33	2.16	0.14	0.0068					
		70	3.90	0.44	2.81	0.43	0.411	3.91	0.39	2.55	0.19	0.0211					
		75	4.35	0.52	3.17	0.52	0.306	4.08	0.45	2.98	0.25	0.1088					
		80	4.23	0.66	3.37	0.51	0.712	4.20	0.47	3.37	0.27	0.4137					
	**12**	10															
		15															
		20															
		25															
		30	0.88	0.17	0.58	0.25	>0.9999										
		35	1.08	0.16	0.87	0.19	>0.9999										
		40	1.48	0.34	1.04	0.19	0.999	1.45	0.15	0.55	0.08	0.4824					
		45	1.75	0.24	1.05	0.18	0.949	1.84	0.20	0.69	0.09	0.1042					
		50	2.07	0.31	1.20	0.24	0.686	2.27	0.26	0.86	0.10	0.0158					
		55	2.45	0.40	1.52	0.27	0.596	2.79	0.28	1.24	0.13	0.0032					
		60	2.98	0.42	1.60	0.31	0.1	3.20	0.30	1.53	0.20	0.0012					
		65	3.30	0.46	2.28	0.40	0.453	3.54	0.31	1.65	0.21	<0.0001					
		70	3.42	0.43	2.84	0.50	0.969	3.76	0.37	1.93	0.25	0.0002					
		75	3.68	0.46	3.34	0.54	1	3.96	0.38	2.71	0.24	0.0279					
		80	3.88	0.46	3.79	0.49	>0.9999	4.06	0.41	3.51	0.28	0.8574					
	**16**	10															
		15															
		20															
		25															
		30	1.55	0.54	0.92	0.26	0.998										
		35	1.14	0.31	0.85	0.19	>0.9999										
		40	1.43	0.29	1.11	0.17	>0.9999	1.34	0.16	0.61	0.10	0.5586					
		45	1.45	0.32	1.32	0.20	>0.9999	1.52	0.14	0.67	0.08	0.1948					
		50	1.69	0.33	1.29	0.28	0.999	1.89	0.19	0.98	0.11	0.1125					
		55	1.82	0.35	1.53	0.28	1	2.28	0.23	1.11	0.07	0.006					

		60	2.43	0.38	1.73	0.26	0.876	2.67	0.26	1.34	0.11	0.0012					
		65	2.88	0.48	2.34	0.31	0.975	3.18	0.30	1.44	0.12	<0.0001					
		70	3.27	0.46	2.68	0.44	0.957	3.72	0.34	1.86	0.15	<0.0001					
		75	3.72	0.42	3.31	0.51	0.997	3.93	0.33	2.31	0.17	<0.0001					
		80	3.41	0.36	3.76	0.53	1	4.41	0.29	3.01	0.23	0.0006					
	**24**	10															
		15															
		20															
		25															
		30															
		35															
		40	1.22	0.06	0.96	0.25	>0.9999										
		45	1.59	0.33	1.22	0.24	0.997	1.43	0.13	0.69	0.09	0.2796					
		50	1.27	0.24	1.54	0.19	0.999	1.66	0.13	0.92	0.08	0.1731					
		55	2.02	0.25	1.69	0.16	0.991	2.08	0.17	1.25	0.09	0.0494					
		60	2.17	0.22	1.79	0.26	0.975	2.18	0.18	1.51	0.15	0.1591					
		65	2.37	0.32	2.47	0.23	>0.9999	2.60	0.24	1.86	0.17	0.0775					
		70	2.97	0.34	2.60	0.22	0.968	2.75	0.21	2.23	0.19	0.4337					
		75	3.31	0.34	3.09	0.27	0.999	3.31	0.23	2.65	0.23	0.1583					
		80	3.49	0.32	3.58	0.32	>0.9999	3.29	0.25	3.13	0.26	0.9986					
	**32**	10															
		15															
		20															
		25															
		30															
		35															
		40															
		45	1.43	0.24	1.08	0.29	1	1.31	0.13	0.53	0.17	0.676					
		50	1.70	0.36	1.48	0.29	>0.9999	1.66	0.14	0.79	0.13	0.1864					
		55	1.70	0.23	1.51	0.13	>0.9999	1.83	0.16	1.28	0.12	0.5159					
		60	2.14	0.40	1.77	0.19	0.993	2.17	0.18	1.50	0.13	0.2022					
		65	2.08	0.31	2.05	0.20	>0.9999	2.48	0.16	1.84	0.18	0.2355					
		70	2.55	0.38	2.13	0.29	0.973	2.74	0.23	2.27	0.22	0.6246					

		75	2.62	0.43	2.53	0.32	>0.9999	2.95	0.22	2.40	0.28	0.3614					
		80	2.94	0.45	2.84	0.36	>0.9999	3.05	0.28	2.51	0.25	0.4166					
**IV**	**8**	10															
		15															
		20															
		25															
		30															
		35															
		40	1.55	0.21	0.91	0.11	0.946										
		45	1.77	0.27	1.29	0.24	0.978	1.68	0.18	0.67	0.05	0.4066					
		50	1.79	0.29	1.14	0.19	0.812	1.84	0.20	0.73	0.08	0.0914					
		55	2.07	0.49	1.10	0.16	0.259	2.04	0.24	0.98	0.08	0.0616					
		60	2.15	0.52	1.18	0.12	0.262	2.14	0.27	1.22	0.10	0.1338					
		65	2.39	0.47	1.14	0.17	0.053	2.16	0.33	1.14	0.06	0.0696					
		70	2.13	0.41	1.45	0.17	0.732	2.12	0.33	1.26	0.12	0.1942					
		75	2.02	0.39	1.52	0.20	0.938	1.96	0.35	1.58	0.17	0.9561					
		80	1.65	0.33	1.53	0.23	>0.9999	1.85	0.37	1.74	0.21	>0.9999					
	**12**	10															
		15															
		20															
		25															
		30	1.70	0.18	0.71	0.18	0.76										
		35	1.48	0.37	1.02	0.10	0.994										
		40	1.50	0.37	0.94	0.08	0.954	1.82	0.17	0.51	0.07	0.0022					
		45	1.66	0.29	0.88	0.10	0.587	1.79	0.20	0.65	0.05	0.0025					
		50	1.76	0.29	1.00	0.13	0.461	1.71	0.19	0.76	0.11	0.0165					
		55	2.04	0.31	1.07	0.11	0.151	1.67	0.20	0.97	0.08	0.1459					
		60	2.33	0.39	1.28	0.17	0.088	1.77	0.22	0.99	0.11	0.0674					
		65	2.34	0.43	1.33	0.17	0.115	1.87	0.25	1.13	0.12	0.0948					
		70	2.18	0.38	1.53	0.21	0.699	1.71	0.23	1.23	0.14	0.5785					
		75	2.04	0.40	1.73	0.23	0.999	1.42	0.23	1.53	0.17	>0.9999					
		80	1.78	0.40	1.92	0.26	>0.9999	1.16	0.20	1.82	0.20	0.1755					
	**16**	10															
		15															

		20															
		25															
		30	1.23	0.25	0.73	0.20	0.997										
		35	1.17	0.29	0.77	0.13	0.993										
		40	0.99	0.20	1.07	0.14	>0.9999	1.69	0.16	0.54	0.06	0.0084					
		45	1.72	0.23	0.99	0.12	0.587	1.90	0.18	0.55	0.05	<0.0001					
		50	1.84	0.20	0.91	0.15	0.207	2.08	0.20	0.82	0.06	<0.0001					
		55	2.28	0.27	0.97	0.16	0.011	1.90	0.18	0.84	0.08	0.0004					
		60	2.31	0.34	1.07	0.12	0.015	2.00	0.22	0.94	0.11	0.0005					
		65	2.51	0.39	1.26	0.15	0.014	2.08	0.19	1.06	0.11	0.0009					
		70	2.69	0.48	1.49	0.19	0.022	2.24	0.22	1.22	0.16	0.001					
		75	2.66	0.48	1.63	0.19	0.083	2.00	0.20	1.37	0.15	0.1452					
		80	2.23	0.47	1.84	0.22	0.983	1.72	0.21	1.67	0.12	>0.9999					
	**24**	10															
		15															
		20															
		25															
		30															
		35															
		40	1.31	0.12	0.91	0.24	1										
		45	1.54	0.20	1.02	0.22	0.994	1.41	0.17	0.47	0.09	0.0207					
		50	1.28	0.14	1.03	0.17	1	1.63	0.17	0.67	0.07	0.0091					
		55	1.49	0.24	0.89	0.09	0.869	1.70	0.16	0.77	0.10	0.0029					
		60	2.07	0.29	1.34	0.28	0.686	1.61	0.19	1.09	0.11	0.2649					
		65	2.46	0.39	1.06	0.13	0.023	1.72	0.21	1.18	0.10	0.2057					
		70	2.04	0.40	1.29	0.23	0.551	1.66	0.17	1.26	0.09	0.5705					
		75	2.36	0.52	1.48	0.25	0.343	1.74	0.21	1.36	0.13	0.6529					
		80	2.49	0.54	1.49	0.34	0.186	1.45	0.22	1.50	0.16	>0.9999					
	**32**	10															
		15															
		20															
		25															
		30															
		35															
		40															
		45	1.63	0.18	0.96	0.33	0.94	1.19	0.09	0.79	0.14	0.9849					

		50	1.71	0.27	1.33	0.34	0.999	1.47	0.08	0.63	0.12	0.1772					
		55	1.65	0.26	1.04	0.20	0.887	1.73	0.11	0.87	0.09	0.0463					
		60	1.73	0.27	1.12	0.17	0.822	1.68	0.18	1.15	0.10	0.438					
		65	1.87	0.32	1.59	0.17	0.997	1.59	0.21	1.48	0.10	>0.9999					
		70	2.10	0.37	1.87	0.21	0.999	1.76	0.22	1.77	0.18	>0.9999					
		75	2.00	0.37	1.91	0.34	>0.9999	1.87	0.25	1.81	0.17	>0.9999					
		80	2.55	0.47	2.02	0.31	0.869	1.90	0.31	1.83	0.15	>0.9999					
		**Latency (ms)**															
			**DMSO/CTL 4 wk**		**BLZ/PLX 4 wk**			**DMSO/CTL 7 wk**		**BLZ/PLX 7 wk**							
**Peak**	**Stimulus (kHz)**	**Intensity (dB SPL)**	**Mean**	**SEM**	**Mean**	**SEM**	**P value**	**Mean**	**SEM**	**Mean**	**SEM**	**P value**					
I	**8**	10															
		15															
		20															
		25															
		30															
		35															
		40	2.07	0.04	2.14	0.09	0.954										
		45	2.04	0.03	2.10	0.07	0.945	1.88	0.02	2.22	0.06	<0.0001					
		50	1.98	0.02	2.04	0.05	0.902	1.84	0.02	2.09	0.06	<0.0001					
		55	1.93	0.02	2.01	0.05	0.702	1.81	0.02	2.05	0.04	<0.0001					
		60	1.87	0.03	1.95	0.05	0.723	1.77	0.02	1.97	0.04	<0.0001					
		65	1.83	0.03	1.91	0.04	0.679	1.73	0.02	1.91	0.03	<0.0001					
		70	1.78	0.03	1.88	0.04	0.459	1.70	0.02	1.87	0.03	0.0002					
		75	1.77	0.03	1.85	0.03	0.744	1.70	0.02	1.82	0.03	0.0229					
		80	1.75	0.03	1.82	0.04	0.92	1.68	0.02	1.79	0.03	0.039					
	**12**	10															
		15															
		20															
		25															
		30	2.09	0.07	2.11	0.13	>0.9999										
		35	2.07	0.05	2.10	0.07	>0.9999	1.92	0.02	2.12	0.03	0.0461					
		40	2.05	0.04	2.06	0.05	>0.9999	1.89	0.02	2.04	0.03	0.0154					

		45	2.02	0.03	2.01	0.04	>0.9999	1.88	0.03	2.06	0.05	0.0007					
		50	1.99	0.04	1.98	0.04	>0.9999	1.83	0.02	1.98	0.04	0.0037					
		55	1.95	0.03	1.94	0.04	>0.9999	1.79	0.02	1.95	0.04	0.001					
		60	1.85	0.03	1.90	0.04	0.989	1.74	0.02	1.90	0.04	0.0016					
		65	1.85	0.03	1.89	0.04	0.999	1.73	0.02	1.88	0.04	0.0023					
		70	1.79	0.04	1.87	0.03	0.764	1.71	0.02	1.85	0.04	0.0076					
		75	1.78	0.04	1.85	0.04	0.947	1.70	0.02	1.82	0.04	0.0261					
		80	1.77	0.03	1.82	0.03	0.994	1.71	0.03	1.79	0.04	0.4326					
	**16**	10															
		15															
		20	2.15	0.10													
		25	2.13	0.07	1.91	0.06	0.588										
		30	2.03	0.05	2.06	0.12	>0.9999										
		35	2.06	0.04	2.09	0.06	>0.9999										
		40	2.00	0.03	2.01	0.07	>0.9999	1.91	0.03	2.07	0.04	0.0276					
		45	2.03	0.05	2.01	0.05	>0.9999	1.88	0.03	2.07	0.06	0.001					
		50	1.99	0.04	1.95	0.05	>0.9999	1.84	0.02	2.00	0.05	0.0112					
		55	1.96	0.04	1.92	0.04	>0.9999	1.82	0.03	1.96	0.04	0.0136					
		60	1.95	0.04	1.89	0.04	0.995	1.79	0.02	1.93	0.04	0.0265					
		65	1.89	0.04	1.86	0.04	>0.9999	1.76	0.02	1.88	0.04	0.0574					
		70	1.86	0.04	1.86	0.03	>0.9999	1.73	0.02	1.83	0.04	0.1749					
		75	1.82	0.03	1.85	0.03	>0.9999	1.71	0.02	1.82	0.04	0.1063					
		80	1.81	0.04	1.82	0.03	>0.9999	1.68	0.02	1.79	0.04	0.1409					
	**24**	10															
		15															
		20															
		25															
		30															
		35															

		40	1.96	0.01	1.90	0.10	0.999										
		45	1.96	0.04	1.86	0.06	0.933	1.90	0.07	2.07	0.13	0.1976					
		50	1.93	0.09	1.85	0.05	0.915	1.83	0.06	1.85	0.03	>0.9999					
		55	1.85	0.04	1.81	0.05	1	1.82	0.05	1.84	0.04	>0.9999					
		60	1.83	0.04	1.78	0.05	0.997	1.77	0.04	1.81	0.05	0.9979					
		65	1.83	0.03	1.82	0.05	>0.9999	1.76	0.03	1.78	0.04	>0.9999					
		70	1.82	0.03	1.76	0.04	0.959	1.73	0.03	1.76	0.04	0.9995					
		75	1.80	0.03	1.74	0.04	0.972	1.71	0.03	1.73	0.04	>0.9999					
		80	1.79	0.03	1.73	0.04	0.982	1.70	0.03	1.72	0.04	>0.9999					
	**32**	10															
		15															
		20															
		25															
		30															
		35															
		40						1.90	0.06	1.87	0.03	>0.9999					
		45	1.92	0.02	1.82	0.11	0.983	1.84	0.05	1.85	0.04	>0.9999					
		50	1.91	0.04	1.79	0.07	0.94	1.81	0.04	1.87	0.07	0.9667					
		55	1.80	0.03	1.83	0.06	>0.9999	1.79	0.04	1.80	0.03	>0.9999					
		60	1.78	0.04	1.80	0.05	>0.9999	1.79	0.04	1.77	0.03	>0.9999					
		65	1.87	0.07	1.79	0.05	0.959	1.76	0.03	1.76	0.03	>0.9999					
		70	1.86	0.07	1.81	0.05	0.997	1.76	0.03	1.74	0.03	>0.9999					
		75	1.90	0.05	1.78	0.04	0.691	1.73	0.03	1.75	0.04	>0.9999					
		80	1.87	0.05	1.81	0.08	0.989	1.71	0.03	1.71	0.03	>0.9999					
**II**	**8**	10															
		15															
		20															
		25															
		30															
		35															
		40	3.15	0.10	3.32	0.16	0.943										
		45	3.13	0.08	3.40	0.13	0.285	2.95	0.06	3.43	0.14	0.0029					
		50	3.08	0.07	3.16	0.07	0.998	2.94	0.06	3.16	0.10	0.2247					
		55	3.17	0.09	3.16	0.07	>0.9999	2.89	0.06	3.07	0.08	0.3802					

		60	3.10	0.09	3.15	0.08	>0.9999	2.86	0.06	3.03	0.08	0.5253					
		65	3.04	0.09	3.08	0.08	>0.9999	2.84	0.04	3.04	0.08	0.2642					
		70	3.00	0.09	3.08	0.06	0.999	2.81	0.05	2.98	0.07	0.422					
		75	2.96	0.10	3.08	0.05	0.956	2.79	0.05	2.98	0.09	0.3329					
		80	2.94	0.10	3.03	0.05	0.99	2.79	0.05	2.92	0.10	0.8248					
	**12**	10															
		15															
		20															
		25															
		30	3.40	0.15	3.42	0.16	>0.9999										
		35	3.28	0.12	3.28	0.09	>0.9999	3.04	0.04	3.23	0.04	0.8787					
		40	3.24	0.12	3.24	0.07	>0.9999	2.99	0.04	3.21	0.07	0.2243					
		45	3.16	0.13	3.23	0.07	>0.9999	2.97	0.06	3.18	0.07	0.2234					
		50	3.16	0.10	3.15	0.06	>0.9999	2.93	0.05	3.07	0.08	0.6279					
		55	3.09	0.09	3.11	0.07	>0.9999	2.91	0.05	2.99	0.09	0.9943					
		60	3.07	0.11	3.11	0.08	>0.9999	2.87	0.05	2.96	0.09	0.9816					
		65	3.02	0.11	3.08	0.07	>0.9999	2.87	0.04	3.05	0.09	0.3047					
		70	3.03	0.11	3.10	0.05	>0.9999	2.84	0.04	3.02	0.09	0.3325					
		75	3.05	0.08	3.05	0.05	>0.9999	2.83	0.04	2.99	0.08	0.4235					
		80	3.01	0.08	3.00	0.05	>0.9999	2.81	0.04	2.98	0.06	0.3927					
	**16**	10															
		15															
		20															
		25															
		30	3.26	0.13	3.30	0.13	>0.9999										
		35	3.20	0.08	3.23	0.09	>0.9999										
		40	3.08	0.12	3.15	0.08	>0.9999	3.00	0.05	3.16	0.09	0.7833					

		45	3.02	0.12	3.06	0.06	>0.9999	2.99	0.05	3.13	0.11	0.7916					
		50	3.17	0.15	3.00	0.06	0.949	2.93	0.06	3.03	0.09	0.9749					
		55	3.24	0.14	2.99	0.07	0.486	2.92	0.06	2.99	0.08	0.9963					
		60	3.18	0.13	3.08	0.08	0.999	2.89	0.06	2.96	0.09	0.9976					
		65	3.14	0.13	3.04	0.07	0.999	2.88	0.05	3.02	0.09	0.7484					
		70	3.09	0.12	2.98	0.07	0.999	2.84	0.05	3.04	0.08	0.3094					
		75	3.09	0.11	2.97	0.07	0.997	2.82	0.05	2.98	0.09	0.5551					
		80	3.04	0.10	2.92	0.06	0.996	2.78	0.05	2.99	0.08	0.2462					
	**24**	10															
		15															
		20															
		25															
		30															
		35															
		40	2.97	0.05	2.82	0.16	0.999										
		45	2.98	0.09	2.82	0.12	0.998	2.86	0.10	3.11	0.21	0.5598					
		50	3.15	0.23	2.76	0.09	0.244	2.79	0.08	2.72	0.05	0.9993					
		55	3.04	0.20	2.72	0.09	0.42	2.75	0.07	2.72	0.10	>0.9999					
		60	2.90	0.13	2.69	0.06	0.884	2.68	0.07	2.66	0.08	>0.9999					
		65	2.89	0.13	2.75	0.06	0.985	2.78	0.09	2.61	0.08	0.7836					
		70	2.82	0.11	2.70	0.06	0.993	2.75	0.08	2.58	0.08	0.7426					
		75	2.81	0.10	2.72	0.08	1	2.75	0.08	2.61	0.08	0.922					
		80	2.75	0.10	2.68	0.07	>0.9999	2.70	0.08	2.67	0.07	>0.9999					
	**32**	10															
		15															
		20															
		25															
		30															
		35															
		40															
		45	3.08	0.27	2.71	0.15	0.864	2.68	0.07	2.73	0.04	>0.9999					
		50	3.06	0.26	2.66	0.15	0.819	2.67	0.08	2.88	0.19	0.6757					
		55	2.70	0.18	2.73	0.10	>0.9999	2.57	0.05	2.66	0.05	0.9856					
		60	2.64	0.19	2.79	0.11	0.996	2.60	0.07	2.68	0.10	0.9947					

		65	2.79	0.21	2.74	0.09	>0.9999	2.60	0.06	2.63	0.08	>0.9999					
		70	2.86	0.13	2.70	0.08	0.988	2.62	0.06	2.56	0.09	0.9994					
		75	2.88	0.12	2.73	0.11	0.991	2.59	0.07	2.55	0.08	0.9999					
		80	2.81	0.11	2.73	0.12	1	2.59	0.09	2.50	0.08	0.9809					
**III**	**8**	10															
		15															
		20															
		25															
		30															
		35															
		40	4.11	0.15	4.13	0.17	>0.9999										
		45	4.20	0.12	4.27	0.13	>0.9999	3.78	0.05	4.19	0.15	0.0649					
		50	4.05	0.12	4.27	0.18	0.861	3.71	0.05	4.08	0.09	0.0305					
		55	4.11	0.12	4.14	0.09	>0.9999	3.64	0.07	4.20	0.14	<0.0001					
		60	4.01	0.11	4.07	0.09	>0.9999	3.61	0.07	4.10	0.11	0.0001					
		65	3.97	0.12	4.03	0.09	>0.9999	3.64	0.05	4.04	0.13	0.0028					
		70	3.96	0.11	4.01	0.09	>0.9999	3.61	0.05	3.98	0.12	0.0092					
		75	3.95	0.11	4.00	0.09	>0.9999	3.65	0.06	4.00	0.10	0.015					
		80	3.93	0.10	3.98	0.09	>0.9999	3.62	0.06	3.96	0.10	0.0219					
	**12**	10															
		15															
		20															
		25															
		30	4.47	0.24	4.44	0.28	>0.9999										
		35	4.20	0.13	4.42	0.19	0.991										
		40	4.24	0.18	4.35	0.15	>0.9999	3.83	0.05	4.22	0.12	0.0278					
		45	4.21	0.14	4.21	0.11	>0.9999	3.82	0.05	4.25	0.13	0.004					
		50	4.29	0.18	4.20	0.10	>0.9999	3.77	0.05	4.09	0.13	0.0672					
		55	4.04	0.13	4.20	0.13	0.997	3.74	0.04	4.09	0.11	0.0208					

		60	4.10	0.16	4.09	0.11	>0.9999	3.70	0.05	4.00	0.12	0.078					
		65	4.03	0.14	4.10	0.10	>0.9999	3.67	0.05	4.12	0.16	0.0005					
		70	3.98	0.14	4.08	0.11	>0.9999	3.68	0.05	4.08	0.12	0.0028					
		75	3.94	0.14	4.01	0.10	>0.9999	3.66	0.05	4.07	0.10	0.002					
		80	3.93	0.13	3.98	0.10	>0.9999	3.69	0.05	4.02	0.10	0.0283					
	**16**	10															
		15															
		20															
		25															
		30	4.69	0.32	4.48	0.31	1										
		35	4.52	0.24	4.41	0.17	>0.9999										
		40	4.34	0.18	4.36	0.16	>0.9999	3.93	0.07	4.32	0.13	0.1102					
		45	4.29	0.16	4.27	0.12	>0.9999	3.87	0.06	4.17	0.11	0.2608					
		50	4.33	0.16	4.19	0.13	1	3.81	0.05	4.26	0.17	0.0084					
		55	4.27	0.15	4.19	0.12	>0.9999	3.82	0.06	4.17	0.14	0.0499					
		60	4.20	0.16	4.13	0.10	>0.9999	3.79	0.05	4.09	0.13	0.142					
		65	4.20	0.17	4.13	0.11	>0.9999	3.76	0.05	4.13	0.13	0.0374					
		70	4.13	0.16	4.09	0.12	>0.9999	3.73	0.05	4.07	0.13	0.058					
		75	4.11	0.16	4.04	0.11	>0.9999	3.71	0.05	4.11	0.16	0.0157					
		80	4.01	0.15	3.99	0.10	>0.9999	3.68	0.06	4.09	0.13	0.0132					
	**24**	10															
		15															
		20															
		25															
		30															
		35															
		40	4.45	0.07	4.14	0.21	0.98										
		45	4.31	0.09	4.25	0.19	>0.9999	4.02	0.11	4.52	0.16	0.0498					

		50	4.35	0.24	4.21	0.15	0.999	3.84	0.10	4.12	0.09	0.5443					
		55	4.36	0.21	4.15	0.12	0.967	3.83	0.08	4.15	0.16	0.2319					
		60	4.06	0.10	3.92	0.16	0.997	3.82	0.06	4.09	0.17	0.4307					
		65	3.96	0.11	4.15	0.15	0.976	3.79	0.07	4.06	0.15	0.3506					
		70	4.01	0.13	3.96	0.08	>0.9999	3.79	0.06	3.99	0.14	0.7042					
		75	4.04	0.18	3.93	0.10	0.999	3.77	0.06	3.96	0.14	0.7873					
		80	3.95	0.17	3.89	0.09	>0.9999	3.73	0.06	3.93	0.13	0.7606					
	**32**	10															
		15															
		20															
		25															
		30															
		35															
		40															
		45	4.67	0.39	4.12	0.26	0.607	3.82	0.10	4.17	0.14	0.8307					
		50	4.37	0.29	4.09	0.34	0.986	3.85	0.10	4.03	0.14	0.9772					
		55	4.09	0.19	4.08	0.21	>0.9999	3.75	0.09	4.02	0.09	0.6524					
		60	4.04	0.18	3.99	0.15	>0.9999	3.83	0.10	4.09	0.18	0.6091					
		65	4.20	0.19	4.00	0.10	0.978	3.82	0.09	3.98	0.16	0.9378					
		70	4.07	0.15	3.89	0.06	0.987	3.71	0.07	3.95	0.17	0.6291					
		75	3.97	0.13	3.96	0.14	>0.9999	3.67	0.08	3.92	0.15	0.5461					
		80	3.94	0.14	3.88	0.13	>0.9999	3.58	0.07	3.95	0.14	0.1226					
**IV**	**8**	10															
		15															
		20															
		25															
		30															
		35															
		40	5.27	0.19	5.57	0.35	0.958										
		45	5.38	0.16	5.53	0.26	0.999	4.75	0.07	5.22	0.18	0.1053					
		50	5.05	0.16	5.36	0.21	0.858	4.69	0.08	5.17	0.13	0.0153					
		55	5.08	0.16	5.19	0.16	1	4.66	0.08	5.19	0.14	0.0012					
		60	5.00	0.16	5.09	0.15	>0.9999	4.61	0.07	5.22	0.14	<0.0001					

		65	5.00	0.13	5.11	0.12	1	4.55	0.08	5.14	0.13	0.0001					
		70	4.94	0.14	5.17	0.15	0.949	4.53	0.07	5.12	0.12	0.0001					
		75	4.93	0.15	5.04	0.12	1	4.54	0.07	5.08	0.12	0.0006					
		80	4.95	0.14	5.01	0.18	>0.9999	4.51	0.07	5.03	0.12	0.001					
	**12**	10															
		15															
		20															
		25															
		30	5.60	0.26	5.67	0.40	>0.9999										
		35	5.33	0.20	5.69	0.27	0.951										
		40	5.31	0.19	5.54	0.23	0.997	4.85	0.07	5.36	0.20	0.0582					
		45	5.24	0.15	5.37	0.19	>0.9999	4.79	0.08	5.40	0.21	0.0018					
		50	5.29	0.17	5.35	0.18	>0.9999	4.69	0.08	5.27	0.16	0.003					
		55	5.17	0.17	5.33	0.16	0.999	4.74	0.07	5.28	0.18	0.0039					
		60	5.11	0.18	5.27	0.15	1	4.68	0.07	5.15	0.16	0.0193					
		65	5.03	0.17	5.19	0.13	1	4.57	0.07	5.18	0.15	0.0006					
		70	4.92	0.17	5.13	0.16	0.993	4.51	0.07	5.08	0.13	0.0018					
		75	4.86	0.18	5.11	0.15	0.981	4.52	0.06	5.14	0.14	0.0005					
		80	4.81	0.17	5.10	0.14	0.941	4.56	0.08	5.04	0.13	0.0125					
	**16**	10															
		15															
		20															
		25															
		30	5.65	0.27	5.59	0.44	>0.9999										
		35	5.57	0.22	5.56	0.23	>0.9999										
		40	5.33	0.27	5.57	0.22	0.998	4.98	0.09	5.29	0.17	0.806					
		45	5.39	0.22	5.46	0.20	>0.9999	4.90	0.08	5.20	0.17	0.658					
		50	5.49	0.26	5.33	0.16	>0.9999	4.83	0.07	5.39	0.20	0.0159					
		55	5.33	0.23	5.29	0.19	>0.9999	4.79	0.08	5.32	0.18	0.0143					
		60	5.30	0.19	5.32	0.17	>0.9999	4.75	0.07	5.23	0.17	0.0403					

		65	5.16	0.18	5.29	0.16	>0.9999	4.71	0.08	5.23	0.16	0.0159					
		70	5.16	0.20	5.24	0.17	>0.9999	4.66	0.09	5.20	0.17	0.0112					
		75	5.06	0.20	5.15	0.17	>0.9999	4.62	0.09	5.19	0.18	0.0075					
		80	4.95	0.21	5.09	0.16	>0.9999	4.57	0.09	5.08	0.17	0.0252					
	**24**	10															
		15															
		20															
		25															
		30															
		35															
		40	5.58	0.20	5.27	0.31	0.999										
		45	5.75	0.27	5.32	0.36	0.958	5.01	0.14	5.37	0.20	0.5554					
		50	5.58	0.36	5.16	0.19	0.86	4.91	0.11	5.05	0.14	0.9942					
		55	5.42	0.26	4.99	0.14	0.761	4.77	0.10	5.09	0.19	0.465					
		60	5.43	0.28	4.90	0.17	0.495	4.74	0.08	5.02	0.18	0.6018					
		65	5.31	0.24	5.00	0.15	0.95	4.71	0.07	4.98	0.17	0.6381					
		70	5.04	0.19	4.97	0.12	>0.9999	4.65	0.08	4.91	0.18	0.6738					
		75	5.10	0.22	4.92	0.13	0.999	4.61	0.07	4.95	0.17	0.3346					
		80	4.97	0.22	4.76	0.09	0.995	4.54	0.07	5.00	0.19	0.0522					
	**32**	10															
		15															
		20															
		25															
		30															
		35															
		40															
		45	5.84	0.42	5.06	0.29	0.534	4.83	0.10	5.19	0.20	0.8706					
		50	5.49	0.27	4.97	0.33	0.913	4.82	0.09	4.92	0.21	0.9999					
		55	5.03	0.22	4.93	0.18	>0.9999	4.75	0.08	4.94	0.11	0.9407					
		60	4.92	0.19	4.88	0.13	>0.9999	4.84	0.11	5.03	0.23	0.9399					
		65	5.31	0.31	4.90	0.13	0.823	4.82	0.12	5.01	0.20	0.9151					
		70	5.26	0.29	5.02	0.12	0.992	4.67	0.07	4.94	0.19	0.6267					
		75	5.17	0.23	4.78	0.15	0.839	4.66	0.08	4.94	0.17	0.553					

Peak	Stimulus (kHz)	Intensity (dB SPL)	Mean	SEM	Mean	SEM	P value	Mean	SEM	Mean	SEM	P value
		80	5.05	0.24	4.71	0.16	0.925	4.51	0.08	4.73	0.10	0.8262
		Interpeak Latency (ms)										
			DMSO/CTL 4 wk		BLZ/PLX 4 wk			DMSO/CTL 7 wk		BLZ/PLX 7 wk		
**Peak**	**Stimulus (kHz)**	Intensity (dB SPL)	Mean	SEM	Mean	SEM	P value	Mean	SEM	Mean	SEM	P value
**I-II**	**8**	10										
		15										
		20										
		25										
		30										
		35										
		40	1.09	0.09	1.18	0.09	0.996					
		45	1.09	0.06	1.30	0.09	0.465	1.06	0.05	1.21	0.09	0.809
		50	1.11	0.07	1.12	0.06	>0.9999	1.10	0.05	1.07	0.05	>0.9999
		55	1.24	0.08	1.15	0.06	0.99	1.08	0.05	1.01	0.06	0.9817
		60	1.23	0.08	1.20	0.07	>0.9999	1.10	0.05	1.06	0.07	0.9992
		65	1.22	0.08	1.17	0.07	>0.9999	1.11	0.04	1.13	0.07	>0.9999
		70	1.22	0.08	1.19	0.06	>0.9999	1.11	0.04	1.11	0.07	>0.9999
		75	1.19	0.09	1.23	0.04	>0.9999	1.09	0.04	1.16	0.07	0.9788
		80	1.18	0.09	1.22	0.03	>0.9999	1.11	0.04	1.12	0.08	>0.9999
	**12**	10										
		15										
		20										
		25										
		30	1.31	0.09	1.32	0.04	>0.9999					
		35	1.21	0.08	1.19	0.05	>0.9999	1.12	0.04	1.11	0.02	>0.9999
		40	1.20	0.10	1.18	0.05	>0.9999	1.09	0.04	1.17	0.06	0.9856
		45	1.15	0.11	1.22	0.05	1	1.09	0.05	1.12	0.04	>0.9999
		50	1.17	0.08	1.17	0.05	>0.9999	1.09	0.05	1.09	0.05	>0.9999
		55	1.15	0.08	1.17	0.05	>0.9999	1.13	0.04	1.03	0.06	0.8302

		60	1.22	0.10	1.21	0.06	>0.9999	1.13	0.04	1.05	0.07	0.9481					
		65	1.17	0.10	1.18	0.06	>0.9999	1.14	0.03	1.17	0.07	>0.9999					
		70	1.24	0.09	1.23	0.03	>0.9999	1.14	0.03	1.17	0.07	0.9998					
		75	1.26	0.08	1.20	0.03	1	1.13	0.03	1.17	0.06	0.9994					
		80	1.24	0.07	1.18	0.03	1	1.10	0.03	1.19	0.03	0.835					
	**16**	10															
		15															
		20															
		25															
		30	1.22	0.09	1.24	0.10	>0.9999										
		35	1.12	0.05	1.15	0.08	>0.9999										
		40	1.07	0.09	1.14	0.07	>0.9999	1.09	0.04	1.09	0.06	>0.9999					
		45	1.04	0.10	1.05	0.06	>0.9999	1.11	0.04	1.07	0.06	0.9992					
		50	1.18	0.12	1.04	0.07	0.957	1.09	0.05	1.03	0.05	0.994					
		55	1.23	0.11	1.06	0.08	0.819	1.10	0.05	1.02	0.04	0.9544					
		60	1.23	0.11	1.19	0.07	>0.9999	1.10	0.05	1.03	0.06	0.9715					
		65	1.26	0.11	1.18	0.06	1	1.12	0.04	1.14	0.06	>0.9999					
		70	1.23	0.10	1.12	0.06	0.988	1.11	0.04	1.20	0.06	0.8436					
		75	1.27	0.09	1.13	0.05	0.944	1.12	0.04	1.17	0.06	0.9963					
		80	1.23	0.09	1.10	0.05	0.961	1.10	0.04	1.20	0.04	0.7865					
	**24**	10															
		15															
		20															
		25															
		30															
		35															
		40	1.01	0.06	0.92	0.06	>0.9999										
		45	1.02	0.05	0.96	0.06	>0.9999	0.96	0.06	1.03	0.09	0.9971					
		50	1.21	0.16	0.91	0.04	0.269	0.96	0.08	0.86	0.03	0.973					
		55	1.19	0.17	0.91	0.04	0.272	0.93	0.07	0.88	0.06	0.9996					
		60	1.07	0.11	0.91	0.05	0.907	0.92	0.07	0.85	0.04	0.9964					

		65	1.06	0.10	0.93	0.07	0.962	1.02	0.08	0.83	0.04	0.3153					
		70	1.01	0.09	0.95	0.07	1	1.02	0.07	0.82	0.04	0.2175					
		75	1.00	0.08	0.98	0.08	>0.9999	1.04	0.07	0.89	0.06	0.5995					
		80	0.96	0.09	0.95	0.07	>0.9999	1.00	0.07	0.95	0.06	0.9996					
	**32**	10															
		15															
		20															
		25															
		30															
		35															
		40															
		45	1.16	0.26	0.89	0.05	0.922	0.85	0.02	0.77	0.02	0.9978					
		50	1.14	0.24	0.87	0.08	0.922	0.86	0.06	0.89	0.08	>0.9999					
		55	0.90	0.16	0.90	0.03	>0.9999	0.77	0.02	0.82	0.03	0.9994					
		60	0.86	0.15	0.99	0.10	0.995	0.81	0.04	0.86	0.05	0.9988					
		65	0.92	0.15	0.95	0.09	>0.9999	0.83	0.05	0.83	0.04	>0.9999					
		70	1.00	0.09	0.89	0.08	0.997	0.86	0.06	0.80	0.04	0.9929					
		75	0.98	0.10	0.94	0.10	>0.9999	0.86	0.06	0.77	0.04	0.8627					
		80	0.94	0.10	0.92	0.10	>0.9999	0.90	0.08	0.77	0.05	0.5378					
**II-III**	**8**	10															
		15															
		20															
		25															
		30															
		35															
		40	0.95	0.09	0.81	0.08	0.952										
		45	1.08	0.09	0.87	0.04	0.475	0.83	0.03	0.76	0.03	0.9985					
		50	0.97	0.09	1.10	0.13	0.885	0.77	0.04	0.99	0.05	0.0883					
		55	0.94	0.05	0.98	0.07	>0.9999	0.76	0.04	1.15	0.09	<0.0001					
		60	0.91	0.05	0.92	0.07	>0.9999	0.74	0.04	1.07	0.09	0.0002					
		65	0.92	0.06	0.95	0.07	>0.9999	0.79	0.03	1.00	0.11	0.063					

		70	0.96	0.06	0.94	0.07	>0.9999	0.81	0.03	1.00	0.11	0.1036					
		75	0.99	0.07	0.92	0.05	0.996	0.86	0.04	1.02	0.05	0.2495					
		80	0.99	0.08	0.95	0.04	>0.9999	0.83	0.03	1.05	0.05	0.0465					
	**12**	10															
		15															
		20															
		25															
		30	1.08	0.12	1.02	0.14	>0.9999										
		35	1.01	0.05	1.14	0.11	0.992										
		40	1.07	0.11	1.11	0.10	>0.9999	0.85	0.03	1.01	0.07	0.3029					
		45	1.14	0.12	0.98	0.07	0.924	0.85	0.04	1.07	0.06	0.0246					
		50	1.13	0.11	1.04	0.06	0.998	0.85	0.04	1.03	0.06	0.1021					
		55	0.95	0.08	1.09	0.09	0.905	0.83	0.03	1.10	0.05	0.0006					
		60	1.03	0.08	0.98	0.07	>0.9999	0.83	0.03	1.04	0.06	0.0174					
		65	1.01	0.09	1.02	0.07	>0.9999	0.80	0.03	1.07	0.10	0.0005					
		70	0.95	0.06	0.98	0.06	>0.9999	0.84	0.04	1.06	0.06	0.0062					
		75	0.89	0.07	0.96	0.06	1	0.83	0.04	1.07	0.05	0.0019					
		80	0.91	0.06	0.98	0.06	1	0.88	0.04	1.04	0.04	0.1393					
	**16**	10															
		15															
		20															
		25															
		30	1.43	0.26	1.18	0.20	0.93										
		35	1.33	0.20	1.18	0.10	0.986										
		40	1.26	0.12	1.21	0.10	>0.9999	0.93	0.05	1.12	0.10	0.3765					
		45	1.27	0.13	1.21	0.08	>0.9999	0.88	0.03	1.12	0.07	0.0403					
		50	1.16	0.09	1.20	0.10	>0.9999	0.88	0.03	1.23	0.09	0.0002					
		55	1.03	0.06	1.21	0.12	0.851	0.90	0.04	1.18	0.07	0.0025					
		60	1.02	0.06	1.05	0.08	>0.9999	0.89	0.03	1.13	0.07	0.0189					
		65	1.06	0.09	1.08	0.07	>0.9999	0.88	0.04	1.10	0.08	0.0325					

		70	1.04	0.08	1.11	0.06	>0.9999	0.89	0.04	1.04	0.08	0.3778					
		75	1.02	0.07	1.07	0.07	>0.9999	0.89	0.04	1.12	0.10	0.0208					
		80	0.97	0.06	1.07	0.07	0.999	0.91	0.04	1.10	0.06	0.1151					
	**24**	10															
		15															
		20															
		25															
		30															
		35															
		40	1.48	0.04	1.32	0.06	0.999										
		45	1.33	0.14	1.42	0.08	>0.9999	1.15	0.08	1.34	0.12	0.7724					
		50	1.31	0.14	1.44	0.07	0.997	1.07	0.08	1.37	0.05	0.1317					
		55	1.32	0.15	1.42	0.04	0.999	1.12	0.09	1.43	0.07	0.055					
		60	1.16	0.11	1.22	0.13	>0.9999	1.14	0.08	1.43	0.08	0.0821					
		65	1.07	0.09	1.41	0.13	0.285	1.01	0.08	1.45	0.07	0.0006					
		70	1.19	0.11	1.26	0.08	>0.9999	1.03	0.07	1.42	0.08	0.0045					
		75	1.24	0.16	1.21	0.12	>0.9999	1.01	0.07	1.35	0.09	0.0195					
		80	1.20	0.15	1.21	0.10	>0.9999	1.03	0.06	1.26	0.11	0.2655					
	**32**	10															
		15															
		20															
		25															
		30															
		35															
		40															
		45	1.59	0.32	1.41	0.12	0.99	1.16	0.07	1.40	0.12	0.8376					
		50	1.32	0.15	1.43	0.19	1	1.18	0.07	1.33	0.08	0.9289					
		55	1.39	0.07	1.38	0.11	>0.9999	1.19	0.07	1.35	0.05	0.769					
		60	1.40	0.06	1.20	0.14	0.879	1.23	0.07	1.41	0.08	0.623					
		65	1.41	0.07	1.26	0.09	0.964	1.22	0.07	1.36	0.08	0.8673					
		70	1.21	0.10	1.18	0.07	>0.9999	1.09	0.07	1.39	0.09	0.0457					

		75	1.09	0.10	1.23	0.15	0.973	1.07	0.08	1.37	0.10	0.0382					
		80	1.13	0.12	1.15	0.11	>0.9999	0.98	0.07	1.44	0.08	0.0002					
**III-IV**	**8**	10															
		15															
		20															
		25															
		30	1.32	0.11	1.23	0.15	>0.9999										
		35	1.13	0.11	1.27	0.09	0.991										
		40	1.07	0.11	1.20	0.09	0.99										
		45	1.03	0.10	1.16	0.10	0.984	0.98	0.04	1.03	0.03	0.9986					
		50	1.00	0.09	1.15	0.11	0.868	0.98	0.03	1.09	0.06	0.661					
		55	1.12	0.13	1.13	0.07	>0.9999	1.02	0.04	0.99	0.04	0.9999					
		60	1.01	0.06	1.18	0.08	0.779	1.00	0.04	1.12	0.05	0.3293					
		65	1.00	0.05	1.09	0.07	0.999	0.91	0.04	1.10	0.03	0.0301					
		70	0.94	0.03	1.05	0.07	0.985	0.91	0.04	1.14	0.04	0.0028					
		75	0.92	0.06	1.10	0.08	0.754	0.89	0.04	1.08	0.04	0.0205					
		80	0.88	0.07	1.11	0.07	0.367	0.88	0.04	1.07	0.04	0.023					
	**12**	10															
		15															
		20															
		25															
		30	0.96	0.16	1.11	0.14	0.998										
		35	1.05	0.09	1.16	0.07	0.999										
		40	0.99	0.13	1.21	0.07	0.687	1.02	0.05	1.13	0.11	0.9355					
		45	1.10	0.10	1.19	0.10	0.999	0.96	0.05	1.15	0.10	0.1819					
		50	1.15	0.12	1.14	0.07	>0.9999	0.92	0.05	1.18	0.05	0.0093					
		55	1.12	0.10	1.15	0.09	>0.9999	0.99	0.04	1.19	0.07	0.0844					
		60	1.10	0.06	1.20	0.10	0.998	0.98	0.04	1.16	0.06	0.1747					
		65	0.96	0.06	1.17	0.10	0.586	0.92	0.05	1.06	0.04	0.3795					
		70	1.03	0.06	1.15	0.08	0.983	0.85	0.04	1.00	0.05	0.3958					
		75	0.96	0.06	1.11	0.08	0.906	0.88	0.04	1.07	0.06	0.1044					
		80	0.93	0.07	1.11	0.08	0.823	0.87	0.06	1.02	0.05	0.2988					
	**16**	10															
		15															

		20															
		25															
		30															
		35															
		40	1.23	0.15	1.13	0.14	0.998	1.10	0.06	0.95	0.06	0.7472					
		45	1.24	0.12	1.16	0.18	1	1.02	0.06	1.02	0.05	>0.9999					
		50	1.23	0.11	0.96	0.07	0.147	1.02	0.04	1.13	0.05	0.8225					
		55	1.12	0.11	0.84	0.04	0.102	0.98	0.05	1.16	0.05	0.1663					
		60	1.09	0.06	0.98	0.09	0.963	0.96	0.04	1.14	0.05	0.2018					
		65	0.96	0.05	0.85	0.05	0.948	0.94	0.04	1.11	0.06	0.2786					
		70	1.01	0.07	1.01	0.05	>0.9999	0.93	0.06	1.13	0.06	0.089					
		75	0.96	0.05	0.99	0.06	>0.9999	0.91	0.06	1.08	0.06	0.2609					
		80	0.93	0.06	0.88	0.06	1	0.89	0.06	0.99	0.05	0.8735					
	**24**	10															
		15															
		20															
		25															
		30															
		35															
		40	1.12	0.13	1.13	0.14	>0.9999										
		45	1.45	0.29	1.16	0.18	0.903	0.98	0.06	0.85	0.10	0.8077					
		50	1.23	0.14	0.96	0.07	0.716	1.06	0.10	0.91	0.05	0.6357					
		55	1.06	0.07	0.84	0.04	0.837	0.94	0.07	0.94	0.04	>0.9999					
		60	1.37	0.23	0.98	0.09	0.174	0.92	0.04	0.93	0.04	>0.9999					
		65	1.29	0.19	0.85	0.05	0.064	0.92	0.05	0.91	0.04	>0.9999					
		70	1.03	0.08	1.01	0.05	>0.9999	0.87	0.05	0.91	0.05	0.998					
		75	1.05	0.13	0.99	0.06	>0.9999	0.85	0.05	0.99	0.06	0.461					
		80	1.02	0.12	0.88	0.06	0.983	0.81	0.05	1.07	0.06	0.0076					
	**32**	10															
		15															
		20															
		25															
		30															
		35															

		40															
		45	1.17	0.11	0.93	0.06	0.96	1.01	0.08	1.03	0.08	>0.9999					
		50	1.12	0.10	0.88	0.06	0.956	0.98	0.06	0.89	0.09	0.9964					
		55	0.95	0.07	0.86	0.09	1	0.99	0.05	0.92	0.04	0.9978					
		60	0.88	0.05	0.89	0.08	>0.9999	1.01	0.06	0.94	0.06	0.9947					
		65	1.11	0.17	0.90	0.06	0.858	0.97	0.08	1.03	0.04	0.9983					
		70	1.19	0.17	1.10	0.09	0.999	0.97	0.08	0.99	0.05	>0.9999					
		75	1.20	0.16	0.82	0.06	0.145	0.99	0.09	1.01	0.05	>0.9999					
		80	1.09	0.16	0.83	0.06	0.601	0.93	0.07	0.91	0.05	>0.9999					
**I-IV**	**8**	10															
		15															
		20															
		25															
		30															
		35															
		40	3.20	0.17	3.44	0.27	0.981										
		45	3.33	0.15	3.43	0.19	1	2.87	0.06	3.00	0.13	0.9789					
		50	3.08	0.16	3.31	0.17	0.924	2.84	0.07	3.12	0.10	0.1771					
		55	3.15	0.15	3.18	0.12	>0.9999	2.85	0.06	3.15	0.11	0.0639					
		60	3.13	0.16	3.13	0.12	>0.9999	2.84	0.06	3.25	0.11	0.0013					
		65	3.17	0.11	3.20	0.10	>0.9999	2.82	0.06	3.23	0.10	0.0014					
		70	3.15	0.12	3.29	0.13	0.997	2.83	0.06	3.25	0.09	0.0009					
		75	3.15	0.13	3.19	0.11	>0.9999	2.84	0.06	3.27	0.10	0.0009					
		80	3.19	0.13	3.21	0.14	>0.9999	2.83	0.06	3.24	0.09	0.0012					
	**12**	10															
		15															
		20															
		25															
		30	3.56	0.21	3.57	0.27	>0.9999										
		35	3.29	0.18	3.59	0.21	0.961										
		40	3.29	0.18	3.48	0.18	0.998	2.96	0.06	3.31	0.18	0.1644					
		45	3.24	0.15	3.36	0.16	>0.9999	2.91	0.06	3.34	0.17	0.0075					

		50	3.29	0.16	3.37	0.15	>0.9999	2.86	0.07	3.28	0.13	0.007					
		55	3.22	0.16	3.39	0.13	0.996	2.95	0.06	3.33	0.14	0.0161					
		60	3.26	0.16	3.37	0.12	>0.9999	2.94	0.06	3.25	0.12	0.0844					
		65	3.18	0.16	3.30	0.10	>0.9999	2.85	0.06	3.30	0.11	0.0014					
		70	3.13	0.15	3.26	0.13	1	2.81	0.05	3.23	0.10	0.0036					
		75	3.08	0.17	3.26	0.12	0.995	2.83	0.04	3.32	0.10	0.0004					
		80	3.04	0.16	3.28	0.12	0.955	2.85	0.07	3.25	0.10	0.0063					
	**16**	10															
		15															
		20															
		25															
		30	3.62	0.21	3.53	0.33	>0.9999										
		35	3.50	0.20	3.48	0.18	>0.9999										
		40	3.33	0.25	3.57	0.16	0.995	3.07	0.07	3.19	0.14	0.9986					
		45	3.41	0.20	3.45	0.17	>0.9999	3.02	0.07	3.17	0.14	0.9574					
		50	3.50	0.23	3.38	0.14	>0.9999	2.99	0.06	3.39	0.16	0.0403					
		55	3.37	0.21	3.39	0.16	>0.9999	2.97	0.06	3.36	0.14	0.0336					
		60	3.35	0.17	3.43	0.14	>0.9999	2.96	0.06	3.30	0.13	0.0901					
		65	3.27	0.16	3.43	0.13	1	2.95	0.06	3.35	0.12	0.0236					
		70	3.31	0.18	3.38	0.14	>0.9999	2.93	0.08	3.37	0.13	0.0095					
		75	3.24	0.18	3.31	0.15	>0.9999	2.92	0.08	3.37	0.14	0.0073					
		80	3.14	0.19	3.27	0.14	>0.9999	2.90	0.08	3.29	0.13	0.0349					
	**24**	10															
		15															
		20															
		25															
		30															
		35															
		40	3.62	0.20	3.37	0.23	0.999										
		45	3.79	0.23	3.49	0.30	0.988	3.07	0.08	3.26	0.15	0.8856					

		50	3.65	0.27	3.31	0.14	0.888	3.08	0.06	3.19	0.11	0.9933					
		55	3.58	0.23	3.17	0.09	0.654	2.98	0.07	3.25	0.15	0.2944					
		60	3.60	0.25	3.11	0.12	0.385	2.97	0.06	3.21	0.13	0.4279					
		65	3.48	0.21	3.19	0.10	0.922	2.95	0.06	3.20	0.13	0.3794					
		70	3.22	0.17	3.21	0.09	>0.9999	2.92	0.06	3.15	0.14	0.4789					
		75	3.30	0.20	3.18	0.10	1	2.91	0.05	3.22	0.13	0.109					
		80	3.19	0.20	3.03	0.07	0.998	2.83	0.05	3.28	0.15	0.0039					
	**32**	10															
		15															
		20															
		25															
		30															
		35															
		40															
		45	3.92	0.40	3.24	0.19	0.487	3.01	0.06	3.21	0.19	0.9816					
		50	3.58	0.26	3.19	0.26	0.949	3.02	0.06	3.03	0.18	>0.9999					
		55	3.23	0.19	3.12	0.13	>0.9999	2.95	0.06	3.10	0.10	0.9616					
		60	3.14	0.16	3.08	0.09	>0.9999	3.05	0.08	3.20	0.18	0.9234					
		65	3.44	0.26	3.11	0.09	0.853	3.06	0.09	3.22	0.16	0.8805					
		70	3.40	0.23	3.18	0.10	0.988	2.91	0.06	3.18	0.14	0.3112					
		75	3.27	0.20	3.00	0.13	0.936	2.92	0.06	3.15	0.13	0.4793					
		80	3.19	0.20	2.90	0.10	0.918	2.82	0.08	3.03	0.08	0.6051					
